# Fringe GlcNAc-transferases differentially extend *O*-fucose on endogenous NOTCH1 in mouse activated T cells

**DOI:** 10.1016/j.jbc.2022.102064

**Published:** 2022-05-25

**Authors:** Kenjiroo Matsumoto, Vivek Kumar, Shweta Varshney, Alison V. Nairn, Atsuko Ito, Florian Pennarubia, Kelley W. Moremen, Pamela Stanley, Robert S. Haltiwanger

**Affiliations:** 1 Complex Carbohydrate Research Center, Department of Biochemistry and Molecular Biology, University of Georgia, Athens, Georgia, USA; 2 Department of Cell Biology, Albert Einstein College of Medicine, New York, New York, USA

**Keywords:** fringe, Notch, glycosylation, *O*-fucose, EGF repeats, mass spectrometry, T cells, BCS, bovine calf serum, cDNA, complementary DNA, CHO, Chinese hamster ovary, DLL1, Delta-like ligand, ECD, extracellular domain, EGF, epidermal growth factor-like, EIC, extracted ion chromatogram, ER, endoplasmic reticulum, FBS, fetal bovine serum, LBB, ligand-binding buffer, MEM, minimum essential medium α, mN1, mouse NOTCH1, MS, mass spectrometry, PE, phycoerythrin, PFA, paraformaldehyde, TBS, Tris-buffered saline

## Abstract

NOTCH1 is a transmembrane receptor that initiates a cell–cell signaling pathway controlling various cell fate specifications in metazoans. The addition of *O*-fucose by protein *O*-fucosyltransferase 1 (POFUT1) to epidermal growth factor-like (EGF) repeats in the NOTCH1 extracellular domain is essential for NOTCH1 function, and modification of *O*-fucose with GlcNAc by the Fringe family of glycosyltransferases modulates Notch activity. Prior cell-based studies showed that POFUT1 modifies EGF repeats containing the appropriate consensus sequence at high stoichiometry, while Fringe GlcNAc-transferases (LFNG, MFNG, and RFNG) modify *O*-fucose on only a subset of NOTCH1 EGF repeats. Previous *in vivo* studies showed that each FNG affects naïve T cell development. To examine Fringe modifications of NOTCH1 at a physiological level, we used mass spectral glycoproteomic methods to analyze *O*-fucose glycans of endogenous NOTCH1 from activated T cells obtained from mice lacking all Fringe enzymes or expressing only a single FNG. While most *O*-fucose sites were modified at high stoichiometry, only EGF6, EGF16, EGF26, and EGF27 were extended in WT T cells. Additionally, cell-based assays of NOTCH1 lacking fucose at each of those *O*-fucose sites revealed small but significant effects of LFNG on Notch-Delta binding in the EGF16 and EGF27 mutants. Finally, in activated T cells expressing only LFNG, MFNG, or RFNG alone, the extension of *O*-fucose with GlcNAc in the same EGF repeats was diminished, consistent with cooperative interactions when all three Fringes were present. The combined data open the door for the analysis of *O*-glycans on endogenous NOTCH1 derived from different cell types.

The diverse set of glycan structures found on proteins in mammalian cells (1, 2) are determined by many factors, including the specific complement of glycosyltransferases and glycosidases expressed in a given cell (3). This makes prediction of glycan structures based solely on mRNA levels in individual cells very difficult. However, recent advances in glycomics and glycoproteomics permit site-specific analysis of glycans on proteins (4, 5). Such studies are essential to understanding how glycans affect cell signaling pathways such as that initiated by Notch receptors (6, 7, 8, 9).

Notch receptors are activated by direct interactions with Delta-like ligand 1 or 4 (DLL1 or DLL4) or Jagged ligands (JAG1 and JAG2) expressed on adjacent cells (10). Four Notch receptors exist in mammals, NOTCH1-4. NOTCH1 (N1) and NOTCH2 (N2) both contain 36 tandem epidermal growth factor-like (EGF) repeats in their extracellular domain (ECD), many of which are modified by fucose *O*-linked to Ser/Thr (*O*-fucose) (4, 11, 12, 13). EGF repeats are small protein domains of ∼40 amino acids with a characteristic fold due to the presence of six conserved Cys residues that form three conserved disulfide bonds (C^1^-C^3^, C^2^-C^4^, and C^5^-C^6^). The *O*-fucose is added in the endoplasmic reticulum (ER) by protein *O*-fucosyltransferase 1 (POFUT1) to the sequence C^2^-X-X-X-X-(S/T)-C^3^ (Fig. 1*A*) (14, 15, 16). The *O*-fucose on selected EGF repeats can then be extended in the Golgi by one of three Fringe GlcNAc-transferases, Lunatic, Manic, or Radical Fringe (LFNG, MFNG, or RFNG), forming a GlcNAcβ1-3Fucose disaccharide (4, 12) (Fig. 1, *A* and *B*). The GlcNAc can be further extended with galactose and sialic acid (4).

**Figure 1:**
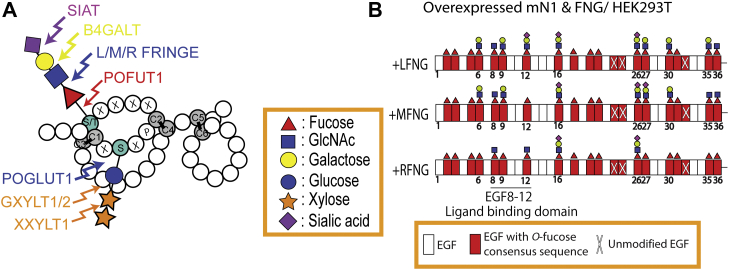
***O*-fucose modifies multiple N1 EGF repeats, but Fringes only modify a subset.***A*, diagram showing an EGF repeat with *O*-fucose and *O*-glucose glycans. *Circles* are amino acids, *gray circles* are the conserved cysteines forming disulfide bonds, and consensus sequences for *O*-fucose and *O*-glucose addition are included with *single letter codes*. Enzymes responsible for the modifications are indicated. Monosaccharide symbols are based on the Symbol Nomenclature for Glycans (60). *B*, summary of *O*-fucose glycans on mN1 EGF1-36 observed previously in HEK293T cells in the presence of overexpressed LFNG, MFNG, or RFNG (4, 13). Diagram shows the most abundant *O*-fucose glycan at each site. EGF repeats are represented as *rectangles*. EGF, epidermal growth factor-like.

Addition of *O*-fucose to Notch is essential for its function *in vivo*. KO of *Pofut1* in mice results in embryonic lethality with a Notch signaling defective phenotype (17). In contrast, Fringes regulate Notch activity. For instance, *Lfng*-null mice display somitogenesis defects due to disruption of Notch signaling (18, 19). *LFNG* mutations in humans cause a similar phenotype in autosomal recessive spondylocostal dysostosis 3 (OMIM #609813) (20). All three Fringe enzymes have been implicated in regulation of Notch activity in a variety of contexts. LFNG plays a role in angiogenesis (21) and kidney development (22), MFNG in ventricular chamber development in heart (23), and all three Fringes in bile duct remodeling (24) and B and T cell maturation and maintenance (25, 26, 27, 28).

Cell-based assays have been used to analyze how Fringe modifications affect Notch activity. While LFNG and MFNG both enhance N1 signaling from DLL1 but inhibit signaling from JAG1, RFNG enhances signaling from both (4, 11). Mass spectral analysis of the N1 ECD overexpressed in HEK293T cells in the presence or absence of exogenous Fringes showed that the majority of EGF repeats with the *O*-fucose consensus sequence are modified with *O*-fucose at high stoichiometry, but Fringes modify *O*-fucose only on some of those EGF repeats. In particular, LFNG modified *O*-fucose on N1 EGF repeats 6, 8, 9, 12, 16, 26, 27, 30, 35, and 36; MFNG modified the same N1 EGF repeats except for EGF12; and RFNG modified a subset of these EGF repeats 8, 12, 16, and 26 (4, 13) (Fig. 1*B*). Elimination of single *O*-fucose sites independently by mutagenesis revealed that Fringe modifications on EGF8 and EGF12 (both in the ligand-binding domain), enhance the ability of DLL1 to bind to and activate N1, while LFNG or MFNG modifications at EGF6 and EGF36 (not in the ligand-binding domain) inhibit N1 activation by JAG1 in a dominant fashion (4). The fact that RFNG did not modify EGF6 or EGF36 when overexpressed in HEK293T cells may provide an explanation for why RFNG does not inhibit activation of N1 by JAG1 in cell-based assays (Fig. 1*B*).

Addition of *O*-fucose to Notch is directly involved in Notch–ligand interactions. Elimination of *O*-fucose sites on N1 EGF repeats 8 or 12 reduces N1 binding to Delta and Jagged ligands, as well as reduces N1 activation by these ligands in cell-based assays (4, 11). N1-DLL4 fragments in a cocrystal provided a molecular explanation for these findings by showing that the *O*-fucose on N1 EGF12 (Thr466) is in direct contact with DLL4 at the N-terminal module termed MNNL (29). Modeling of a GlcNAc onto EGF12 *O*-fucose in the N1-DLL4 X-ray structure suggests enhanced interactions with the DLL4 MNNL domain (29). A subsequent N1-JAG1 fragment cocrystal structure showed that the *O*-fucose residues on both EGF8 and EGF12 were in direct contact with JAG1 (30), explaining the importance of these modifications for N1 activity.

The importance of the *O*-fucose on EGF12 of N1 in mice was shown by generating a Thr to Ala knock-in mutation in the endogenous mouse *Notch1* locus, eliminating the *O*-fucose site on EGF12 (termed *Notch1*12f) (31, 32). The early studies showed a hypomorphic allele with defects in T-cell differentiation (31) and development (33), while later studies showed that after multiple generations of backcrossing to C57Bl6 mice, the *Notch1*12f mutation resulted in embryonic lethality (32). Similarly, eliminating the *O*-fucose site on EGF12 of *Drosophila* Notch resulted in embryonic lethality with a neurogenic phenotype, and elimination of *O*-fucose on EGF8 or EGF12 reduced Fringe-dependent wing vein development (34).

All of the *O*-fucose site-mapping studies aforementioned used overexpressed portions of N1 ECD in cell lines with or without coexpressed Fringes (4, 13). Here, for the first time, we sought to analyze the site-specific *O*-fucose and Fringe modifications on endogenous N1 from a physiologically relevant system. We chose T cells due to the fact that Fringes affect their differentiation *in vivo* (28) and the ability to expand T cells and increase N1 expression upon activation by anti-CD3/CD28 *in vitro* (35). We used the mouse preT 2017 thymic lymphoma cell line (36, 37) to develop a N1 immunoprecipitation method, which was applied to anti-CD3/CD28–activated T cells derived from spleen of WT or *Fng* LMR (control), triple-Fringe KO (*Fng* tKO), and three double-KO lines expressing only one allele of a single Fringe gene (*Lfng*, *Mfng*, or *Rfng*) (28). Consistent with our previous site mapping studies using overexpressed N1 ECD, endogenous N1 from activated T cells was modified at many predicted sites with *O*-fucose. Surprisingly, we did not detect Fringe extension of *O-*fucose on EGF8 or 12 within the ligand-binding domain of N1 but rather on EGF 6, 16, 26, and 27. Mutation of the *O*-fucose sites on EGF16 and 27 reduced the ability of Fringe to enhance binding to DLLs using cell-based assays.

## Results

### A method to evaluate site-specific *O*-fucose and Fringe modifications of endogenous mouse NOTCH1

We previously developed methods to determine Fringe modifications of overexpressed, secreted mouse N1 (mN1) EGF1-36-Myc-His_6_ coexpressed with or without LFNG, MFNG, or RFNG in HEK293T cells, and purified from conditioned media using nitrilotriacetic acid–agarose chromatography (4). To analyze endogenous N1, we had to identify an antibody that could efficiently immunoprecipitate N1. The antibody also needed to be covalently coupled to magnetic beads so that elution from immunoglobulin G (IgG) did not interfere with downstream mass spectral analysis. Full-length mN1 was expressed in HEK293T cells with or without LFNG, and several commercial anti-N1 antibodies were tested for efficient immunoprecipitation. Fig. S1*A* shows that a sheep anti-mN1 polyclonal antibody against the ECD of mN1 efficiently immunoprecipitated N1, whether expressed with or without LFNG. N1 eluted from beads was analyzed by mass spectrometry (MS) as described in Experimental procedures. An extracted ion chromatogram (EIC) of the glycoforms of a peptide that includes the *O*-fucose site in EGF12 showed that mN1 overexpressed without cotransfected *Lfng* had only the monosaccharide *O*-fucose modification, while *Lfng* cotransfection resulted in extension of the *O*-fucose, generating *O*-fucose disaccharide and tetrasaccharide glycoforms (Fig. S1*B* and Table S1). Therefore, immunoprecipitation from cell lysates of full-length mN1, with or without modification by LFNG, could be used to evaluate Fringe modification in T cells using EICs.

**Figure 2:**
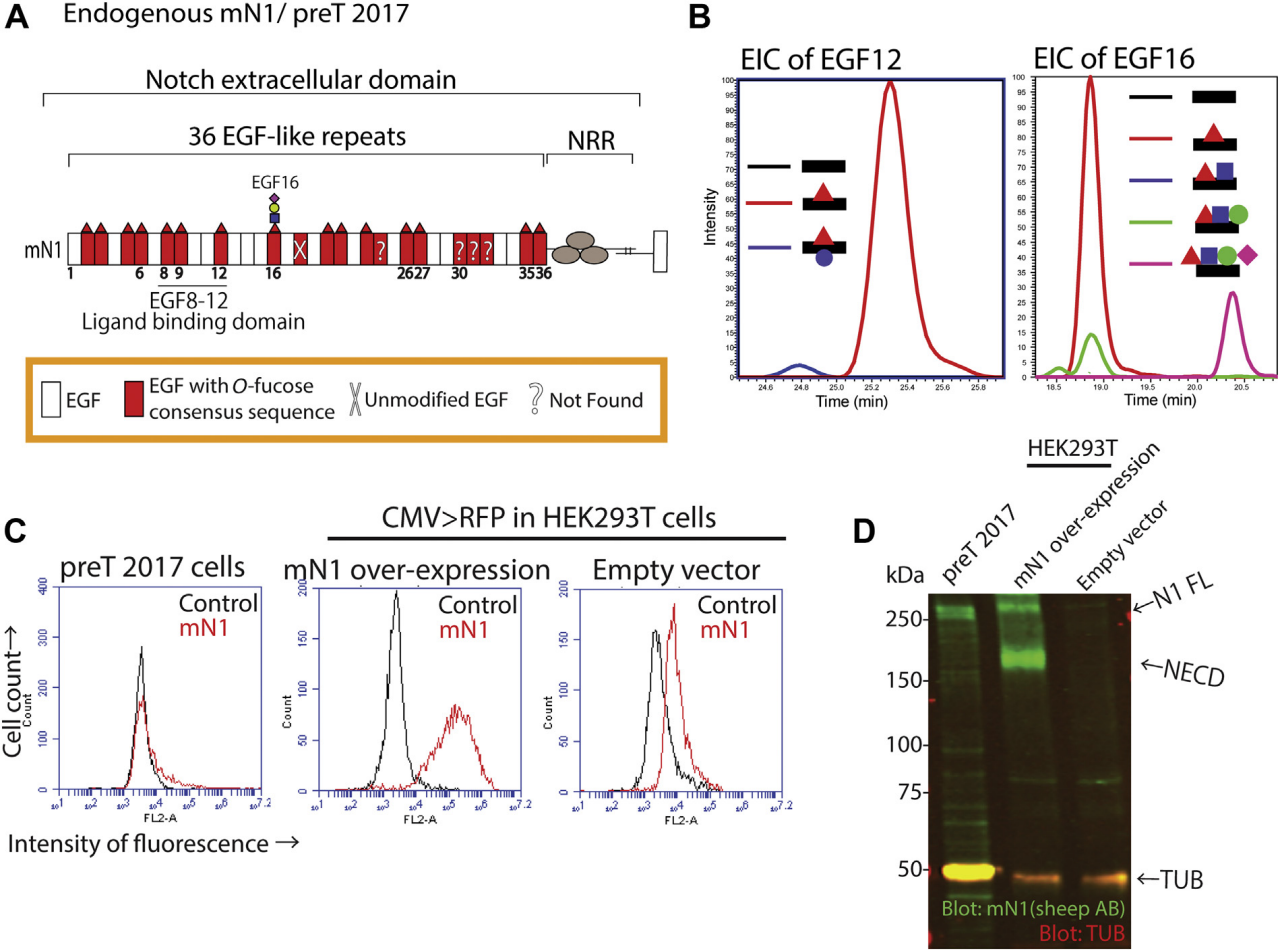
**Endogenous N1 from preT 2017 cells was modified by *O*-fucose at high stoichiometry, but only the EGF16 *O-*fucose was extended by Fringes.***A*, summary of *O*-fucose modifications identified by mass spectral analysis of endogenous mN1 isolated from preT 2017 cells. Most extended form of the *O*-fucose glycan detected is shown. Mass spectral data in Fig. S10 and Table S2. *B*, extracted ion chromatogram showing relative amounts of *O*-fucose peptide glycoforms from EGF12 and EGF16. *Black*, *red*, and *blue lines* indicate the unmodified, *O*-fucose monosaccharide, and both *O*-fucose and *O*-glucose monosaccharide glycoforms in the EGF12 panel, respectively. *Black*, *red*, *blue*, *green*, and *magenta lines* indicate the unmodified, monosaccharide, disaccharide, trisaccharide, and tetrasaccharide *O*-fucose glycoforms in the EGF16 panel, respectively. *C*, cell surface N1 expression measured by flow cytometry in preT 2017 cells or in HEK293T cells overexpressing (O/E) mN1 or empty vector (EV) control. Note that the mouse N1 antibody used here has slight crossreactivity with human N1. *D*, Western blot analysis of endogenous mN1 in preT 2017 lysate or overexpressed mN1 from HEK293T lysate with anti-N1 ECD antibody. ECD, extracellular domain; EGF, epidermal growth factor-like.

We tested the method to analyze endogenous mN1 using an immortalized mouse pre-T cell line, preT 2017 (36, 38). Endogenous mN1 in preT 2017 cell lysates was efficiently recovered by immunoprecipitation (Fig. S2*A*). The purified mN1 was divided into three portions, digested with trypsin, chymotrypsin, or V8 proteases, and the resulting peptides analyzed by MS, as described in Experimental procedures. All three proteases are required to identify peptides containing all of the *O*-fucose modification sites in mN1 (4). mN1 has 20 predicted *O*-fucose sites, and peptides containing 16 of these sites were identified (Fig. 2*A* and Table S2). Peptides containing the *O*-fucose consensus sequence from EGF24, EGF30, EGF31, and EGF32 were not detected (Fig. 2*A*). Peptide searches showed the *O*-fucose monosaccharide modification on all of the detected peptides with an *O*-fucose consensus site, except for EGF18, which was unmodified (Fig. 2*A* and Table S2). Only EGF16 had an extended *O*-fucose on a small proportion of *O*-fucosylated peptides. EICs of peptides from EGF12 and EGF16 showed a small amount of trisaccharide and tetrasaccharide on EGF16 but only *O*-fucose monosaccharide on EGF12 (Figs. 2*B* and S10). *O*-fucosylation at both sites was at high stoichiometry. Quantitative RT-PCR analysis of preT 2017 RNA and RNA from activated mouse T cells showed that both expressed equivalent levels of *Pofut1*, *Mfng*, and *Rfng* (Fig. S3). However, activated T cells had relatively more *Lfng* transcripts than preT 2017 cells (Fig. S3).

The *O*-fucosylated peptide from EGF12 of N1 from preT 2017 cells, TGPRcEIDVNEcI**S**NPcQNDA**T**cLDQIGEF, contains an *O*-glucose site (bold, underlined S) in addition to its *O*-fucose site (bold, underlined T). This peptide is usually modified by an *O*-fucose monosaccharide and an *O*-glucose trisaccharide in overexpressed N1 from HEK293T cells, as shown in Fig. S1*B* (4). However, the EGF12 peptide from preT 2017 cells was poorly modified with an *O*-glucose monosaccharide (Fig. 2*B*). *O*-glucose is added by POGLUT1, and *Poglut1* expression is two orders of magnitude lower than *Pofut1* expression in preT 2017 cells (Fig. S3). POGLUT1 contributes to N1 trafficking in mammalian cells (39). *Poglut1* KO leads to N1 accumulation in the ER and less cell surface N1 in some cell contexts (39). Very little N1 is expressed on the surface of preT 2017 cells (Fig. 2*C*). N1 is cleaved by furin in the *trans*-Golgi network generating a heterodimer held together by noncovalent bonds (40). This suggests that full-length (uncleaved) N1 is in the ER, whereas cell surface N1 is cleaved (Fig. 2*D*). N1 in preT 2017 cells appears to be mainly in the ER form (Fig. 2*D*). Since Fringes are Golgi-localized enzymes (20), localization of N1 in the ER could explain why there is so little Fringe modification of *O*-fucose residues in preT 2017 cells.

### Activated T cells have sufficient N1 for mass spectral analysis

**Figure 3:**
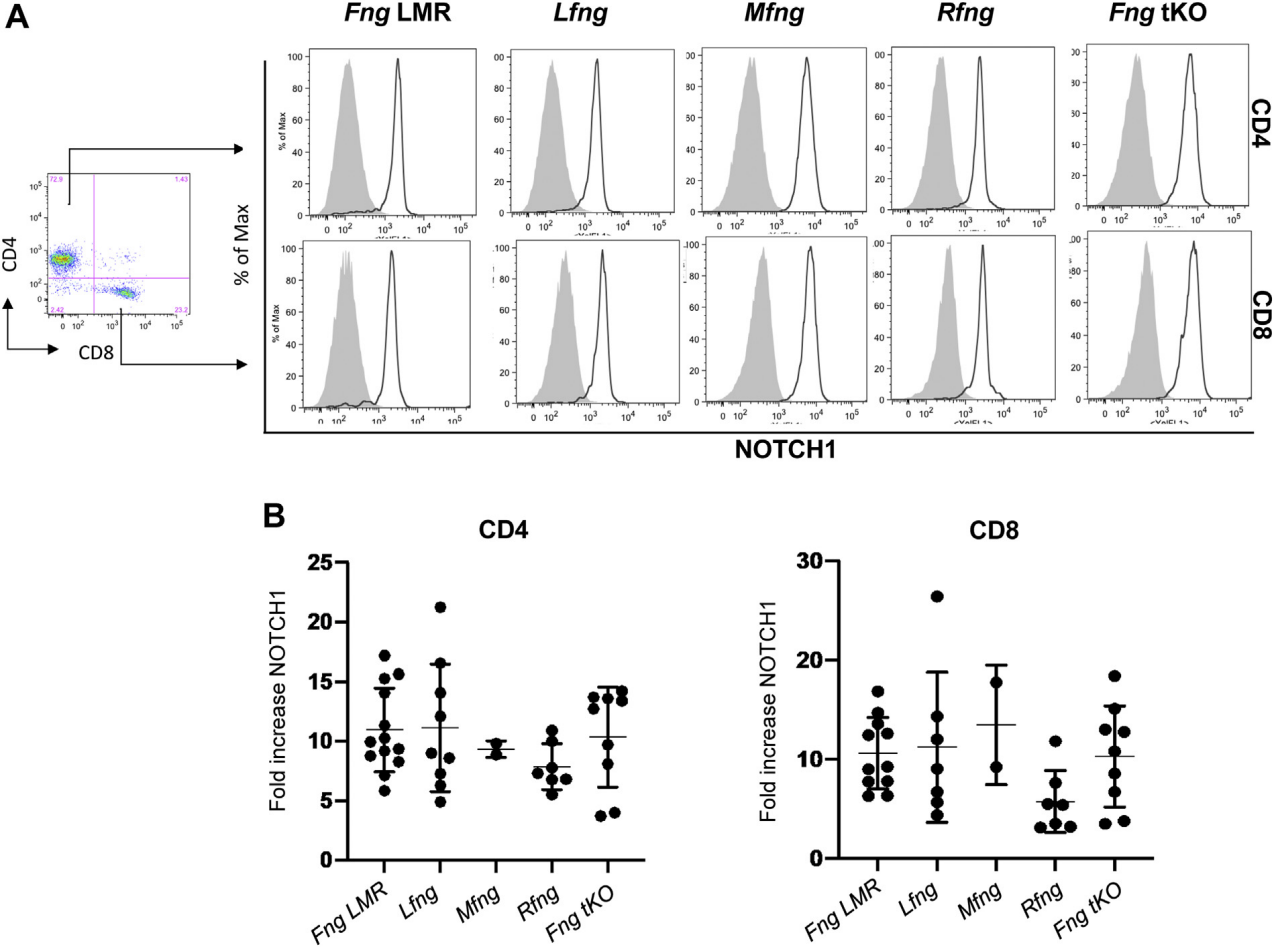
**T cells from *Fng* LMR and *Fng* mutant mice exhibit increased levels of cell surface N1 after activation in culture.***A*, representative flow cytometric dot plot showing CD4+ and CD8+ splenocytes used to analyze cell surface N1 expression. Flow cytometry profiles show N1 cell surface expression on CD4+-CD8+ enriched (*gray profile*) or anti-CD3/CD28 activated (*solid line*) T cells. *B*, fold-change in mean fluorescence index (MFI) of cell surface N1 in activated T cells compared to enriched T cells. *Fng* LMR mice express one allele of each *Fng* gene; *Lfng, Mfng*, or *Rfng* mice express one allele of the single *Fng* gene noted; *Fng* tKO mice express no *Fng* genes. Each symbol in the graphs represents the fold increase in N1 expression following activation of T cells from one mouse. Error bars reflect ± SD. tKO, triple knockout.

While CD4/CD8 double negative T cell progenitors from *Fringe* triple KO mice show reduced DLL4 binding and reduced expression of activated N1 target genes compared to *Fng* LMR controls (28), we could not obtain sufficient N1 from DN T cell progenitors to perform mass spectral analysis. To obtain cells with higher levels of endogenous N1, naïve T cells from spleen were activated by incubation with anti-CD3/CD28 *in vitro* as described previously (35). The expression of CD8 in activated T cells from Fringe control and mutant groups was similar (Fig. S4*A*). Although there was some variation between groups in the proportion of CD4-high *versus* CD4-low activated T cells in Fig. S4*A*; no significant differences were observed when numerous mice from each group were compared (Fig. S4*B*). Thus, activated T cells were analyzed for cell surface expression of N1 (Fig. 3). Activated T cells bound approximately 10 times more antibody to N1-ECD than naïve T cells. Western blot analysis of activated T cells showed similar amounts of N1 to preT 2017 cells, but the major form of N1 in activated T cells was the cleaved cell-surface form, whereas the major form in preT 2017 cells was the ER form (Fig. S4*C*).

### The *O*-fucose on N1 EGF16 is the major Fringe extension site in activated T cells

**Figure 4:**
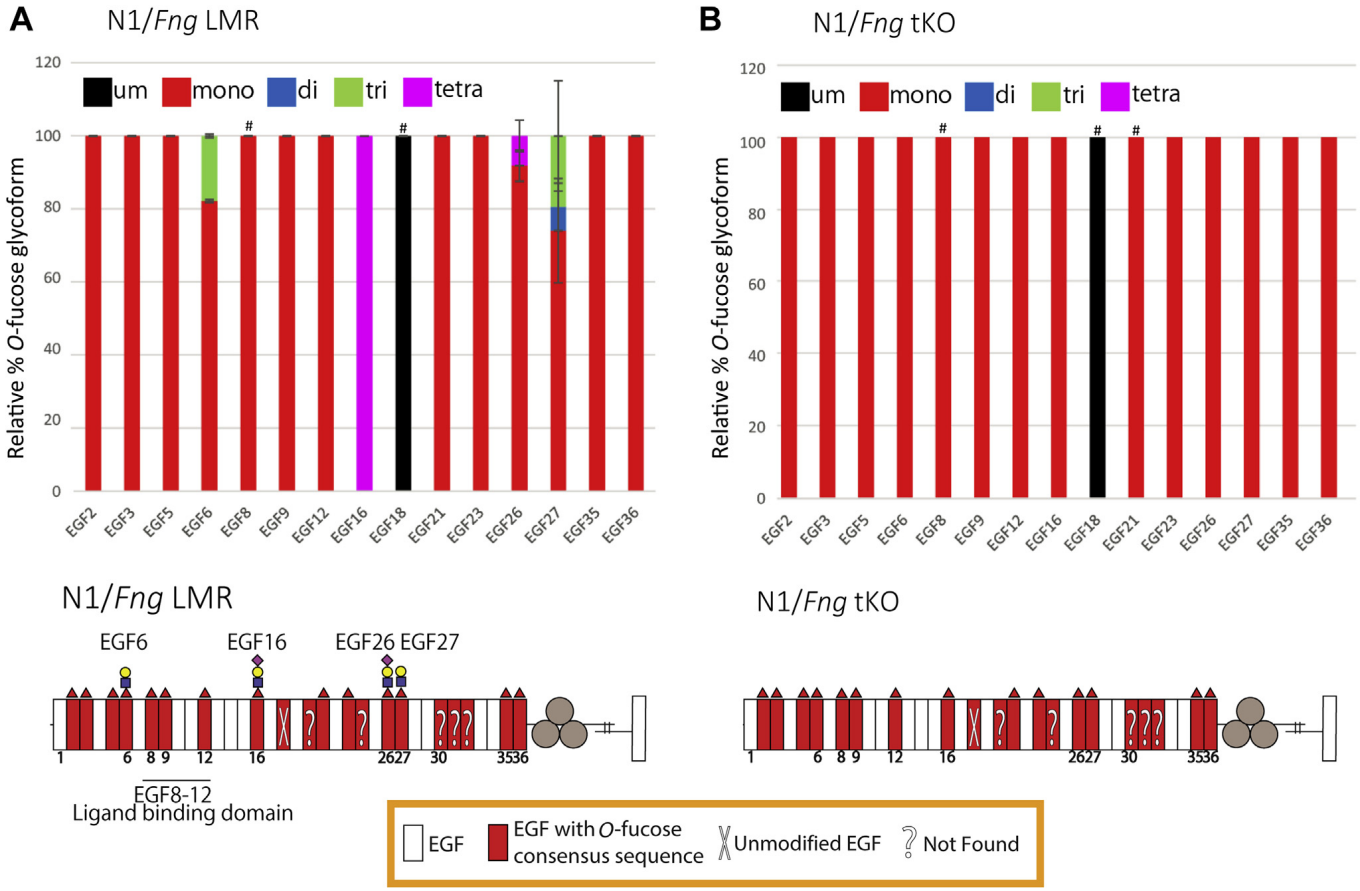
**Endogenous N1 from activated T cells is modified by *O*-fucose at high stoichiometry, but only EGF6, EGF16, EGF26, and EGF27 are modified by Fringes.***Top*: Bar graphs show the relative amount of *O*-fucose glycoforms on peptides from each N1 EGF repeat detected. *Black*, *red*, *blue*, *green*, and *magenta* indicate the unmodified (um), monosaccharide, disaccharide, trisaccharide, and tetrasaccharide glycoforms, respectively. Average of three biological replicates is shown. Error bars show SD. # indicates peptides that were only detected in two of the biological replicates. Mass spectral data can be found in Fig. S11 and Tables S3–S5. Representative EICs are shown in Fig. S5. *Bottom*: Summary of mass spectral analysis of endogenous N1 from *Fng* LMR (*A*) or *Fng* tKO (*B*) activated T cells. N1 ECD shown as in Fig. 2*A*. The most extended form of the *O*-fucose glycan detected is shown. ECD, extracellular domain; EGF, epidermal growth factor-like; EIC, extracted ion chromatogram.

Activated T cells expressed comparable levels of mRNA for *Pofut1*, *Poglut1*, *Lfng*, *Mfng*, and *Rfng* (Fig. S3). To examine the Fringe-mediated elongation of *O*-fucose on endogenous N1, we purified N1 by immunoprecipitation from activated T cells derived from *Fng* LMR (Fig. S2*B*) or *Fng* tKO mice and performed mass spectral analysis as described previously. Peptides containing *O*-fucose sites were detected for 15 of the 20 predicted sites (Tables S3–S5). Peptides for EGF20, EGF24, EGF30, EGF31, and EGF32 were not detected (Fig. 4). EICs were generated for each of the detected peptides (Fig. S5). All of the peptides containing *O*-fucose sites were modified by *O*-fucose at high stoichiometry except EGF18, which was unmodified (Figs. 4 and S5), consistent with the preT 2017 cell results (Fig. 2*A*). Extension of *O*-fucose was detected on N1 EGF6, EGF16, EGF26, and EGF27 from *Fng* LMR activated T cells, but the stoichiometry was partial (Figs. 4*A* and S5). Interestingly, only the *O-*fucose on EGF16 was fully extended to the *O*-fucose tetrasaccharide, EGF6, EGF26, and EGF27 being partially extended. Extension of *O*-fucose was not detected in N1 from *Fng* tKO activated T cells, confirming that the extension in N1 from *Fng* LMR T cells was due to Fringe activities (Figs. 4*B* and S5). These results were quantified and confirmed in biological triplicate analyses (Fig. 4).

**Figure 5:**
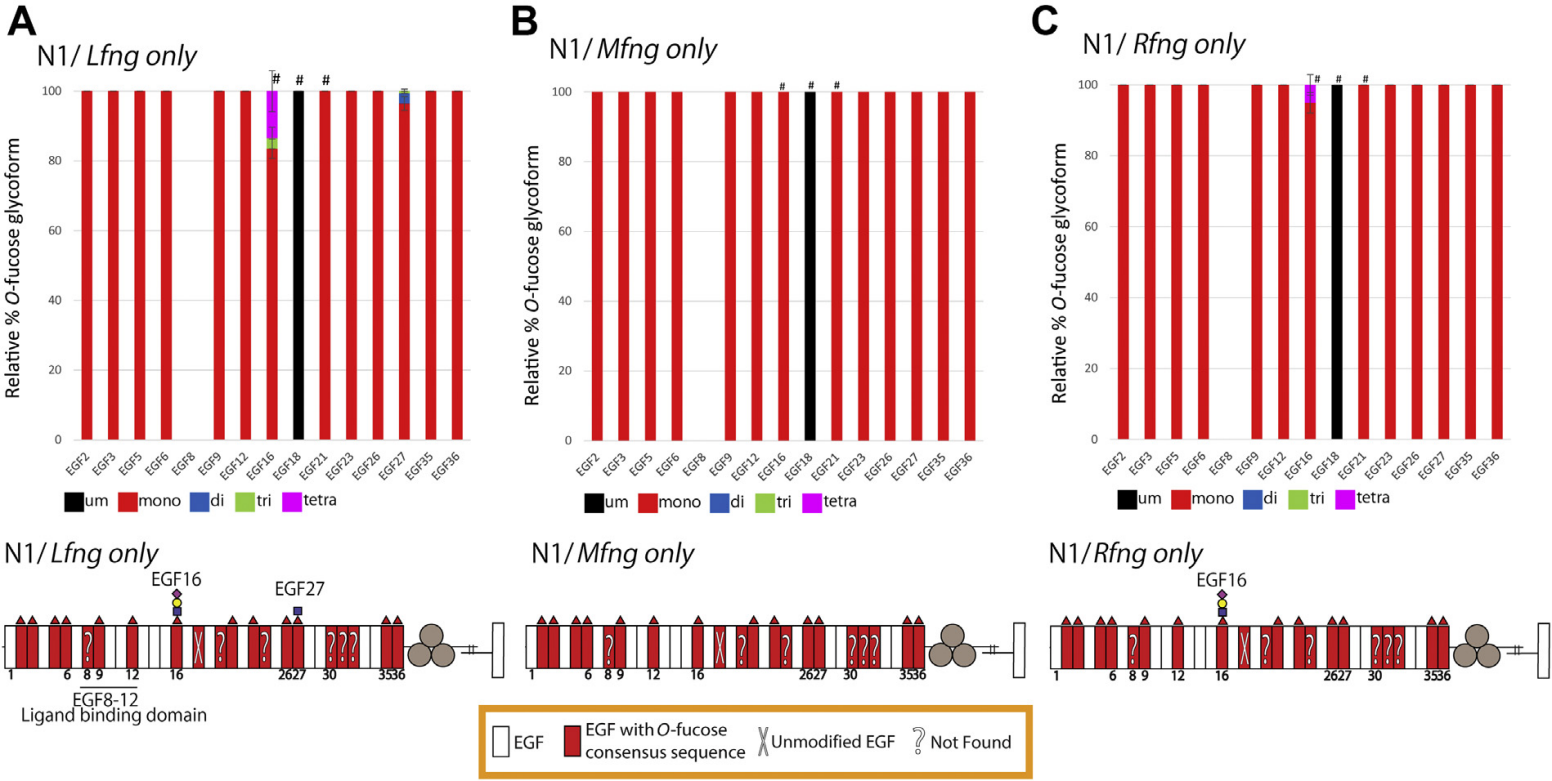
**N1 from activated T cells expressing a single Fringe had fewer *O*-fucose extensions.***Top*: Bar graphs show the relative amount of *O*-fucose glycoforms on peptides from each EGF repeat detected in N1 from activated T cells expressing only *Lfng* (*A*) or only *Mfng* (*B*) or only *Rfng* (*C*). *Black*, *red*, *blue*, *green*, and *magenta* indicate the unmodified (um), monosaccharide, disaccharide, trisaccharide, and tetrasaccharide peptide glycoforms, respectively. Average of three biological replicates is shown. Error bars show SD. # indicates peptides that were only detected in two of the biological replicates. Mass spectral data can be found in Fig. S12 and Tables S6–S8. Representative EICs are shown in Fig. S6. *Bottom*: Summary of mass spectral analysis of endogenous N1 from *Lfng-only* (*A*) or *Mng*-only (*B*) or *Rfng*-only (*C*) activated T cells. N1 ECD shown as in Fig. 2*A*. ECD, extracellular domain; EGF, epidermal growth factor-like; EIC, extracted ion chromatogram.

To determine the contribution of each Fringe, we analyzed extension of *O*-fucose on N1 from activated T cells derived from mice expressing a single *Fringe* gene (*i.e.*, *Rfng/Mfng* double KO [*Lfng* only], *Lfng/Rfng* double KO [*Mfng* only], and *Lfng/Mfng* double KO [*Rfng* only] mice). *Lfng*-only T cells had ∼20% extension of *O-*fucose at EGF16 and less than 5% extension of *O-*fucose at EGF27 (Figs. 5*A* and S6). *Mfng*-only T cells did not have any extension of *O-*fucose (Figs. 5*B* and S6), and *Rfng*-only T cells had ∼10% extension of *O-*fucose at EGF16 (Figs. 5*C* and S6). These results suggest that LFNG makes a larger contribution to the modification of N1 than either MFNG or RFNG in activated T cells. In addition and importantly, the data indicate that all three Fringes are necessary to obtain the modification level observed in N1 from activated T cells of *Fng* LMR mice.

### No extension of *O*-fucose on EGF12 by Fringe in activated T cells

**Figure 6:**
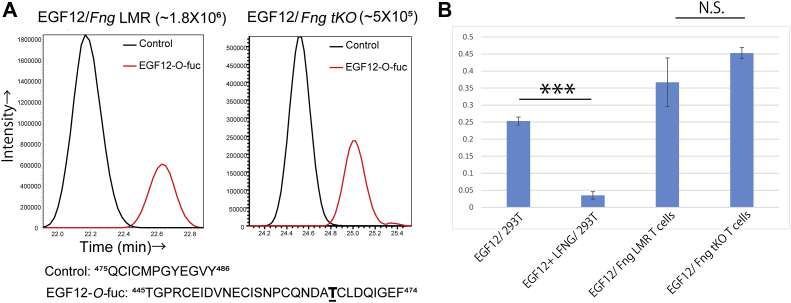
**EGF12 is not modified by Fringe in *Fng* tKO activated T cells.***A*, EICs show relative levels of mN1 control peptide lacking an *O-*fucose site from EGF12 (*black line*: ^475^QCICMPGYEGVY^486^) *versus* mN1 EGF12 peptide with monosaccharide *O*-fucose modification (*red line*: ^445^TGPRCEIDVNECISNPCQNDA**T**CLDQIGEF^474^, *O*-fucose site bold underlined) from *Fng* LMR or *Fng* tKO activated T cells. *B*, ratio of EGF12 peptide to control peptide from mN1 expressed in HEK293T cells in the absence or presence of LFNG (shown in Fig. S9) or mN1 isolated from *Fng* LMR or *Fng* tKO activated T cells. NS ≥ 0.05, ∗*p* < 0.05, ∗∗*p* < 0.01, ∗∗∗*p* < 0.001. Mass spectral data for EGF12 and control peptide are shown in Table S4. Error bars reflect ± SD. EGF, epidermal growth factor-like; EIC, extracted ion chromatogram; tKO, triple knockout.

It was surprising that no Fringe extension of the *O*-fucose on EGF12 was detected in mN1 from activated T cells since this site is important *in vivo* (31, 32, 33) and for Fringe-dependent effects in cell-based assays (4, 13). It was possible that an unusual, Fringe-dependent glycan at EGF12 that was not detected in our Byonic search string was present. One such *O*-fucose glycan carrying glucuronic acid has been reported in *Drosophila* (41), although this glycan was not shown to be on Notch. To rule out the possibility of an unknown modification extending *O*-fucose on EGF12 of N1 in activated T cells, we quantified the level of *O*-fucose monosaccharide on the EGF12 peptide in *Fng* LMR and *Fng* tKO samples compared to a control peptide from N1 without any glycan modifications. We demonstrated that this method works by comparing the ratio of EGF12 *O*-fucose-modified peptide to control peptide lacking an *O-*fucose site (EGF12/control) using full-length N1 expressed in HEK293T cells, with or without *Lfng*. As expected, the presence of LFNG reduced the ratio of unextended *O*-fucose peptide significantly (Fig. S7), and the ratio was consistent over a large range of samples analyzed (Fig. S7). This is consistent with the extension of *O*-fucose on EGF12 by LFNG as shown in Fig. S1*B*. We repeated this analysis using the same peptide from EGF12 of N1 from *Fng* LMR and *Fng* tKO activated T cells (Fig. 6*A*). There was no significant difference between the *Fng* LMR and *Fng* tKO samples (Fig. 6*B*), consistent with the conclusion that N1 EGF12 does not have *O*-fucose extension in *Fng* LMR activated T cells.

### Independent loss of *O*-fucose on N1 EGF16 or EGF27 had an effect on binding to DLL1 or DLL4 in the presence of LFNG

**Figure 7:**
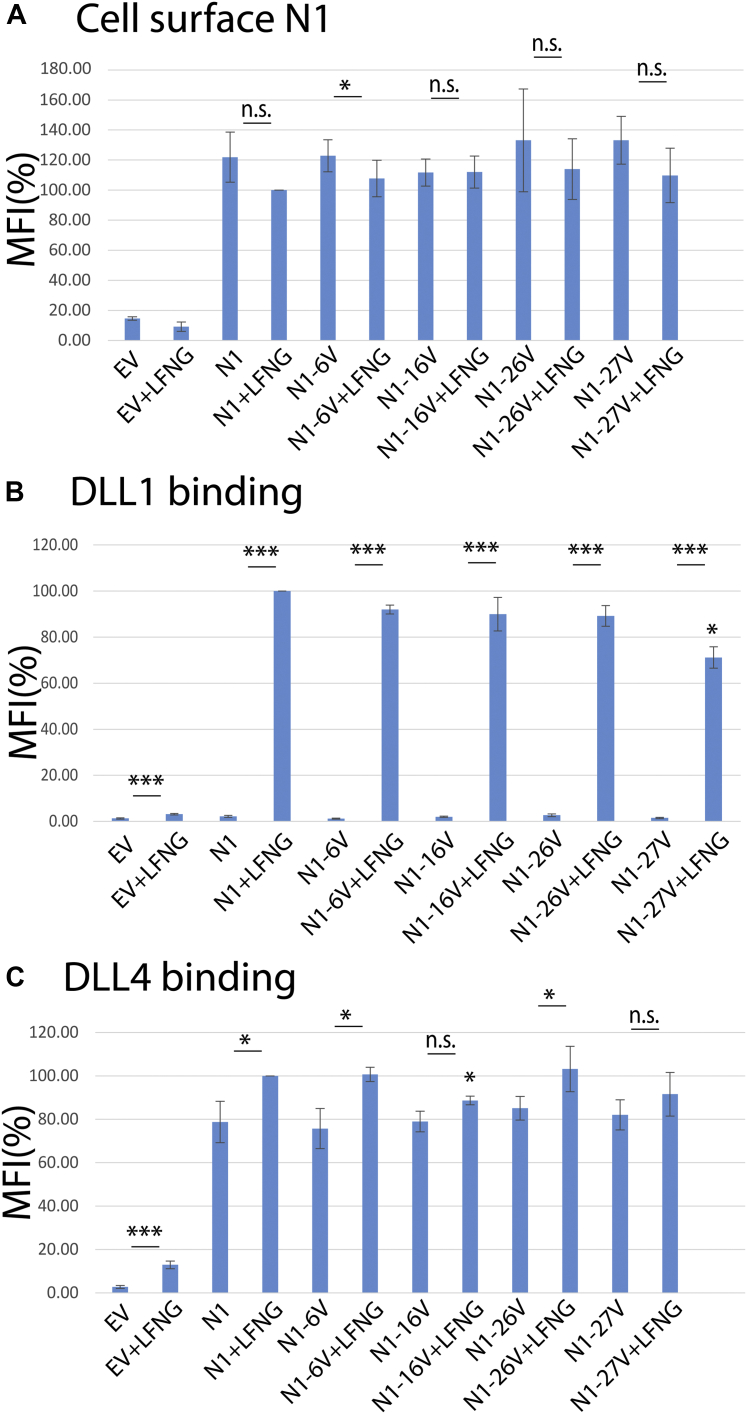
**Elimination of *O*-fucose sites in EGF6, EGF16, EGF26, or EGF27 has small effects on the binding of DLL1 or DLL4 to N1.** WT N1, N1-EGF6V, N1-EGF16V, N1-EGF26V, or N1-EGF27V with or without *Lfng* cDNAs were overexpressed in HEK293T cells as described in Experimental procedures. Mean fluorescent intensity (MFI) of cell surface N1 (*A*), DLL1-Fc binding (*B*), and DLL4-Fc binding (*C*) is shown. Average of three biological replicates is shown. Error bars show SD. The lines with ∗ above are the comparison of the samples with or without LFNG. The ∗ above the N1-27V+LFNG in (*B*) and N1-16V+LFNG in (*C*) are comparison to WT+LFNG. NS ≥ 0.05, ∗*p* < 0.05, ∗∗*p* < 0.01, ∗∗∗*p* < 0.001. cDNA, complementary DNA; DLL1, Delta-like ligand; EGF, epidermal growth factor-like.

None of the Fringe extension sites in N1 from activated T cells (EGF6, EGF16, EGF26, and EGF27) are within the N1 ligand-binding domain (EGF8–12) (4, 29, 30). To test whether eliminating the Thr *O*-fucose site in N1 EGF6, EGF16, EGF26, or EGF27 by mutating T to V might have an effect on DLL1 or DLL4 binding to N1, we performed cell-based binding assays using N1 overexpressed, with or without *Lfng*, in HEK293T cells as surrogates for activated T cells. Each mutation expressed alone in the context of full-length N1 had no effect on cell surface levels of N1 overexpressed in HEK293T cells (Fig. 7*A*). As we and others have shown before (11, 42), DLL4 binds N1 much better than DLL1 in the absence of LFNG (Fig. 7, *B* and *C*). DLL1 binding was strongly enhanced by the presence of LFNG, while DLL4 binding was only slightly increased. The T to V mutation in EGF16 and EGF27 had a small but significant effect on the ability of LFNG to enhance binding to DLL4 or DLL1, respectively (Fig. 7, *B* and *C*, respectively). Thus, Fringe-enhanced Notch ligand binding would be reduced in *Fng* tKO activated T cells, consistent with previously reported reduced binding of DLL4 to *Fng* tKO naïve T cells (28). To further examine the importance of the *O*-fucose on EGF16, we performed a cell-based N1 signaling assay (Fig. S8). Elimination of the *O*-fucose site on EGF16 in N1 overexpressed in Chinese hamster ovary (CHO) cells had no apparent effect on N1 activation by DLL1, DLL4, or JAG1, with or without coexpression of *Lfng.* However, this assay only partially mimics the situation in *Fng* tKO activated T cells that have *O*-fucose on EGF16 of N1 (Fig. 4*B*).

## Discussion

Here, we show that POFUT1 consensus sites in endogenous N1 isolated from mouse preT 2017 or activated T cells are modified at high stoichiometry with *O*-fucose, consistent with prior analysis of N1 ECD overexpressed in HEK293T, CHO, or U2OS cells (4, 13). In *Fng* LMR activated T cells, *O-*fucose was also found at high stoichiometry. However, Fringe extension of *O*-fucose was detected on only four fucosylated EGF repeats, EGF6, EGF16, EGF26, and EGF27, with the highest degree of modification on EGF16. This represents a subset of Fringe-modified sites on overexpressed N1 ECD expressed in HEK293T, CHO, or U2OS cells with cotransfected Fringe complementary DNAs (cDNAs) (4, 13). All Fringe-mediated extension of *O-*fucose was absent in N1 from *Fng* tKO activated T cells, consistent with expectation in the absence of all three Fringes. Most interesting was that N1 from activated T cells expressing only a single Fringe showed only small amounts of elongation on EGF16 and EGF27 (LFNG only), small amount of elongation of EGF16 (RFNG only), or no detectable elongation (MFNG only). These results suggest that LFNG is more active than either MFNG or RFNG, consistent with previous results (4, 13, 43). More importantly, the data indicate that all three Fringes work together to extend more *O*-fucose sites. Mutagenesis of individual *O*-fucose sites in EGF6, EGF16, EGF26, or EGF27 caused a small but significant reduction of DLL1 binding in the N1–EGF27V mutant expressed with LFNG and reduced DLL4 binding to N1–EGF16V in the presence of LFNG. These results are consistent with reduced DLL4 binding to naïve T cells from *Fng* tKO mice (28). A caveat of the mutagenesis experiments is that the effects are a combination of removing the *O*-fucose and any Fringe-mediated extension rather than effects of removing Fringe-mediated extension alone. To determine if the absence of Fringe activities alter functionalities of activated T cells, extensive *in vivo* studies would be required in the relevant Fringe mutant mice.

The high stoichiometry of *O-*fucose modification of N1 EGF repeats shows that POFUT1 is a highly efficient enzyme in preT 2017 and activated T cells. Similarly, high levels of *O*-fucosylation were detected on endogenous Notch isolated from *Drosophila* embryos (44). Prior studies showed that POFUT1 only modifies properly folded EGF repeats containing the POFUT1 consensus sequence (45), and thus, the EGF repeats of endogenous mouse N1 are properly folded. Since POFUT1 is localized to the ER, it has been implicated as a folding sensor of individual EGF repeats in N1 (39, 46). POGLUT1 is also localized to the ER, only modifies properly folded EGF repeats (47, 48, 49) and has been implicated as a folding sensor for EGF repeats (39, 48). Loss of POFUT1 or POGLUT1 has been shown to reduce cell surface expression of N1 in HEK293T cells (39), but this has not been observed in other cells (50, 51), suggesting that effects on N1 trafficking are cell-type specific. The fact that *Poglut1* mRNA levels were very low in preT 2017 cells is consistent with low levels of *O*-glucose on EGF12 of endogenous N1 and may explain why N1 was not expressed on the surface of these cells in contrast to N1 in activated T cells. In contrast, *Poglut1* mRNA levels in activated T cells were comparable to *Pofut1*, and *O*-glucose modification of EGF12 was high, consistent with the predominantly cell surface localization of N1 in these cells. There are several EGF repeats in N1 that contain POGLUT1 modification sites, but do not have POFUT1 modification sites (EGFs 4, 10, 13, 14, 17, 19, 25, 28, and 33 (52, 53)), that may require *O*-glucosylation for efficient folding and trafficking of N1 to the cell surface in preT 2017 cells (39).

The sites modified by Fringes in the activated T cells are a subset of those modified by LFNG when overexpressed with N1 ECD in HEK293T, CHO, or U2OS cells (4, 13). It was surprising that we saw significant Fringe modification of EGF16 but no modification of other sites, especially EGF8 or EGF12 in the N1 ligand-binding domain, even though transcripts for all three Fringe genes were present at similar levels to those of *Pofut1* in the activated T cells (Fig. S3). The simplest explanation for this is that *O*-fucose residues on some EGF repeats are better substrates for Fringes than others. We previously provided data supporting this idea using *in vitro* assays of several *O*-fucosylated EGF repeats (43). Based on our results, we would predict that the *O*-fucose on EGF16 is a much better substrate than other EGF repeats for Fringe modification. We have also shown that the ratio of Fringes to N1 expressed in a cell plays an important role in determining the extent of modulation of N1 activity (4, 11), so the level of expression of N1 in activated T cells could affect the level of Fringe modification. In addition, the presence of Fringe transcripts does not necessarily correspond to Fringe modification of *O*-fucose in cells (13). Similarly, lack of correspondence between mRNA levels of glycosyltransferases and the glycans expressed in the same cells has been reported (54).

Although we have shown that LFNG or MFNG modification of *O*-fucose on EGF6 prevents Fringe-mediated inhibition of JAG1–N1 signaling in cell-based assays (4), we do not have a clear idea of how Fringe modifications at EGF16, EGF26, or EGF27 affect N1 activity. EGF26 and EGF27 are in the *Abruptex* region of N1, and mutations in this region of *Drosophila* Notch cause hyperactivity and resistance to the effects of ectopic *fringe* expression (55). We previously showed that mutagenesis of the *O*-fucose sites on EGF26 or EGF27 have small effects on DLL1–N1 or JAG1–N1 activation in cell-based signaling assays but that Fringes still modulated N1 activity similar to WT N1 (4). Here, we showed that the EGF26 and EGF27 *O-*fucose site mutants still bind DLL1 and JAG1 in cell-based assays and binding to both is enhanced by LFNG as shown previously (4). However, there is a statistically significant decrease in the ability of LFNG to enhance DLL1–N1 binding of the EGF27 mutant in HEK293T cells (Fig. 7*B*). We had not previously analyzed the effects of eliminating the *O*-fucose on EGF16 in prior studies, but here, we showed that it had no effect on the ability of LFNG to enhance DLL1–N1–EGF16V binding, although it does cause a statistically significant decrease in LFNG’s ability to enhance DLL4–N1–EGF16V binding in HEK293T cells (Fig. 7). This is the first report of a biological effect of Fringe extension of *O*-fucose on EGF16. No differences were detected in ligand-induced activation assays of CHO cells overexpressing N1–EGF16V in the presence or absence of LFNG and stimulated by DLL1, DLL4, or JAG1 (Fig. S8). Of course, the Fringe modifications on EGF6, EGF16, EGF26, and EGF27 in activated T cells could have effects on N1 that are not measured in our cell-based ligand-binding or activation assays in HEK293T or CHO cells, respectively.

The high degree of *O*-fucose modifications of endogenous N1 isolated from preT 2017 or activated T cells is very similar to that of N1 EGF1-36 or EGF1-18 expressed in HEK293T, CHO, or U2OS cells (4, 13), except for EGF18 and EGF23. In two prior studies, EGF18 was shown to be modified by *O*-fucose, but in preT 2017 and activated T cells the EGF18 peptide was completely unmodified. In contrast, EGF23 was previously shown to be unmodified, but in this study, it was modified. This may be due to a difference in the ionization efficiency of the *O*-fucosylated peptides from EGF18 or EGF23 detected in this study compared to those identified in the prior studies. Alternatively, a soluble, secreted fragment of the N1 ECD lacking the negative regulatory region (Fig. 2*A*) used in prior studies may be modified differently than the full-length, endogenous N1 characterized in this study. A recent analysis of N1 structure using crosslinking mass spectral analysis suggested that N1 is folded such that parts of the ligand-binding domain (EGF8–12) interact with the negative regulatory region (56). Lack of *O*-fucosylation on EGF18 or the presence of *O*-fucose on EGF23 on endogenous N1 could result from changes in access of the sites caused by this folding, altering *O*-fucose modification of the consensus sequence in certain EGF repeats of the membrane-bound, folded structure.

## Experimental procedures

### Plasmids

Mammalian expression plasmids encoding mouse LFNG (pAPtag2-LFNG), full-length mouse N1 (pcDNA1-N1), and *O*-fucose site mutants of mouse N1 (pcDNA1-N1-EGF6V, pcDNA1-N1-EGF26V, pcDNA1-N1-EGF27V) were described previously (4). The RFP plasmid was obtained from Addgene (#12520), the TP1-1 luciferase reporter construct was kind gift from Dr Georg Bornkamm, and the gWIZ β-galactosidase construct was from Gene Therapy Systems. The *O*-fucose site mutant in N1 EGF16 (pcDNA1-N1-EGF16V) was generated by PCR-directed mutagenesis with primers (Table S9) and CloneAmp HiFi (TAKARA). All mutants were confirmed by sequencing.

### Cell culture

HEK293T cells (from ATCC) were cultured in Dulbecco's Modified Eagle’s Medium (DMEM) of high glucose media with 10% bovine calf serum (BCS) at 37 °C in a humidified incubator at 5% CO_2_. CHO Pro-5 cells (from ATCC) were cultured in minimum essential medium (MEM) α without nucleosides with 10% BCS at 37 °C in a humidified incubator at 5% CO_2_. Murine PreT 2017 cells were kind gift from Dr Isabella Screpanti (37) and were cultured in α-MEM with 10% fetal bovine serum (FBS) at 37 °C in a humidified chamber at 5% CO_2_.

### Mice

Mice heterozygous for *Lfng* and with inactivating mutations in *Mfng* and *Rfng* on a mixed C57BL/6/FVB background were a gift of Dr Susan Cole (University of Ohio). Their origin was previously described (19). tKO (*Fng* tKO) mice were obtained by intercrossing, and mice expressing one copy of all *Fng* genes (*Fng* LMR) were obtained by crossing to FVB as previously described. Further intercrossing generated mice that expressed a single *Fng* gene—*Lfng* or *Mfng* or *Rfng* (28). Genomic DNA was used to genotype progeny as previously described (19, 28). Mice housed in a barrier facility at Albert Einstein College of Medicine were permitted to eat and drink *ad libitum.* Spleens were isolated at 6 to 8 weeks of age. The Albert Einstein Institutional Use and Animal Care Committee reviewed and approved experimental protocols (numbers 20140803 and 20170709).

### T-cell activation

To obtain splenocytes, spleen from control (*Fng* LMR) or *Fng* mutant mice was removed and placed in 1 ml MACS buffer (1× PBS [Ca^++^ Mg^++^ free], pH 7.2, containing 0.5% bovine serum albumin fraction V [Sigma]). Each spleen was disrupted with the head of a 1 ml syringe plunger on a 70 μm cell strainer in a 50 ml falcon tube. The strainer was washed thrice with 5 ml MACS buffer. To remove red blood cells, splenocytes were pelleted (1200 rpm, 10 min at 4 °C), resuspended in 3 ml, freshly prepared red blood cell lysis buffer (0.15 M NH_4_Cl, 10 mM KHCO_3_, 0.1 mM EDTA, pH 7.2–7.4), and incubated on ice. After 3 min, 30 ml cold MACS buffer was added and transferred to a new 50 ml falcon tube through a 70 μm strainer. After centrifugation, the pellet was resuspended in 3 ml MACS buffer and cells were counted in a Coulter counter. A small aliquot of splenocytes (5 × 10^5^) were fixed in 4% paraformaldehyde (PFA) in PBS and stored at 4 °C for flow cytometry. T cells were isolated from ∼1.5 × 10^8^ splenocytes using the Pan T Cell Isolation Kit (Miltenyi Biotech; catalog no.: # 130-095-130) according to the manufacturer’s protocols. Briefly, splenocytes were centrifuged, resuspended in 600 μl MACS buffer and 150 μl biotinylated antibodies cocktail was added. After incubation for 10 min at 4 °C, 450 μl MACS buffer and 300 μl antibiotin microbeads were added, and incubated at 4 °C for 20 min with gentle shaking. The LS column was placed in MACS separator and rinsed with 3 ml MACS buffer. Thereafter, the cell suspension was passed through the column and enriched T cells were collected as flow-through in a 15 ml round-bottom collecting tube. The LS column was washed twice with 5 ml MACS buffer. Enriched T cells were counted in a Coulter counter and 5 × 10^5^ enriched T cells were fixed in 4% PFA in PBS and stored at 4 °C for later use in flow cytometry. The remaining cells were used for T-cell activation.

A day before T-cell activation, a 10 cm tissue culture dish was coated with 5 μg/ml of anti-CD3 (eBioscience; catalog no.: # 16-0032-82) and 5 μg/ml of anti-CD28 antibodies (eBioscience; catalog no.: # 16-0281-82) in PBS at 4 °C. The next day, PBS was removed and enriched T cells (1–2.5 × 10^7^) were added in complete medium (RPMI containing 10% FBS, penicillin/streptomycin, beta-mercaptoethanol (50–55 μM) (Gibco; catalog no.: #21985-023) and 5 ng/ml and IL-2 (PeproTech: catalog no.: #212-12) and cultured at 37 °C in a CO_2_ incubator. After 48 h, the activated T cells were removed by gently pipetting the medium and counted in a Coulter counter. A small aliquot (∼5 × 10^5^) of cells was fixed in 4% PFA in PBS and stored at 4 °C for flow cytometry. The remainder was washed with 1 ml PBS containing 1 mM CaCl_2_, and the pellet was stored at −80 °C.

### Flow cytometry for T cells

Total splenocytes, CD4+, and CD8+ enriched T cells and activated T cells stored at 4 °C after fixation in PFA were washed once with 1 ml ligand-binding buffer (LBB) (Hank’s balanced salt solution containing 1% bovine serum albumin, 1 mM CaCl_2_, and 0.05% sodium azide) and incubated with 1 μl purified rat–antimouse CD16/CD32 (BD Biosciences; catalog no.: #553141; Clone 2.4G2) in 90 μl LBB. Following incubation on ice for 15 min, 10 μl containing a mix of anti-CD4-FITC (eBioscience, catalog no.: #11-0042, Clone;RM4-5, 1:200), anti-CD8-APC (eBioscience; catalog no.: #17-0081, Clone:53-6.7, 1:200), and anti-N1 Ab (R&D Systems; catalog no.: #AF5267, 1:50) was added. After incubation for 30 min at 4 °C in the dark, 1 ml LBB was added, cells were pelleted, and washed once with 1 ml LBB. Rhodamine Red X-conjugated, antisheep IgG (Jackson ImmunoResearch, catalog no.: #713-295-147 1:100) antibody was added and incubated for 30 min at 4 °C in the dark. The cells were washed twice with 1 ml LBB, and N1 cell surface expression was determined using a FACS Calibur (BS Biosciences) flow cytometer. Data were analyzed using FlowJo Software (FlowJo, LLC).

### Overexpression of mN1 in HEK293T cells

Transfection methods were described previously (4). Briefly, HEK293T cells cultured in 10 cm dishes were transfected with 3 μg pcDNA1-mouse N1 or mouse N1 point mutants and 1.5 μg of pAPtag2-LFNG or empty vector using polyethyleneimine (PEI) in DMEM. Media was changed to fresh DMEM after 6 to 8 h of transfection. Cells were generally used for assay after 48 h of transfection.

### mN1 immunopurification

PreT 2017 cells (∼2.5 × 10^7^ cells), HEK293T cells transfected with mN1 with or without LFNG as described previously (1 × 10^7^ cells), or activated T cells from spleen (∼2.5–3.0 × 10^7^ cells) were lysed in 1 ml Tris-buffered saline (TBS, 10 mM Tris-HCl, pH 7.5, 0.15 M NaCl) with 1% NP-40 and cOmplete protease inhibitor without EDTA (Sigma) and incubated on ice for 20 min. Cell debris was removed by centrifugation (12,000 rpm for 5 min at 4 °C), and the supernatants were used for immunoprecipitation. A portion was saved for Western blots. Sheep anti-mN1 antibody (15 μg, AF5267; R&D Systems) was covalently coupled to 60 μl of protein G Dynabeads (catalog no.: #10003D, Thermo) using BS3 (catalog no.: #21580, Thermo) as described by the manufacturer. The antibody-bound Dynabeads were washed three times with TBS, 1% NP-40 by collecting with a magnet, then then times with TBS alone, and the remaining cell supernatant was added. The beads and cell lysate were incubated at 4 °C for 8 to 12 h with tilting rotation. The beads were collected with a magnet and washed three times with TBS, 1% NP-40, then three more times with TBS alone. Protein was eluted by adding 15 μl of 8 M urea in water for 10 min at 37 °C. Aliquots of each fraction were analyzed by Western blot using the sheep antimouse N1 antibody to confirm the efficiency of the immunoprecipitation.

### Glycoproteomic mass spectral analysis of mN1

Reduction, alkylation, digestion, and mass spectral analysis are based on our previous protocol (4). Tris(2-carboxyethyl)phosphine (25 mM final, Thermo) was added to the immunopurified N1 in 8 M urea and heated to 100 °C for 5 min. After cooling to room temperature, iodoacetamide (25 mM final concentration) was added and incubated in the dark for 30 min. The sample was diluted eightfold using mass spectral grade water. Diammonium phosphate (20 mM final concentration) was added, followed by 0.5 μg of trypsin (Sigma), 0.5 μg of chymotrypsin (Thermo), or 0.5 μg of V8 (Thermo). Digestion was performed for 4 to 6 h at 37 °C. Peptides were desalted using Pierce C18 Spin Tip (Thermo), washed with 0.1% formic acid, and eluted with 50% acetonitrile in 0.1% formic acid. Peptides were separated using an Easy nano-LC HPLC system with a C18 EasySpray PepMap RSLC C18 column (50 mm 3 15 cm, Thermo). Separation of glycopeptides was carried out using a 30 min binary gradient consisting of solvent A (0.1% formic acid in water) and solvent B (90% acetonitrile and 0.1% formic acid in water) with a constant flow rate of 300 nl/min. Peptides were detected by a Q Exactive Plus mass spectrometer (Thermo Fisher Scientific). Higher energy collisional dissociation-tandem MS method was used, and the 10 most abundant precursor ions in each MS scan were selected for fragmentation (collision energy was 27%, 2 × 10^5^ gain control, isolation window m/z 3.0, dynamic exclusion enabled, and 17,500 fragment resolution). Peak lists and raw data files were generated using Xcalibur software (Thermo) set to its default settings. Raw data files were analyzed using Proteome Discoverer 2.1.0.81 (Thermo) and were searched against a mN1 ECD database (Q01705, 18 April 2012—v3). Byonic software version 2.10.5 (Protein Metrics) was used as a node inside Proteome Discoverer for identifying peptides with glycan modifications. Two missed cleavages were allowed. Fixed modification was carbamidomethyl (+57.021464) on cysteines; variable modifications were oxidation (+15.994915) on methionine, histidine, asparagine, and aspartic acid. Glycoforms were searched as rare 1 modification: fucose (+146.057909), fucose-HexNAc (+349.137281), fucose-HexNAc-hexose (+511.190105), Fucose-HexNAc-Hexose-NeuAc (+802.285522), Fucose-HexNAc-Hexose-NeuGc (+818.280436), Hexose (+162.052824), Hexose-Pentose (+294.095082), Hexose-Pentose-Pentose (+426.137341), HexNAc (+203.079373), HexNAc-Hexose (+365.132196), and HexNAc-Hexose-NeuAc (+655.227613). Protein and peptide false discovery rates were set to a threshold of 1% and calculated in Byonic software version 2.10.5 using the two-dimensional target decoy strategy as described (5). EICs for parent ions of all glycopeptides were generated using Xcalibur Qual Browser 4.0.27.19 (Thermo) with precursor mass tolerance set to 20 ppm. The glycoform distribution on each EGF repeat was quantified based on area under the curve of each EIC for all biological and technical replicates. Byonic search results are provided in Tables S1–S8. Parallel reaction monitoring method was used for quantitative analysis of EGF12 peptide modified with *O*-fucose monosaccharide. The m/z 1028.48 ion was followed since it is the most abundant and reproducible ion in the MS/MS spectra (Fig. S10*A*). The m/z 693.24 ion was followed for the parallel reaction monitoring of the control peptide (MS2 spectrum is in Fig. S11V). Raw data of mass spectral analysis results are uploaded to PRIDE (https://www.ebi.ac.uk/pride/), with project number PXD031297. A summary of the uploaded data is in Table S10.

### Notch ligand-binding assays and cell surface N1 analysis

HEK293T cells were transfected as described previously using 3 μg of pcDNA1-N1 or N1 with a T to V mutation in a single EGF repeat, 1.5 μg of pAPtag2-LFNG or empty vector, and 1 μg of RFP plasmid. After 48 h, cells were fixed with 2% PFA and used for Notch ligand-binding assays or analysis of cell surface N1. After washing 1 × 10^5^ cells with binding buffer (Hank’s balanced salt solution containing 1% BCS, 0.05% azide, and 1 mM CaCl_2_), the cells were incubated with 50 nM DLL1-Fc (R&D Systems) or DLL4-Fc (R&D Systems) and anti-Fc phycoerythrin (PE) conjugated antibody (Jackson, 1:25 dilution) in binding buffer on ice for 1 h and washed in binding buffer. Binding was determined and analyzed using an Accuri C6 flow cytometer. Three thousand cells were gated for RFP expression, and PE intensity of the RFP-expressing cells was determined.

For cell surface mN1 detection, washed cells were incubated with sheep anti-mN1 antibody (AF5267; R&D Systems, 1:1000 dilution) or sheep nonspecific IgG (R&D Systems, 1:1000 dilution) on ice for 1 h. N1 antibody or nonspecific IgG were detected by antisheep Fc PE conjugate antibody (Thermo, 1/25). Binding was determined and analyzed using an Accuri C6 flow cytometer. Ten thousand cells were gated for RFP expression of HEK293T cells or preT 2017 cells, and PE intensity of gated cells was determined. Graphics were generated using BD Accuri C6 software (BD Biosciences) and Excel.

### Cell-based Coculture N1 activation assay

OP9 cells stably expressing DLL4 are kind gift from Dr Juan Carlos Zúñiga-Pflücker (57) and L cells stably expressing JAG1 or DLL1 were a kind gift of Dr Gerry Weinmaster (UCLA). N1 signaling assay was performed as previously described (11, 13). CHO cells (0.5 × 10^5^ cells/well) were seeded in each well and cultured for 48 h. Media were removed after 48 h, washed cells by PBS for one time, and α-MEM without serum was added. The cells were then coransfected with 0.2 μg of WT or mutant pcDNA1-N1, 0.1 μg of pAPtag2-LFNG or empty vector, 0.2 μg of TP1-1 of luciferase reporter, and 0.1 μg of gWIZ β-galactosidase of linear plasmid. PEI was used for the transfection. Media were removed after 4 h of transfection, washed once by PBS, and α-MEM with serum was added. L-cells cells stably expressing JAG1 and DLL1 or OP9 cells stably expressing DLL4 were added to the transfected cells at a density of 1.5 × 10^5^ cells/well for 24 h. Cells were lysed, and luciferase assays were performed based on the manufacturer’s instructions by Luciferase Assay System (Promega) as described previously (11, 13).

### mRNA expression in preT 2017 and activated T cells

PreT 2017 or activated T cells were harvested and frozen at −80 °C until use. Total RNA from 1 × 10^7^ cells was isolated and used for cDNA synthesis. cDNA from activated T or preT 2017 cells for technical or biological replicates was carried out, respectively, as described previously (58). The quantitative RT-PCR reactions were performed for each gene three times using the primers listed in Table S9. Amplification conditions and data analysis were performed as described previously (58). Briefly, Ct values for each gene were normalized by Ct values for *Gapdh*, and calculation of relative transcript abundance is performed.

## Statistical analysis

All experiments were performed in biological triplicates or more, and results were reported as the means ± SD. Statistical significance was determined using one-way ANOVA.

## Data availability

The mass spectrometry proteomics data have been deposited to the ProteomeXchange Consortium *via* the PRIDE (59) partner repository with the dataset identifier PXD031297.

## Supporting information

This article contains supporting information.

## Conflict of interest

The authors declare that they have no conflicts of interest with the contents of this article.
