# Supplementary material for: Fringe GlcNAc-transferases differentially extend O-fucose on endogenous NOTCH1 in mouse activated T cells
Source: J Biol Chem. 2022 May 25;298(7):102064. doi: 10.1016/j.jbc.2022.102064 (PMC9234238; doi:10.1016/j.jbc.2022.102064)

Supporting Information for:

O-Fucose glycans on endogenous mouse NOTCH1 from activated T cells of Fringe mutant mice

Kenjiroo Matsumoto, Vivek Kumar, Shweta Varshney, Alison V. Nairn, Atsuko Ito, Florian Pennarubia, Kelley W. Moremen, Pamela Stanley, Robert S. Haltiwanger

Contents:

Supporting Information Tables S1-S10
Supporting Information Figures S1-S12

Supporting Information Tables:

Table S1: Chymotryptic peptides from mouse N1 overexpressed with or without LFNG in HEK293T cells

Table S2: Tryptic, chymotryptic, or V8 peptides from endogenous N1 of preT 2017 cells

Table S3: Tryptic peptides from endogenous N1 of *Fng* LMR and *Fng* tKO activated T cells

Table S4: Chymotryptic peptides from endogenous N1 of *Fng* LMR and *Fng* tKO activated T cells

Table S5: V8 peptides from endogenous N1 of *Fng* LMR and *Fng* tKO activated T cells

Table S6: Tryptic peptides from endogenous N1 single *Fng* activated T cells

Table S7: Chymotryptic peptides from endogenous N1 of single *Fng* activated T cells

Table S8: V8 peptides from endogenous N1 of single *Fng* activated T cells

Table S9: Primer list for the qRT-PCR analysis, genotyping, and PCR mutagenesis

Table S10: Description of data uploaded to the PRIDE database

Supporting Information Figures:

Figure S1: Anti-N1 ECD antibody can immunoprecipitate LFNG-modified mouse N1

Full-length mouse N1 was expressed in HEK293T cells with or without LFNG and purified from cell lysates by immunoprecipitation with anti-mouse N1 ECD antibody. **A.** Western blot with anti-N1 ECD of immunoprecipitate elution shows that similar amounts of N1 were obtained from lysates expressing mouse N1 in the presence or absence of LFNG. Molecular weight markers are shown on the left in kDa. **B.** EIC showing glycoforms of EGF12 peptide (TGPRCEIDVNECISNPCQNDA**T**CLDQIGEF) from N1 expressed in 293T cells in the presence or absence of LFNG. Black, red, blue, green, and magenta lines show the unmodified, monosaccharide, disaccharide, trisaccharide, and tetrasaccharide glycoforms. Peptides also modified with *O*-glucose trisaccharide are indicated with an asterisk. Mass spectral data can be found in Table S1. Representative MS2 spectra are shown in Fig. S9.

Figure S2: Endogenous mN1 is efficiently immunoprecipitated from preT 2017 cells and activated T cell lysates

Endogenous N1 was immunopurified from cell lysates of murine preT 2017 cells (**A**) and activated T cells (**B**) as described in Experimental Procedures. Western blots with anti-N1 ECD of before immunoprecipitation, after immunoprecipitation, wash, and elution are shown. Molecular weight markers are shown on the left in kDa. Migration positions of full-length N1 (N1 FL), and N1 ECD (NECD) are shown.

Figure S3: qRT-PCR analysis of glycosyltransferases involved in modifying EGF repeats in preT 2017 and wild-type activated T cells

mRNA was purified from preT 2017 and activated T cells and analyzed by qRT-PCR, normalized by GAPDH as described in Experimental Procedures. Primers used for each enzyme are in Table S9. Average of three biological replicates is shown. Error bars show +/- SD. NS≥0.05, *p<0.05, **p<0.01, ***p<0.001.

Figure S4: Activated T cells from splenocytes, expression of CD4 in activated T cells, and mN1 in preT 2017 versus activated T cells.

A. T cells from mouse spleen were enriched using the Pan T Cell Isolation Kit. Fixed splenocytes or enriched T cells or activated T cells were incubated with anti-CD4 and anti-CD8 antibodies and analyzed by flow cytometry to determine the efficiency of enrichment. Representative flow cytometry analyses are shown of total splenocytes (upper panel), enriched T cells (middle panel) and activated T cells (lower panel) from control (*Fng* LMR) and Fringe mutant mice. **B.** Representative flow cytometric dot plot of activated T cells from *Fng* LMR mice used to determine MFI of CD4-high versus CD4-low activated T cells (Upper panel). Histogram showing the MFI for CD4 in CD4-high and CD4-low activated T cells (Lower panel). *Fng* LMR mice express one allele of each *Fng* gene; *Lfng, Mfng* or *Rfng* mice express one allele of the single *Fng* gene noted; *Fng* tKO mice express no *Fng* genes. Each symbol in the graphs represents activated T cells from one mouse. Error bars reflect +/- SD. **C.** Cell lysates form preT

2017 cells or activated T cells were analyzed by western blotting using anti-N1 ECD antibody (Green) and Tubulin (Red) as a loading control. Molecular weight markers are shown on the left in kDa. Migration positions of full-length N1 (N1 FL), N1 ECD (NECD), and Tubulin (TUB) are shown on the right. N1 from activated T cells was mostly the cell surface, furin-cleaved form. N1 from preT 2017 cells was mostly the uncleaved ER form.

Figure S5: EICs showing relative levels of *O*-fucose glycoforms on peptides from N1 EGF repeats isolated from activated T cells expressing the three Fringes (*Fng* LMR) or no Fringes (*Fng* tKO).

Each EIC was generated using Xcalibur as described in Experimental Procedures. Black, red, blue, green, and magenta lines indicate the unmodified, monosaccharide, disaccharide, trisaccharide, and tetrasaccharide *O*-fucose glycoforms, respectively, of peptides from the indicated EGF. Mass spectral data can be found in Figure S11 and Tables S3, 4, and 5.

Figure S6: EICs showing relative levels of *O*-fucose glycoforms of peptides from N1 EGF repeats isolated from activated T cells expressing LFNG only, MFNG only, or RFNG only.

Each EIC was generated by Xcalibur as described in Experimental Procedures. Black, red, blue, green, and magenta lines indicate the unmodified, monosaccharide, disaccharide, trisaccharide, and tetrasaccharide *O*-fucose glycoforms, respectively, of peptides from the indicated EGF. Mass spectral data can be found in Figure S12 and Tables S6, 7, and 8.

Figure S7: Unknown modifications of *O*-fucose on EGF12 peptide can be followed by measuring reduction in the *O*-fucose monosaccharide form

Mass spectral analysis of the *O*-fucose monosaccharide glycoform of EGF12 peptide from overexpressed N1 without LFNG (**A**) and with overexpressed LFNG (**B**) using PRM as described in Experimental Procedures. Different numbers of cells (left to right is low to high numbers) were analyzed to determine how the amount of sample affected the analysis. Black line is a control peptide (QCICMPGYEGVY) from N1 without glycosylation, and the red line is the EGF12 peptide with *O*-fucose monosaccharide. Mass spectral data can be found in Figure S9 and Table S1. **C**. Ratio of the area under the curve of EGF12 *O*-Fuc peptide to Control Peptide.

Figure S8: Fringe elongation of EGF16 does not alter the N1 signaling activations in cell-based assays

N1 or N1-EGF16V were expressed in CHO cells with or without *Lfng*, and the cells were co-cultured with L cells stably overexpressing DLL1 (**A**), OP9 cells stably overexpressing DLL4 (**B**), or L cells stably overexpressing JAG1 (**C**). N1 activation was measured as described in Experimental Procedures. Blue, red, blue with shade, red with shade indicate the N1, N1-EGF16V, N1 with LFNG, and N1-EGF16V with LFNG, respectively. Average of three biological replicates is shown. Error bars show SD. The lines with * above are comparing the N1

WT and EGF16V with LFNG. The * above the +LFNG samples are comparing to without LFNG. NS≥0.05, *p<0.05, **p<0.01, ***p<0.001.

Figure S9: Annotated MS/MS spectra for different glycoforms of *O*-fucosylated EGF12 peptide from overexpressed N1 isolated from HEK293T cells with or without LFNG

Overexpressed N1 was immunopurified from HEK293T cells with or without *Lfng* overexpression, digested, and analyzed by nano-LC-MS/MS as described in Experimental Procedures. Glycopeptides were identified using Byonic. **A**, unmodified peptide. **B**, monosaccharide glycoform. **C**, disaccharide glycoform. **D**, tetrasaccharide glycoform. Due to the lability of the fucose-peptide bond in HCD experiments, Byonic is frequently unable to correctly assign the *O*-fucosylated Ser/Thr residue in a peptide. All identified peptides contain the Ser/Thr in the well-described POFUT1 consensus sequence. Green arrows show the oxonium ions containing HexNAc from the Fringe modification of *O*-fucose.

Figure S10: Annotated MS/MS spectra for different glycoforms of *O*-fucosylated peptides from EGF12 and EGF16 of N1 isolated from preT 2017 cells

N1 was immunopurified from preT 2017 cells, digested, and analyzed by nano-LC-MS/MS as described in Experimental Procedures. Glycopeptides were identified using Byonic. **A**, monosaccharide glycoform of EGF12 peptide without *O*-glucose. **B**, monosaccharide glycoform of EGF12 peptide with *O*-glucose. **C**, monosaccharide glycoform of EGF16 peptide. **D**, trisaccharide glycoform of EGF16 peptide. **E**, tetrasaccharide glycoform of EGF16 peptide. Due to the lability of the fucose-peptide bond in HCD experiments, Byonic is frequently unable to correctly assign the *O*-fucosylated Ser/Thr residue in a peptide. All identified peptides contain the Ser/Thr in the well-described POFUT1 consensus sequence. Green arrows show the oxonium ions containing HexNAc from the Fringe modification of *O*-fucose

Figure S11: Annotated MS/MS spectra for all *O*-fucosylated peptides from N1 isolated from *Fng* LMR activated T cells

N1 was immunopurified from *Fng* LMR activated T cells, digested, and analyzed by nano-LC-MS/MS as described in Experimental Procedures. Glycopeptides were identified using Byonic. **A**, monosaccharide glycoform of EGF2 peptide with *O*-glucose from *Fng* LMR. **B**, monosaccharide glycoform of EGF3 peptide from *Fng* LMR. **C**, monosaccharide glycoform of EGF5 peptide from *Fng* LMR. **D**, monosaccharide glycoform of EGF6 peptide from *Fng* LMR. **E**, trisaccharide glycoform of EGF6 peptide from *Fng* LMR. **F**, monosaccharide glycoform of EGF8 peptide from *Fng* LMR. **G**, monosaccharide glycoform of EGF9 peptide from *Fng* LMR. **H**, monosaccharide glycoform of EGF12 peptide with *O*-glucose from *Fng* LMR. **I**, monosaccharide glycoform of EGF16 peptide from *Fng* tKO. **J**, tetrasaccharide glycoform of EGF16 peptide from *Fng* LMR. **K**, unmodified of EGF18 peptide from *Fng* LMR. **L**, monosaccharide glycoform of EGF21 peptide from *Fng* LMR. **M**, monosaccharide glycoform of EGF23 peptide from *Fng* LMR. **N**, monosaccharide glycoform of EGF26 peptide from *Fng* LMR. **O**, tetrasaccharide glycoform of EGF26 peptide from *Fng* LMR. **P**, monosaccharide

glycoform of EGF27 peptide with *O*-glucose from *Fng* LMR. **Q**, disaccharide glycoform of EGF27 peptide with *O*-glucose from *Fng* LMR. **R**, trisaccharide glycoform of EGF27 peptide with *O*-glucose. **S**, monosaccharide glycoform of EGF35 peptide from *Fng* LMR. **T**, monosaccharide glycoform of EGF35 peptide from *Fng* LMR. **U**, monosaccharide glycoform of EGF36 peptide with *O*-GlcNAc from *Fng* LMR. **V**, control peptide from *Fng* LMR. Due to the lability of the fucose-peptide bond in HCD experiments, Byonic is frequently unable to correctly assign the *O*-fucosylated Ser/Thr residue in a peptide. All identified peptides contain the Ser/Thr in the well-described POFUT1 consensus sequence. Green arrows show the oxonium ions containing HexNAc from the Fringe modification of *O*-fucose.

Figure S12: Annotated MS/MS spectra for glycoforms of *O*-fucosylated peptides with Fringe elongation from N1 isolated from *Lfng*-only or *Rfng*-only T cells

N1 was immunopurified from *Lfng*-only or *Rfng*-only activated T cells, digested, and analyzed by nano-LC-MS/MS as described in Experimental Procedures. Glycopeptides were identified using Byonic. **A**, tetrasaccharide glycoform of EGF16 peptide from *Lfng*-only. **B**, tetrasaccharide glycoform of EGF16 peptide from *Rfng*-only. **C**, disaccharide glycoform of EGF27 peptide from *Lfng*-only. Due to the lability of the fucose-peptide bond in HCD experiments, Byonic is frequently unable to correctly assign the *O*-fucosylated Ser/Thr residue in a peptide. All identified peptides contain the Ser/Thr in the well-described POFUT1 consensus sequence. Green arrows show the oxonium ions containing HexNAc from the Fringe modification of *O*-fucose.

Figure S1

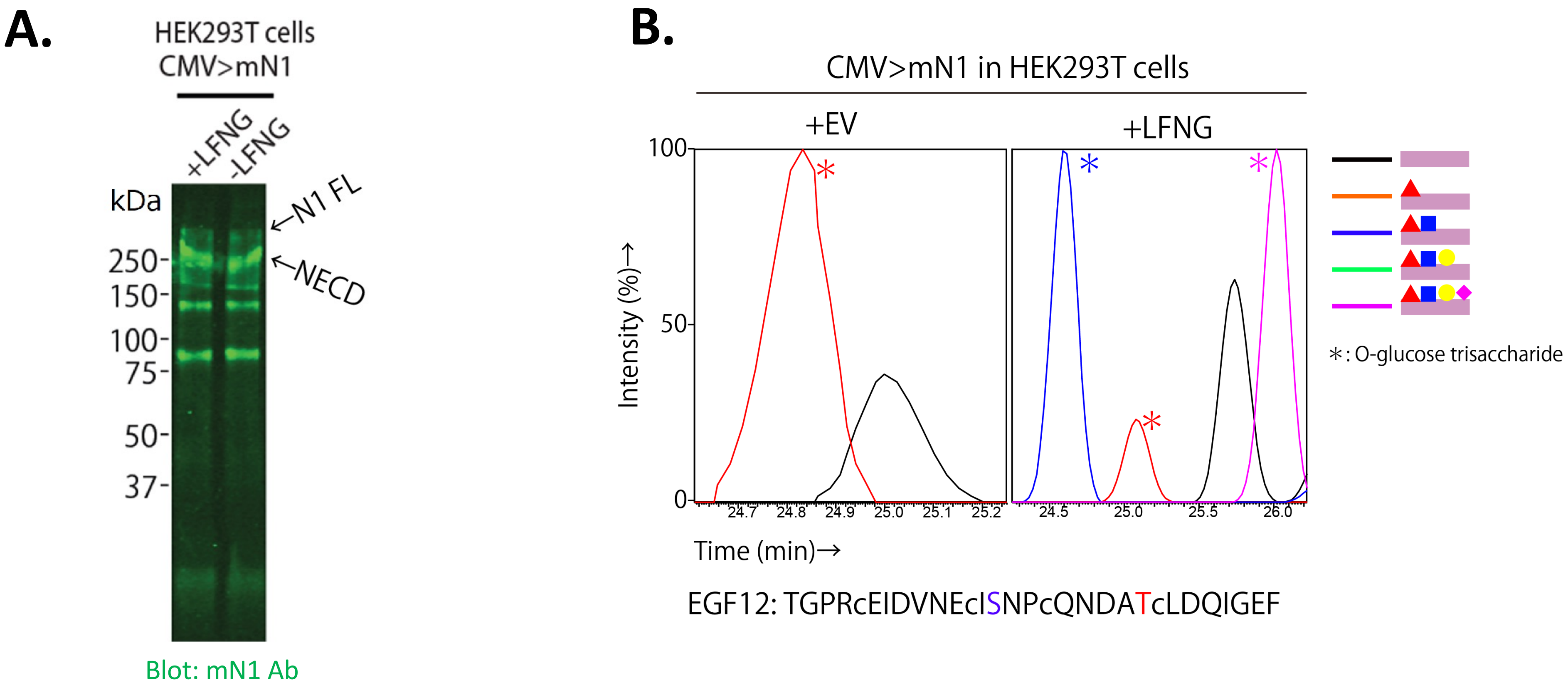
A.
HEK293T cells
CMV>mN1
+LFNG
-LFNG
kDa
250
150
100
75
50
37
←N1 FL
←NECD
Blot: mN1 Ab
B.
CMV>mN1 in HEK293T cells
+EV
+LFNG
100
50
0
Intensity (%)→
24.7
24.8
24.9
25.0
25.1
25.2
24.5
25.0
25.5
26.0
Time (min)→
*: O-glucose trisaccharide
EGF12: TGPRcEIDVNEcISNPcQNDATcLDQIGEF

Figure S2

A.

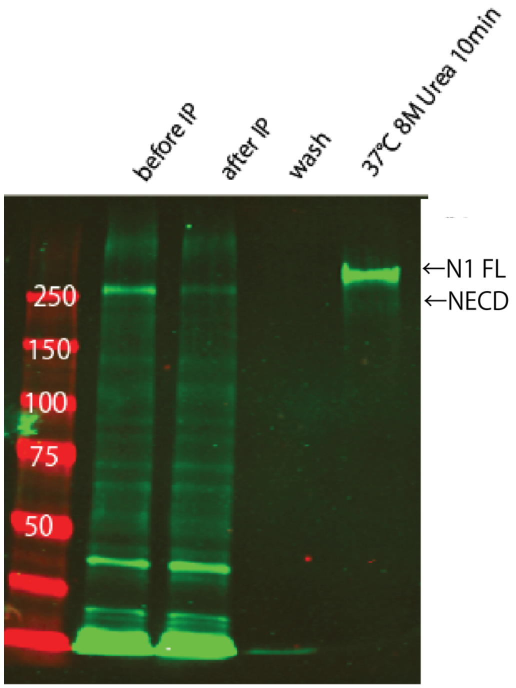

Blot: mN1 Ab

B.

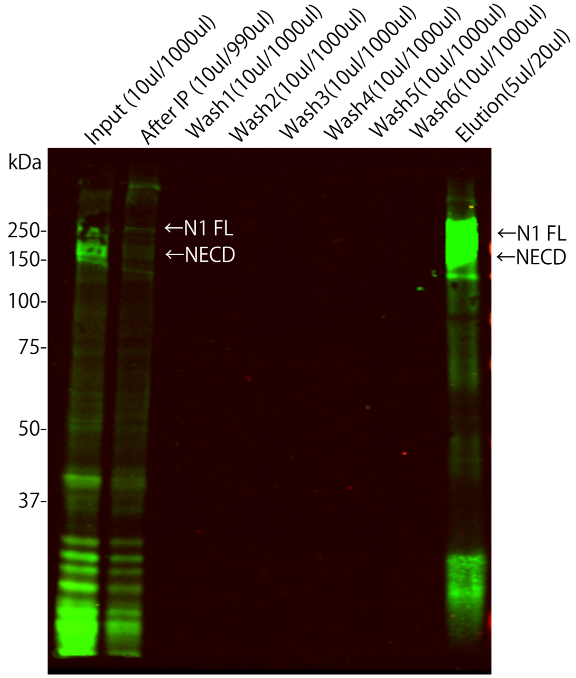

Blot: mN1 Ab

Figure S3

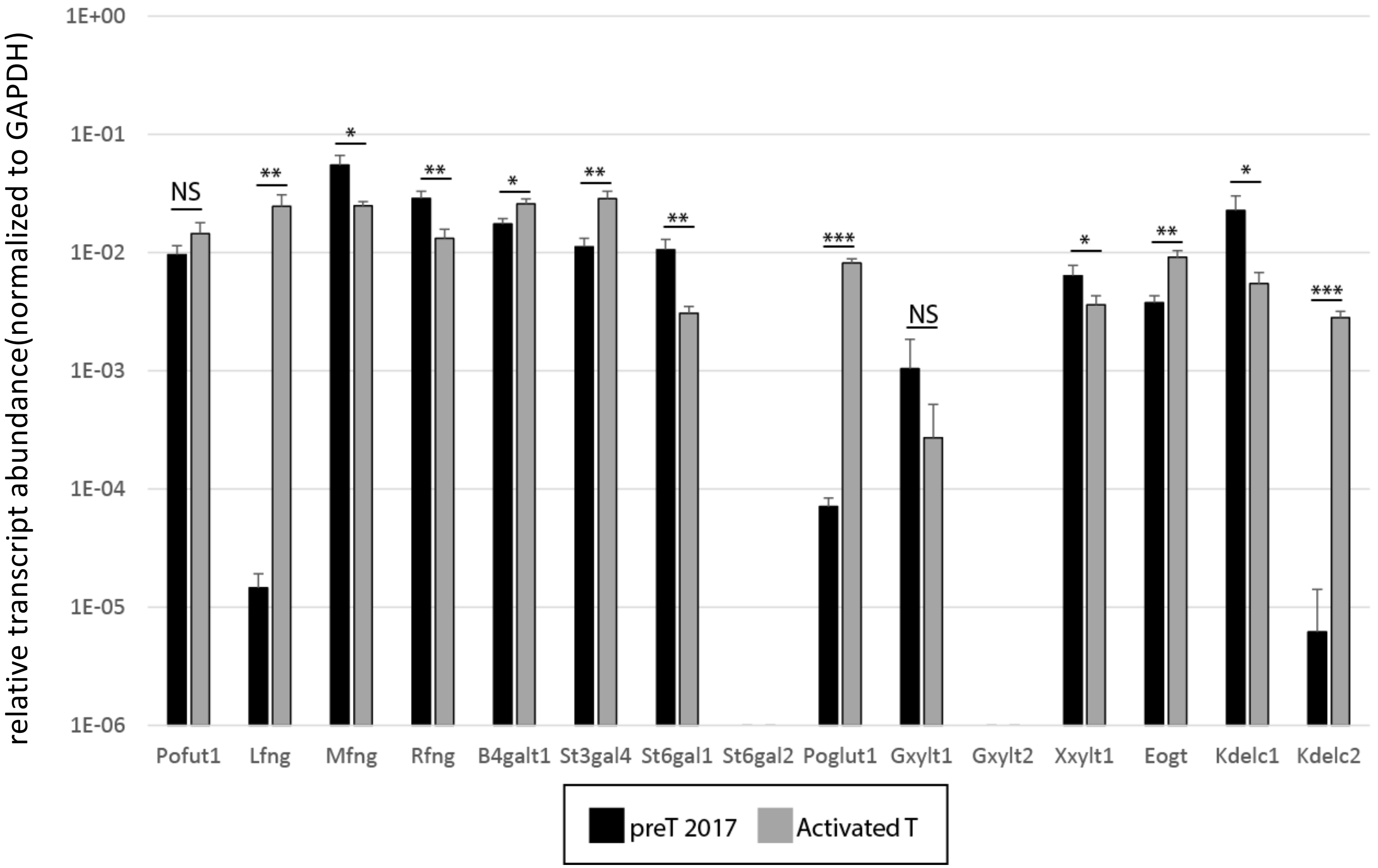

relative transcript abundance(normalized to GAPDH)
1E+00
1E-01
1E-02
1E-03
1E-04
1E-05
1E-06
NS
**
*
**
*
**
**
***
NS
*
**
*
***
Pofut1
Lfng
Mfng
Rfng
B4galt1
St3gal4
St6gal1
St6gal2
Poglut1
Gxylt1
Gxylt2
Xxylt1
Eogt
Kdelc1
Kdelc2
preT 2017
Activated T

Figure S4

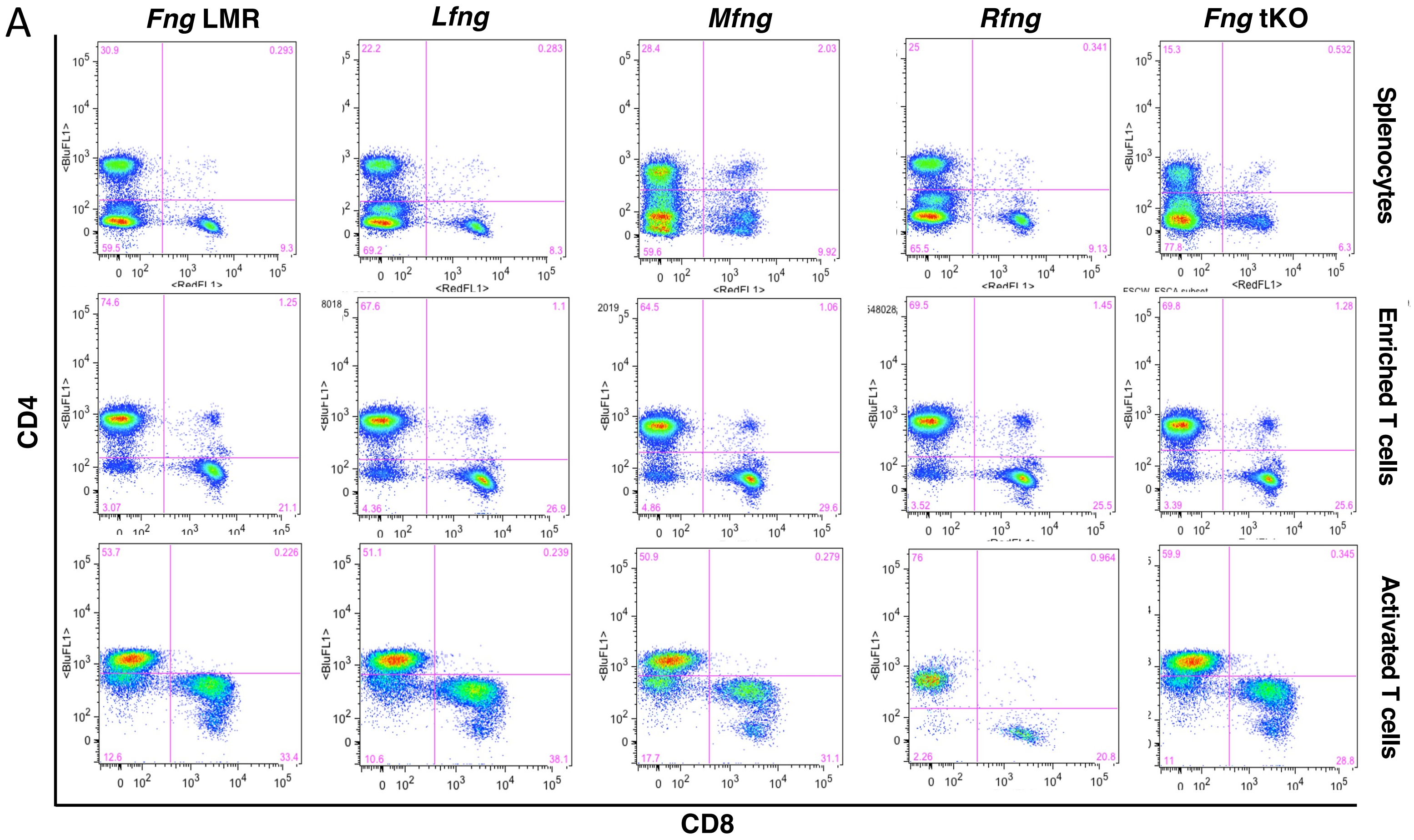

A
Fng LMR
Lfng
Mfng
Rfng
Fng tKO
Splenocytes
Enriched T cells
Activated T cells
CD4
CD8

Figure S4

B

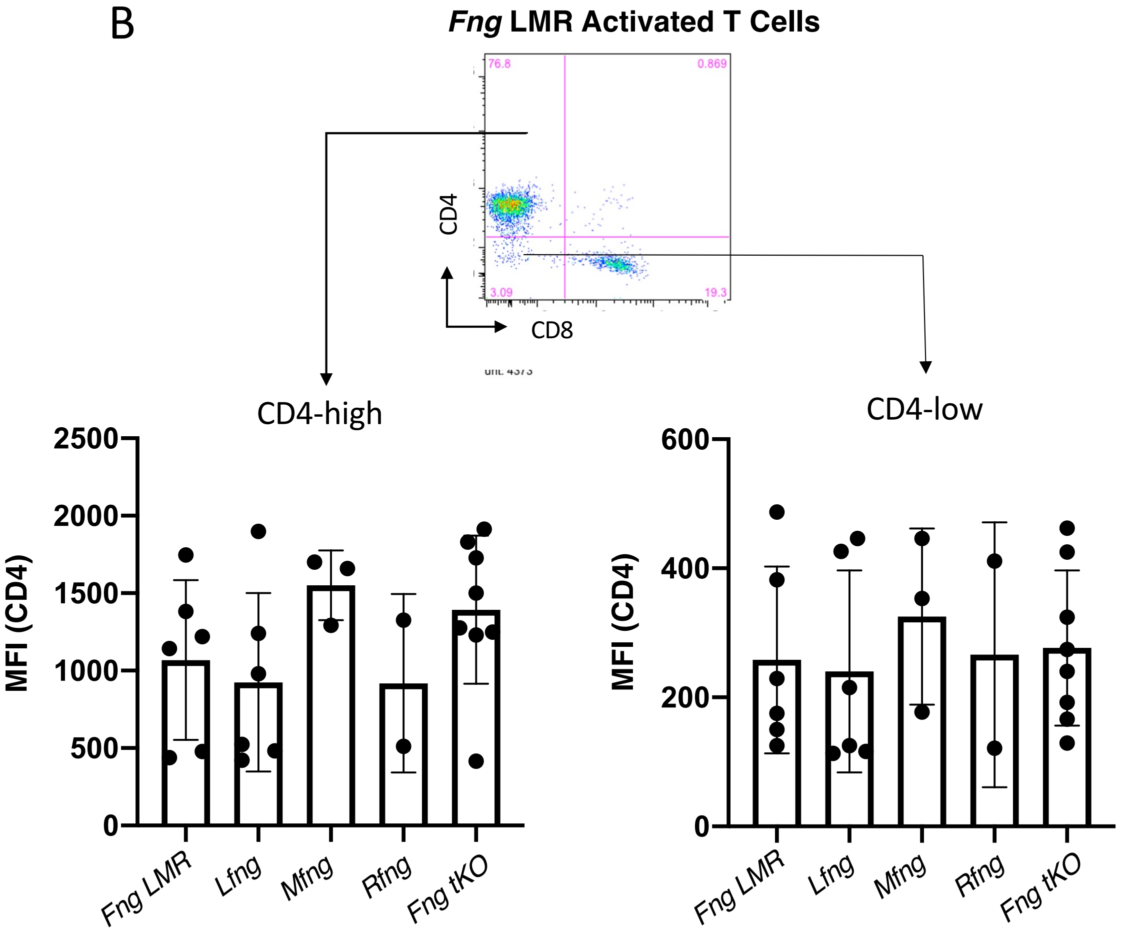

Fng LMR Activated T Cells
76.8
0.869
3.09
19.3
CD4
CD8
CD4-high
CD4-low
MFI (CD4)
2500
2000
1500
1000
500
0
600
400
200
Fng LMR
Lfng
Mfng
Rfng
Fng tKO

C

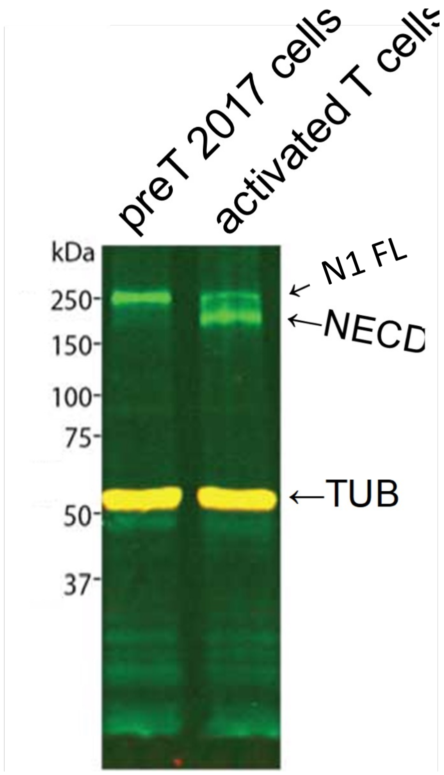

preT 2017 cells
activated T cells
kDa
250
150
100
75
50
37
N1 FL
NECD
TUB

Figure S5

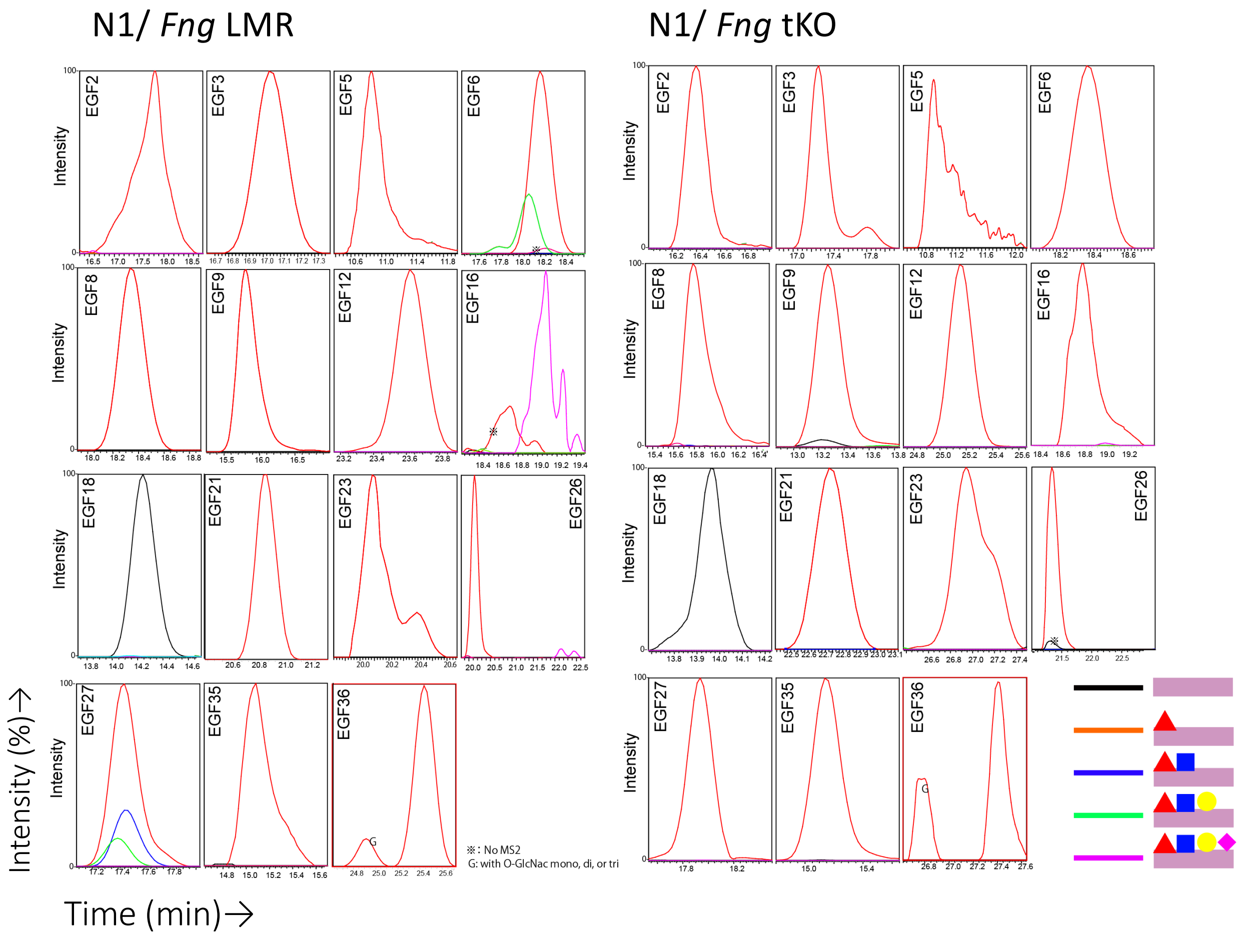
N1/ Fng LMR
N1/ Fng tKO
EGF2
EGF3
EGF5
EGF6
EGF8
EGF9
EGF12
EGF16
EGF18
EGF21
EGF23
EGF26
EGF27
EGF35
EGF36
Intensity
Intensity (%)→
Time (min)→
※: No MS2
G: with O-GlcNac mono, di, or tri

Figure S6

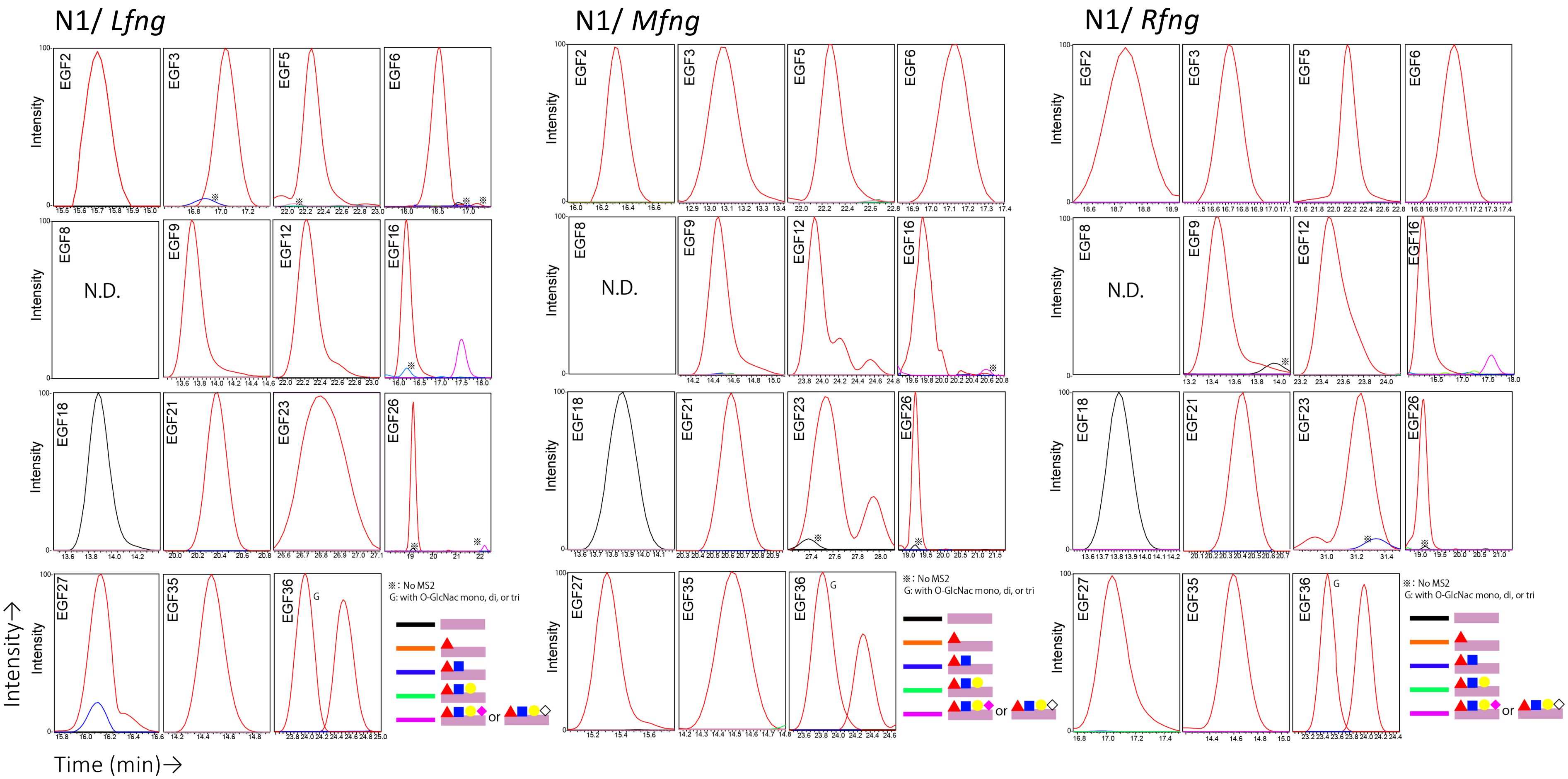
N1/ Lfng
N1/ Mfng
N1/ Rfng
EGF2
EGF3
EGF5
EGF6
EGF8
EGF9
EGF12
EGF16
EGF18
EGF21
EGF23
EGF26
EGF27
EGF35
EGF36
N.D.
Intensity
G
※: No MS2
G: with O-GlcNac mono, di, or tri
or
Intensity→
Time (min)→

Figure S7

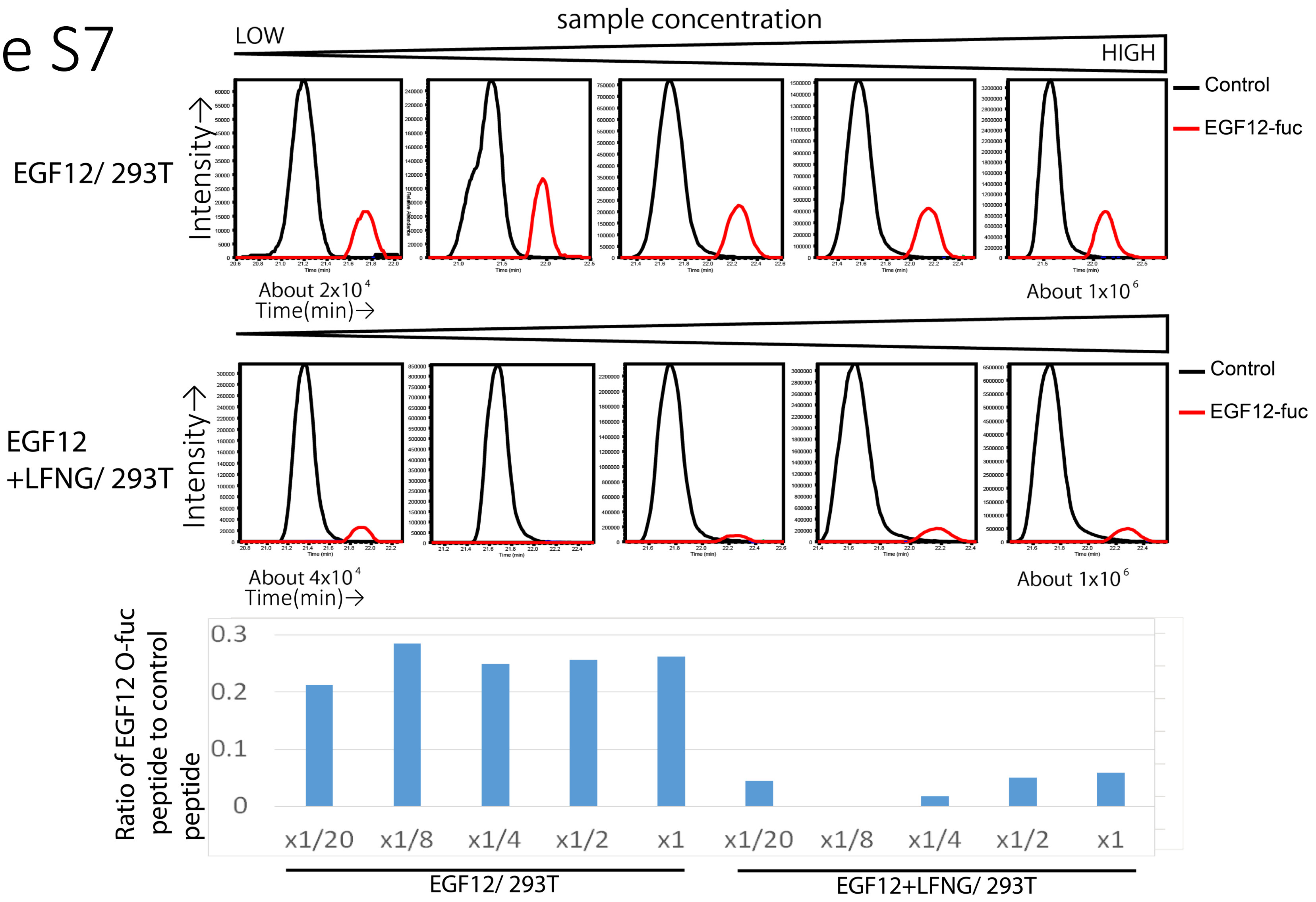
sample concentration
LOW
HIGH
Control
EGF12-fuc
EGF12/ 293T
Intensity→
About 2x10 4
Time(min)→
About 1x10 6
Control
EGF12-fuc
EGF12
+LFNG/ 293T
Intensity→
About 4x10 4
Time(min)→
About 1x10 6
Ratio of EGF12 O-fuc peptide to control peptide
0.3
0.2
0.1
0
x1/20
x1/8
x1/4
x1/2
x1
x1/20
x1/8
x1/4
x1/2
x1
EGF12/ 293T
EGF12+LFNG/ 293T

Figure S8

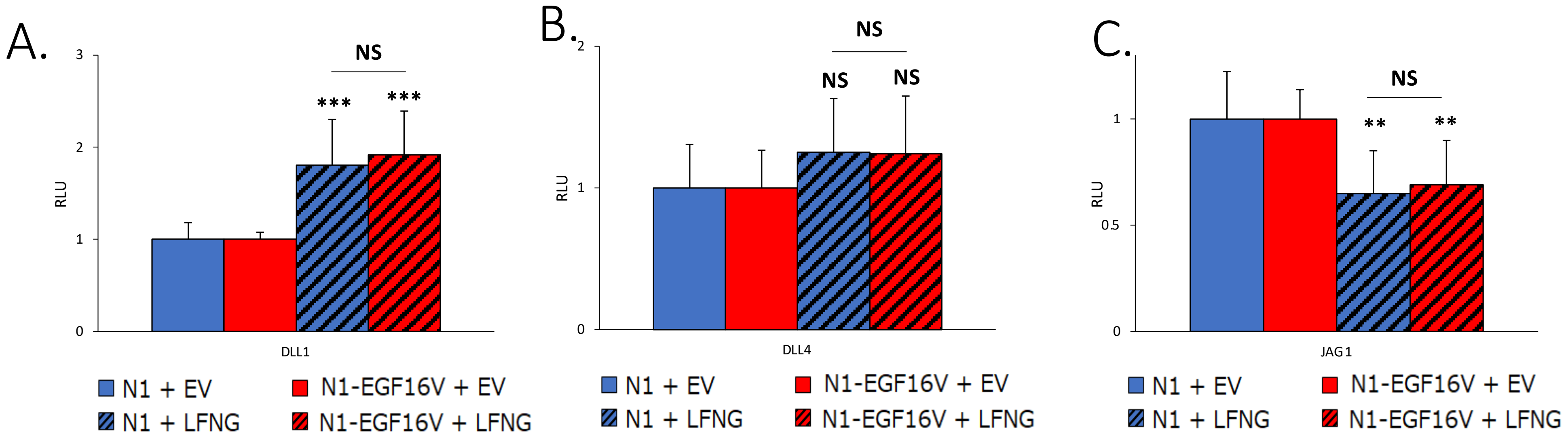

A.
3
2
1
0
RLU
NS
***
***
DLL1
N1 + EV
N1-EGF16V + EV
N1 + LFNG
N1-EGF16V + LFNG
B.
2
1
0
RLU
NS
NS
NS
DLL4
N1 + EV
N1-EGF16V + EV
N1 + LFNG
N1-EGF16V + LFNG
C.
1
0.5
0
RLU
NS
**
**
JAG1
N1 + EV
N1-EGF16V + EV
N1 + LFNG
N1-EGF16V + LFNG

Figure S9A

Unmodified EGF12 from HEK293T cells

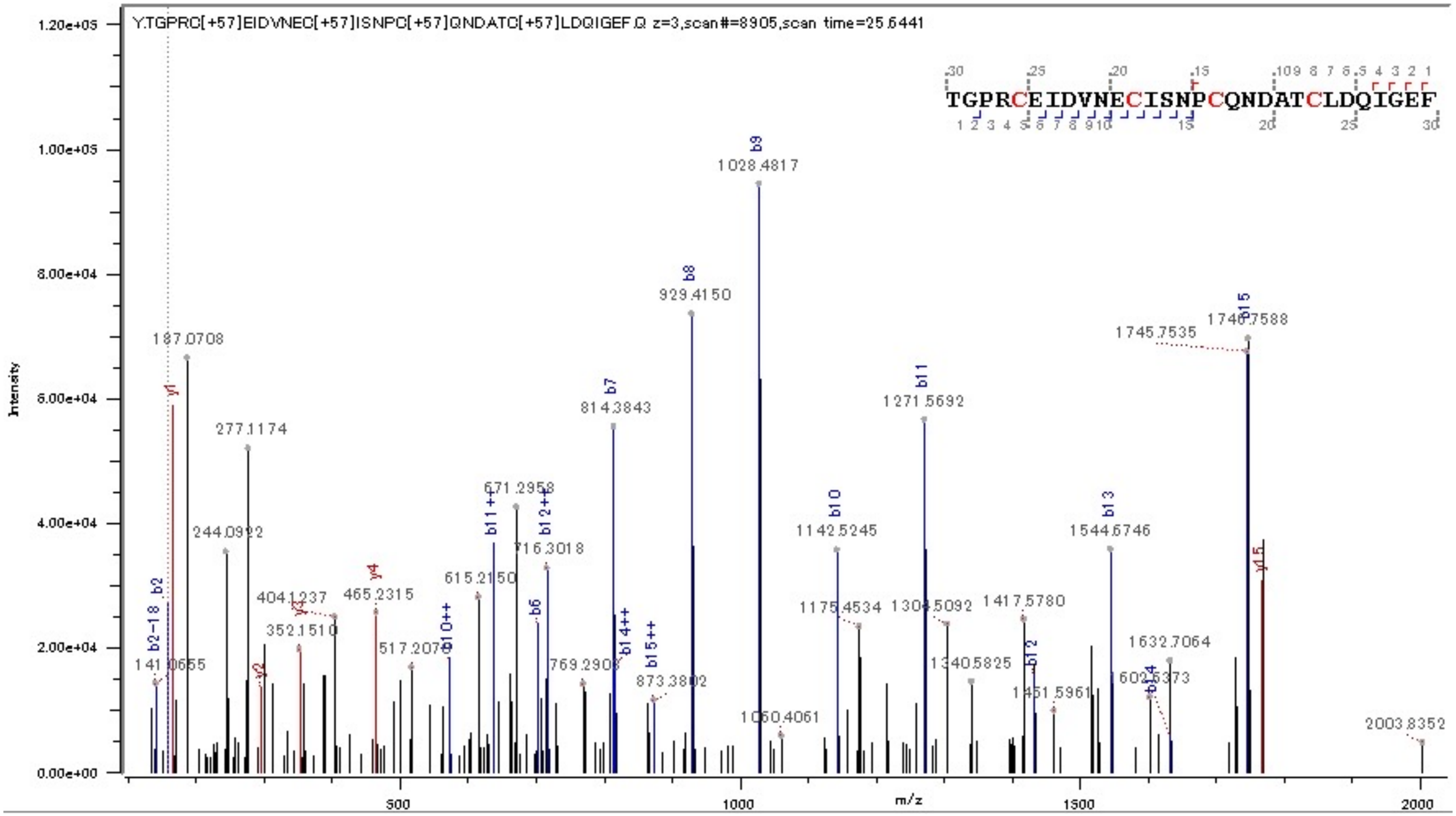

Figure S9B

Monosaccharide *O*-fucose modification of EGF12* from HEK293T cells

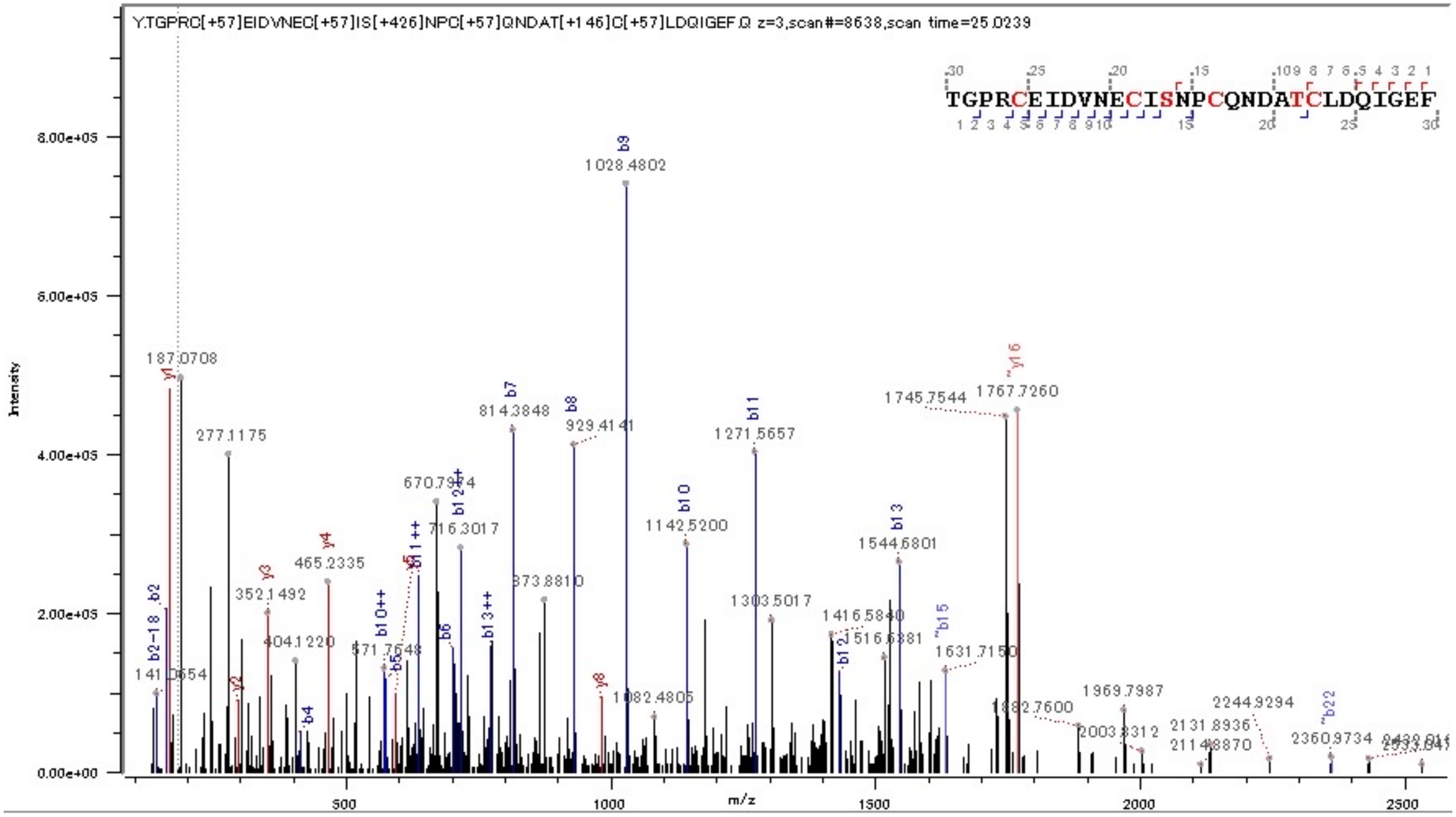

*This peptide is also modified with an *O*-glucose tetrasaccharide.

Figure S9C

Disaccharide *O*-fucose modification of EGF12* from HEK293T cells with LFNG

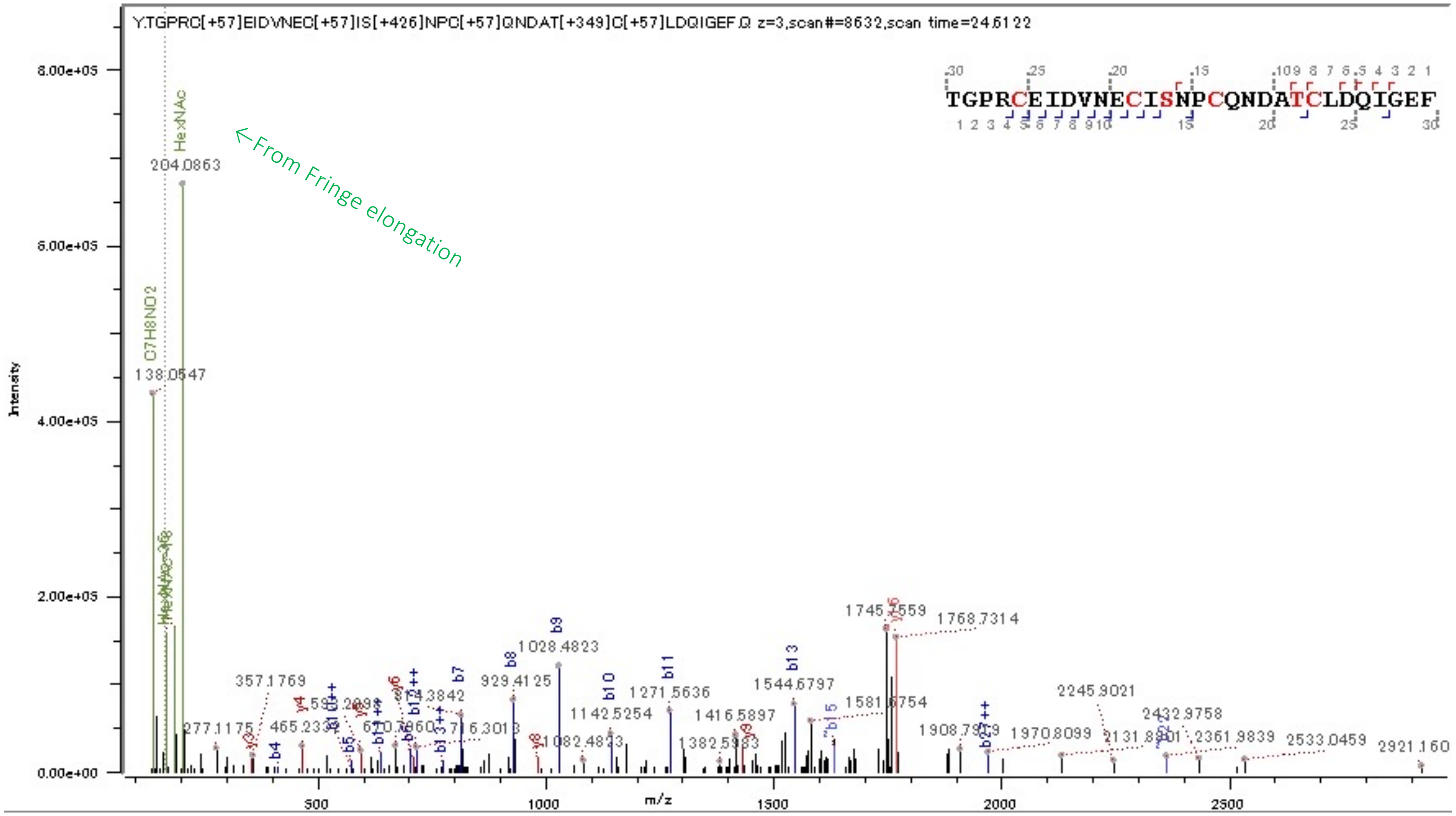

*This peptide is also modified with an *O*-glucose tetrasaccharide.

Figure S9D

Tetrasaccharide *O*-fucose modification of EGF12* from HEK293T cells with LFNG

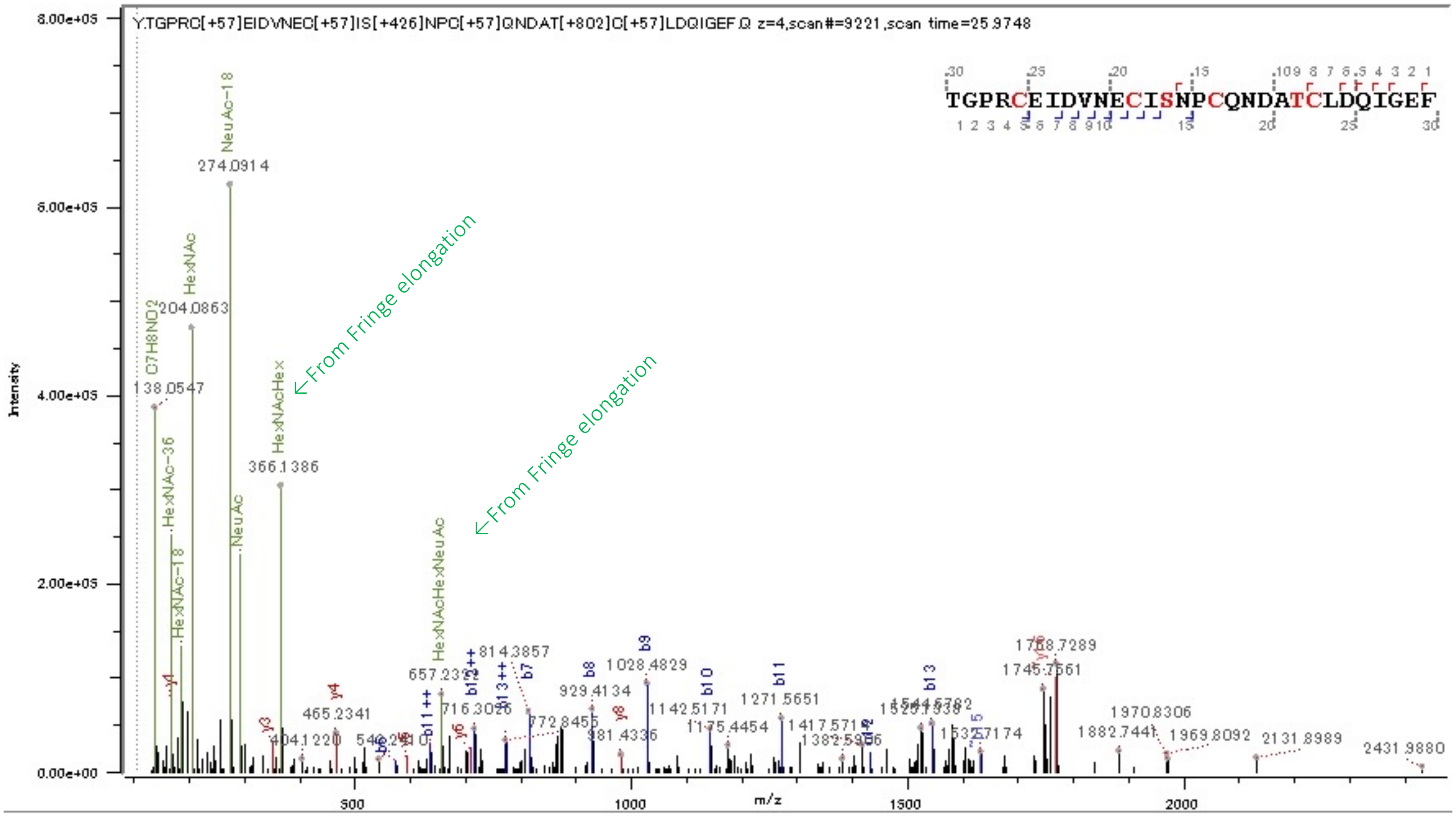

*This peptide is also modified with an *O*-glucose tetrasaccharide.

Figure S10A

O-fucose modification of EGF12 from preT 2017 cells.

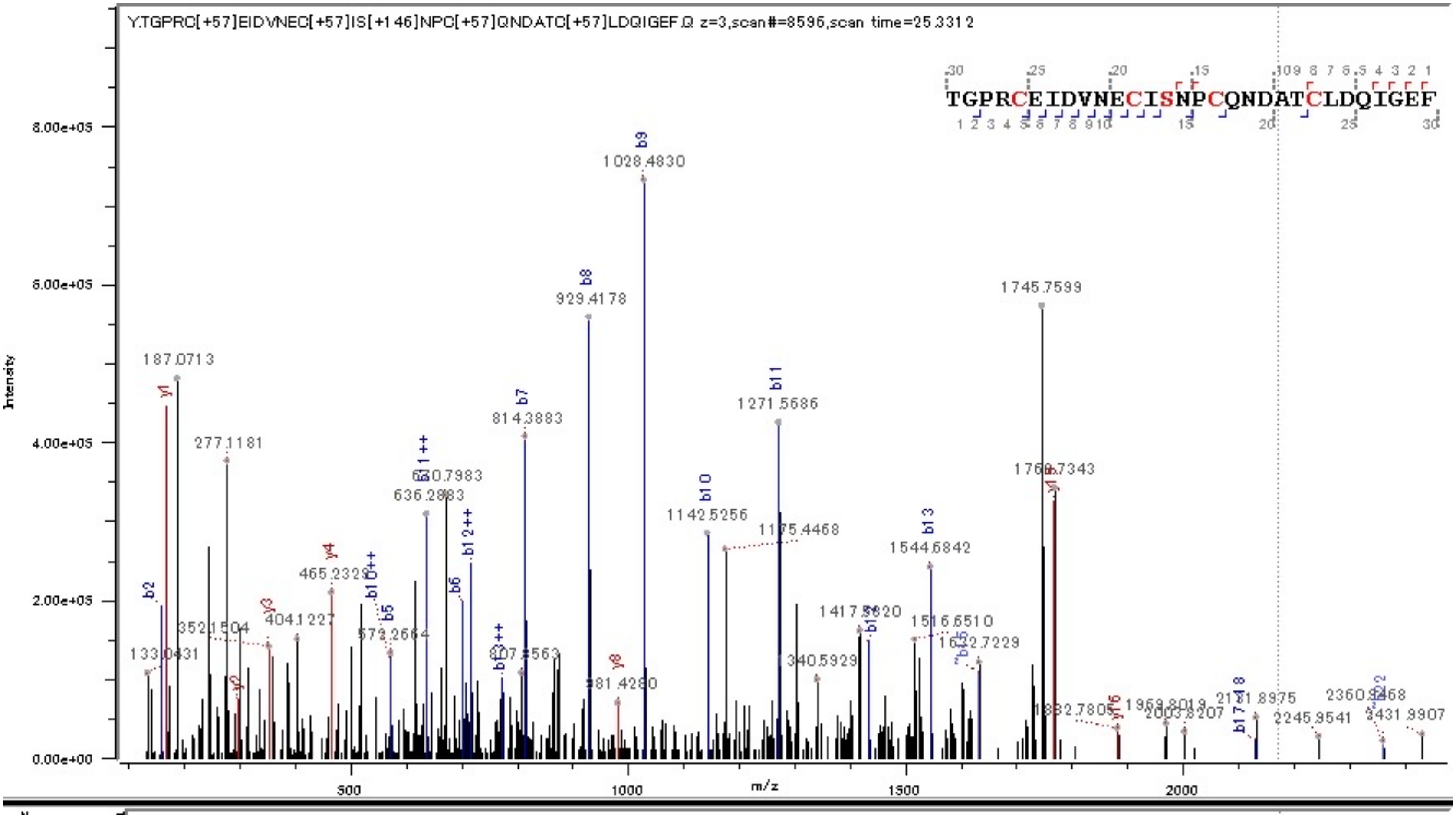

Figure S10B

O-fucose modification of EGF12 from preT 2017 cells.

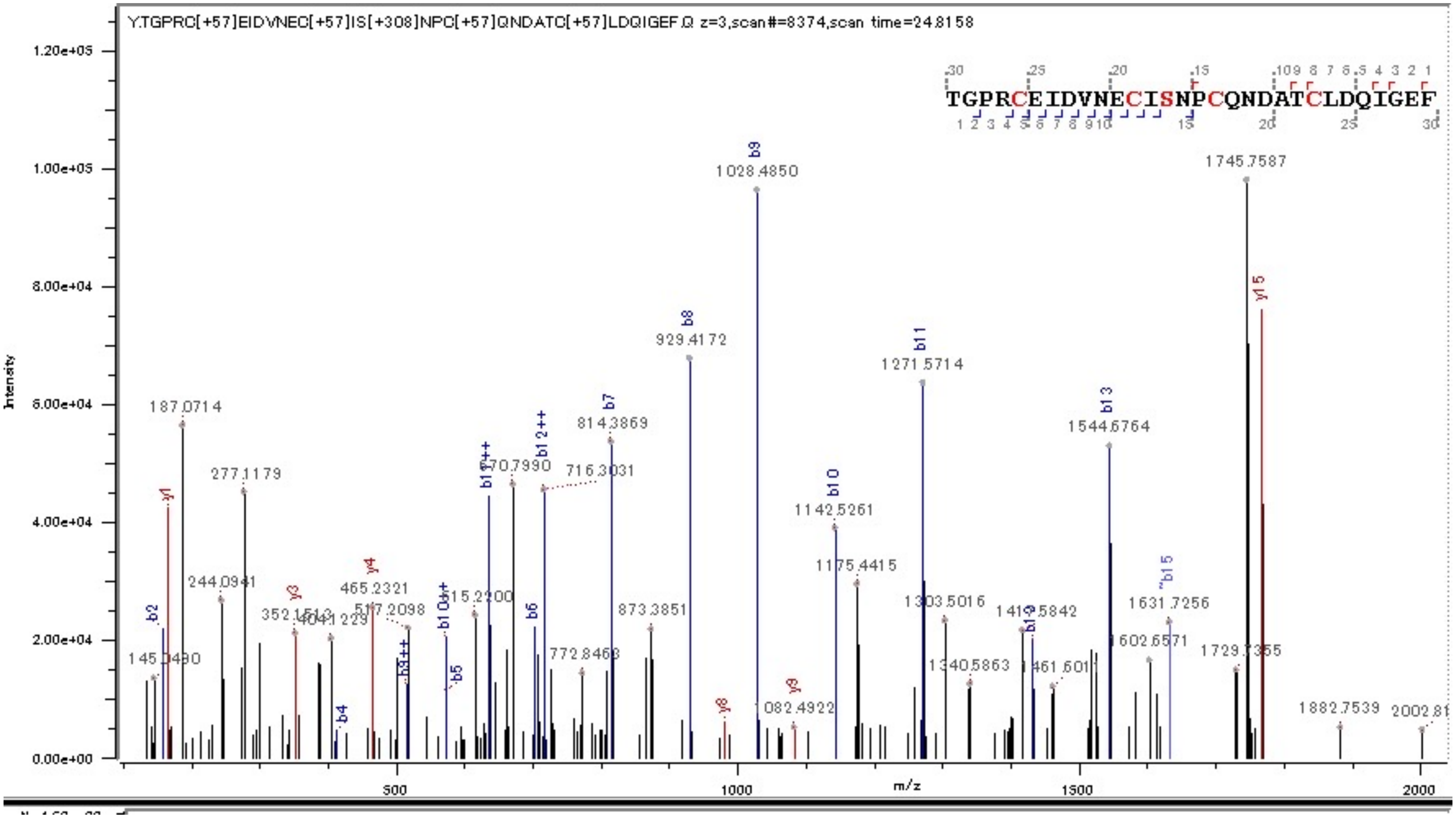

*Peptide also modified with *O*-glucose monosaccharide

Figure S10C

Monosaccharide *O*-fucose modification of EGF16 from preT 2017 cells.

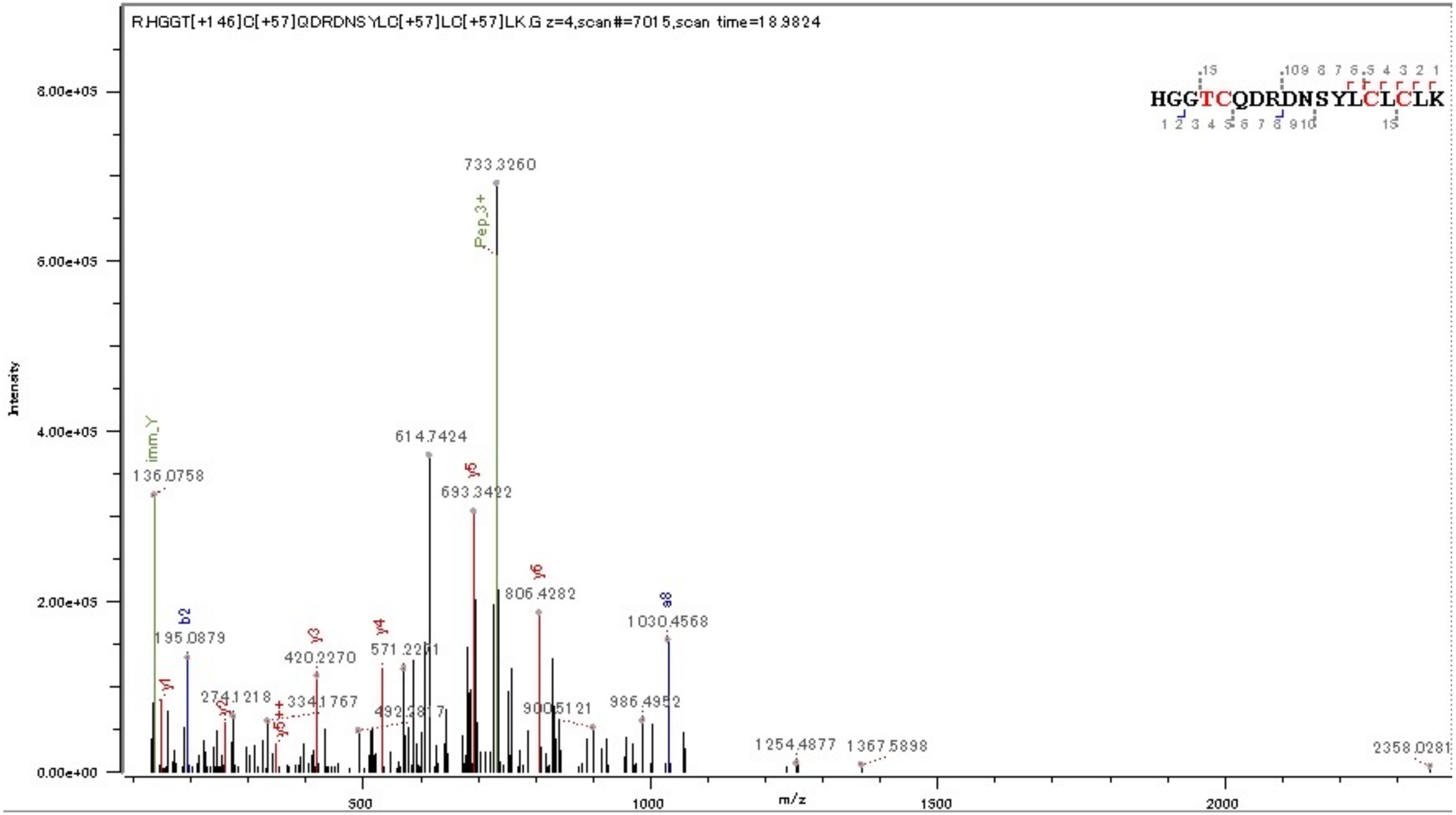

Figure S10D

Trisaccharide *O*-fucose modification of EGF16 from preT 2017 cells.

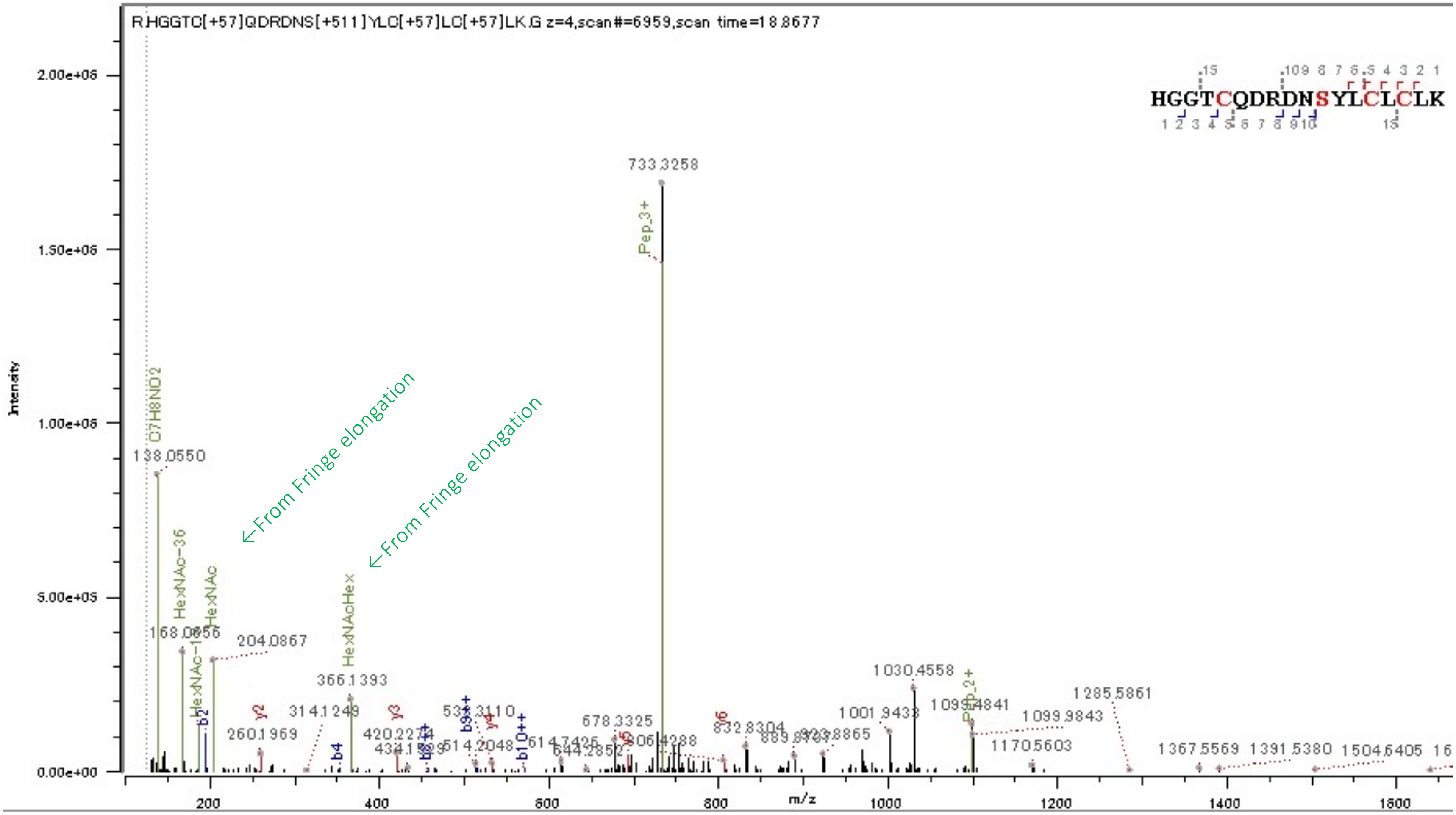

Figure S10E

Tetrasaccharide *O*-fucose modification of EGF16 from preT 2017 cells.

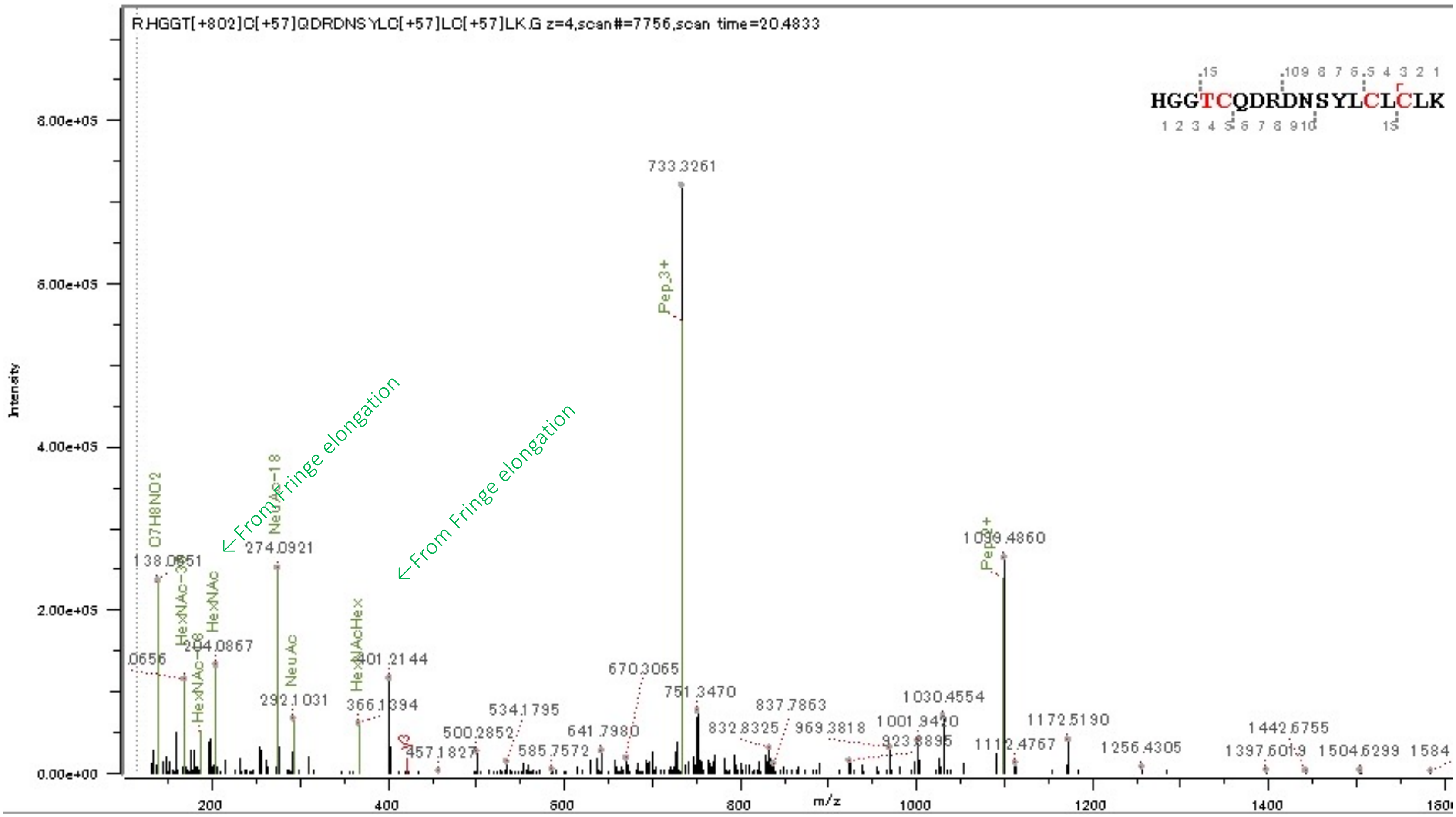

Figure S11A

Monosaccharide *O*-fucose modification of EGF2 from *Fng* LMR activated T cells

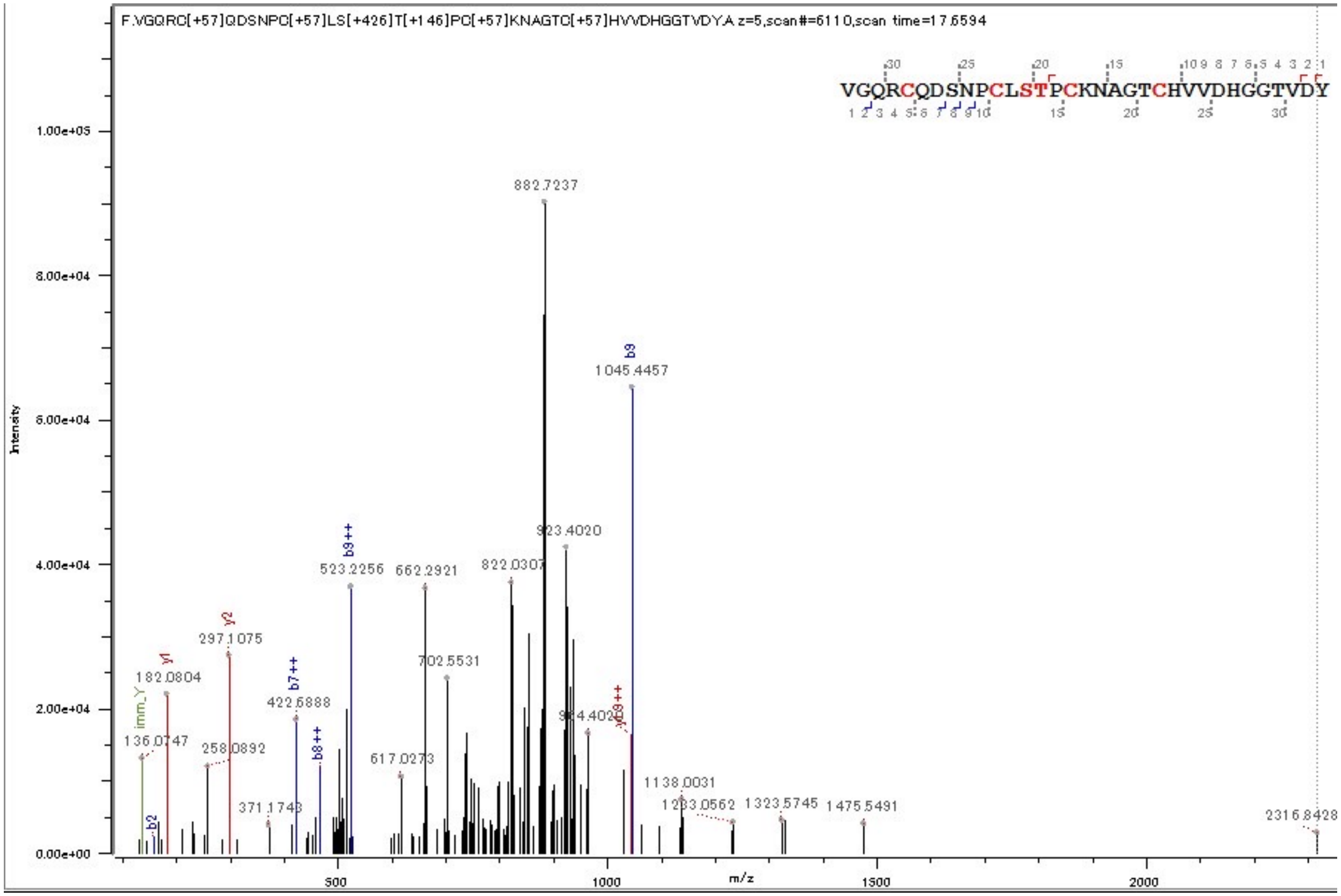

*Peptide also modified with *O*-glucose trisaccharide

Figure S11B

Monosaccharide *O*-fucose modification of EGF3 from *Fng* LMR activated T cells

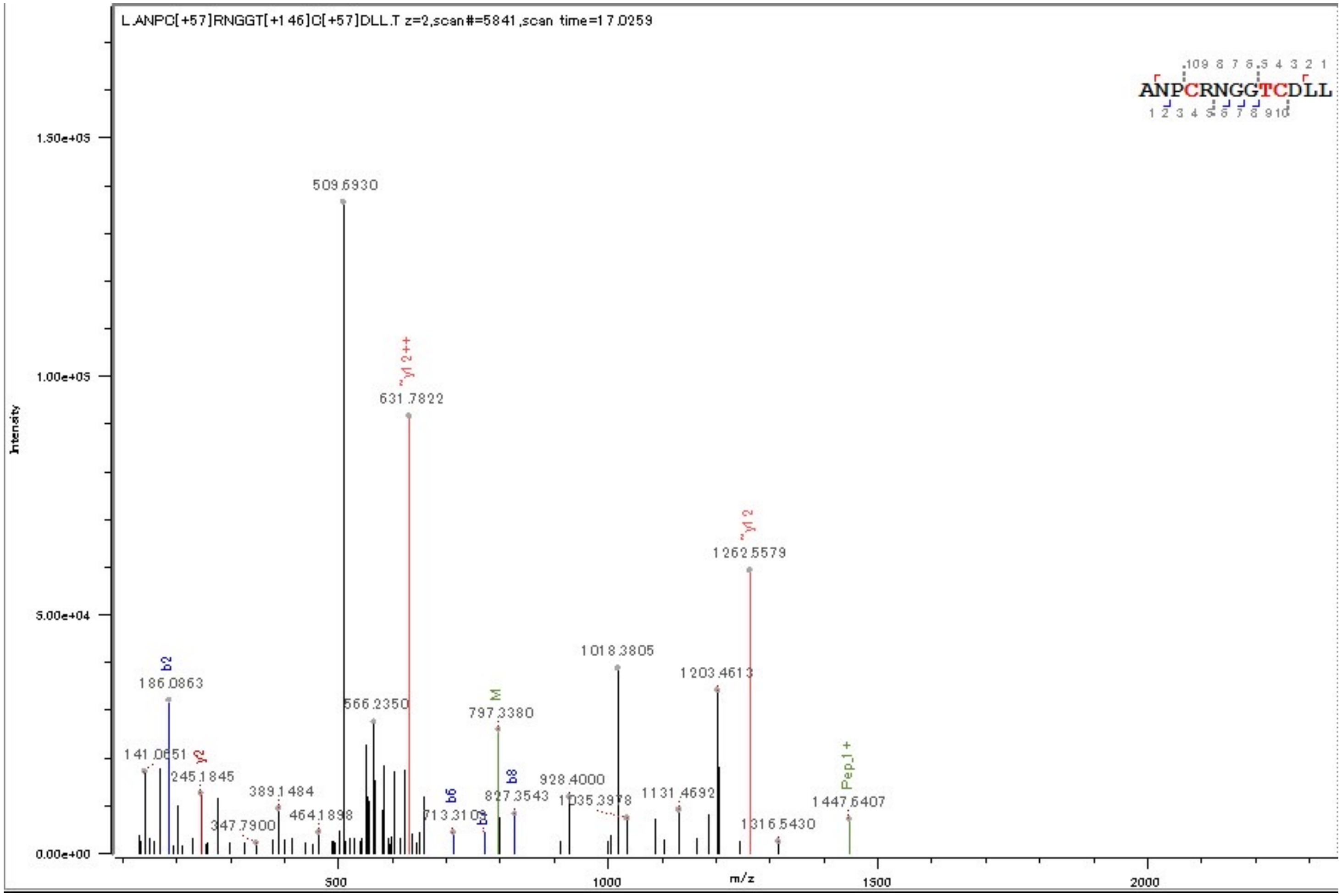

Figure S11C

Monosaccharide *O*-fucose modification of EGF5 from *Fng* LMR activated T cells

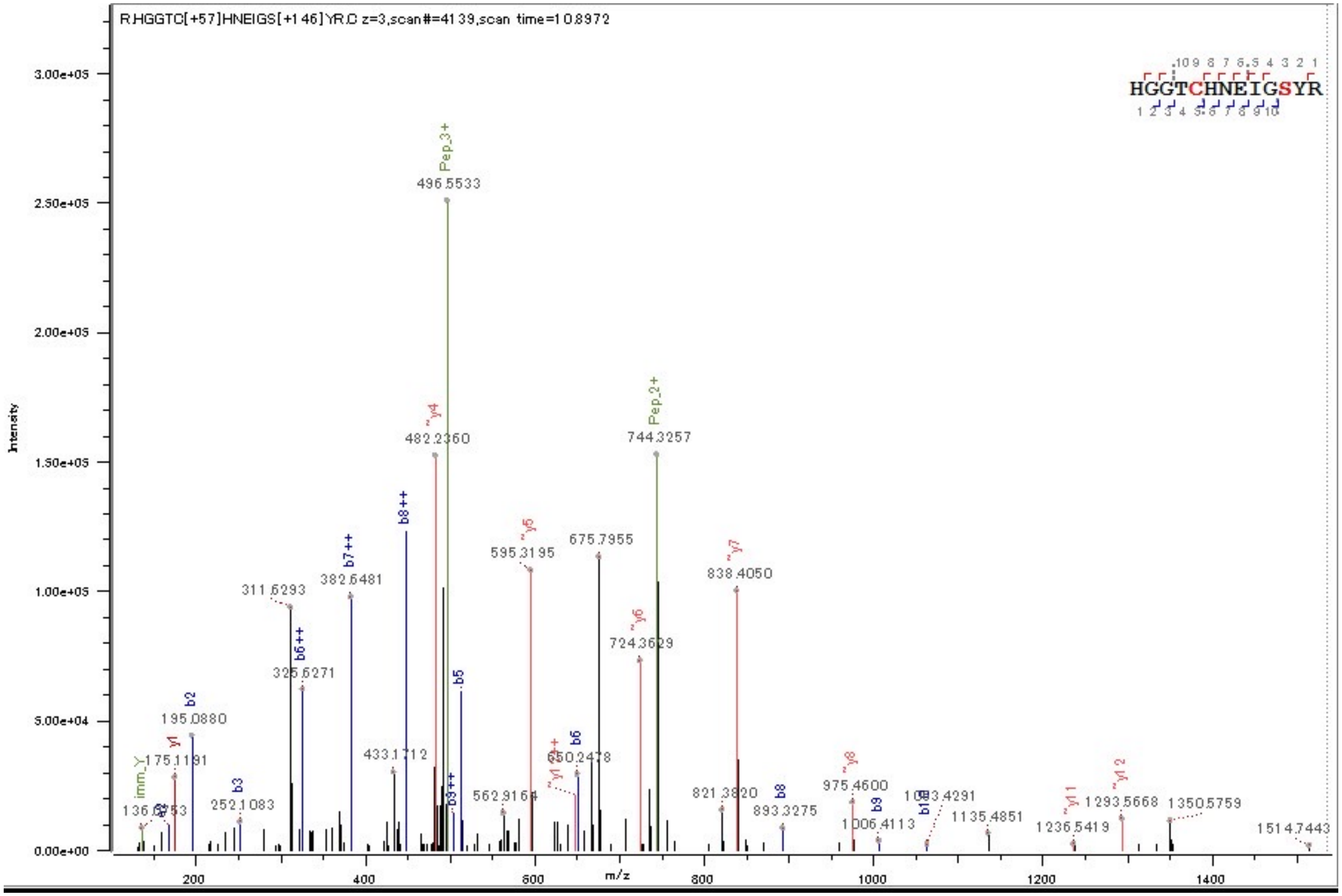

Figure S11D

Monosaccharide *O*-fucose modification of EGF6 from *Fng* LMR activated T cells

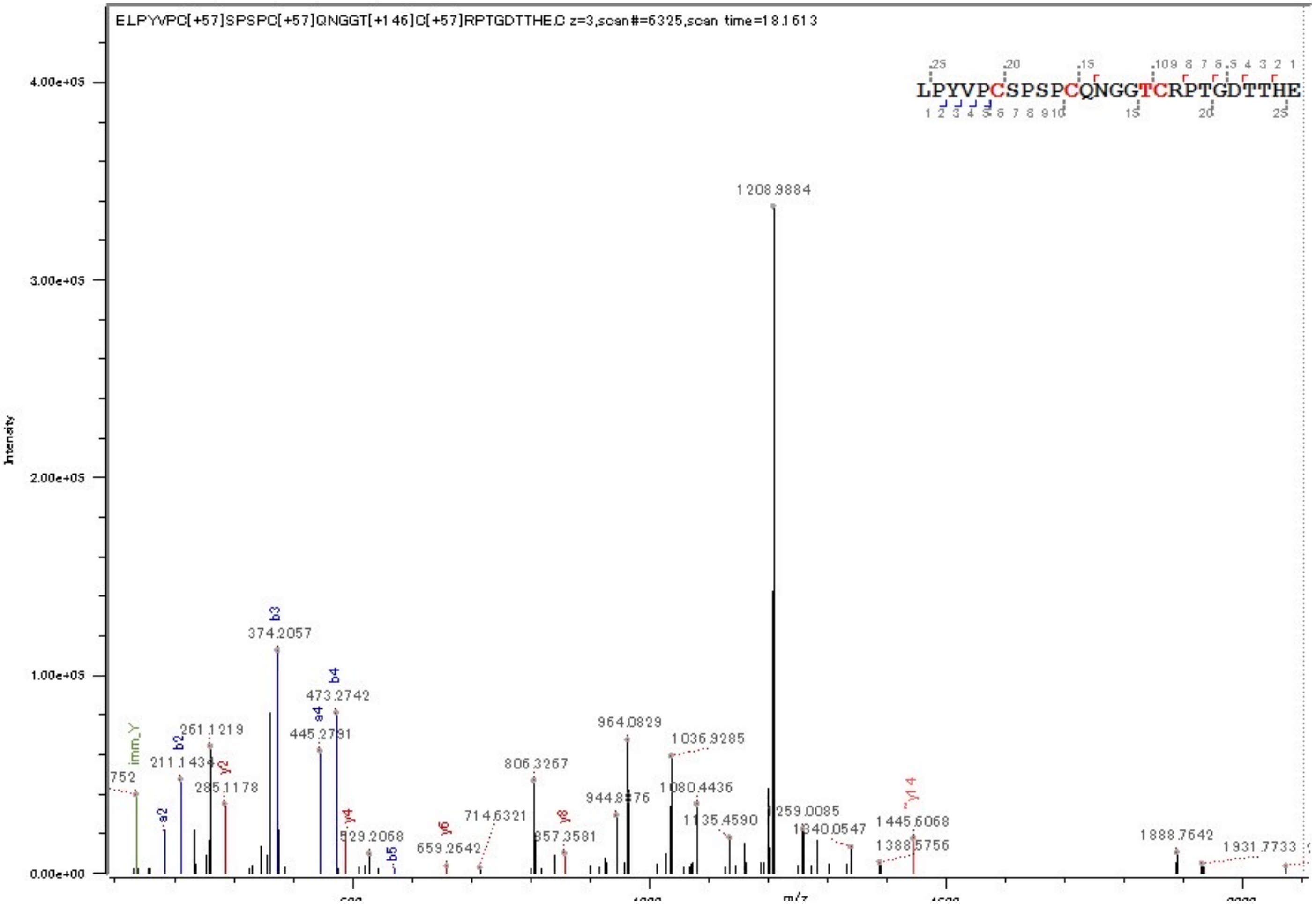

Figure S11E

Trisaccharide *O*-fucose modification of EGF6 from *Fng* LMR activated T cells

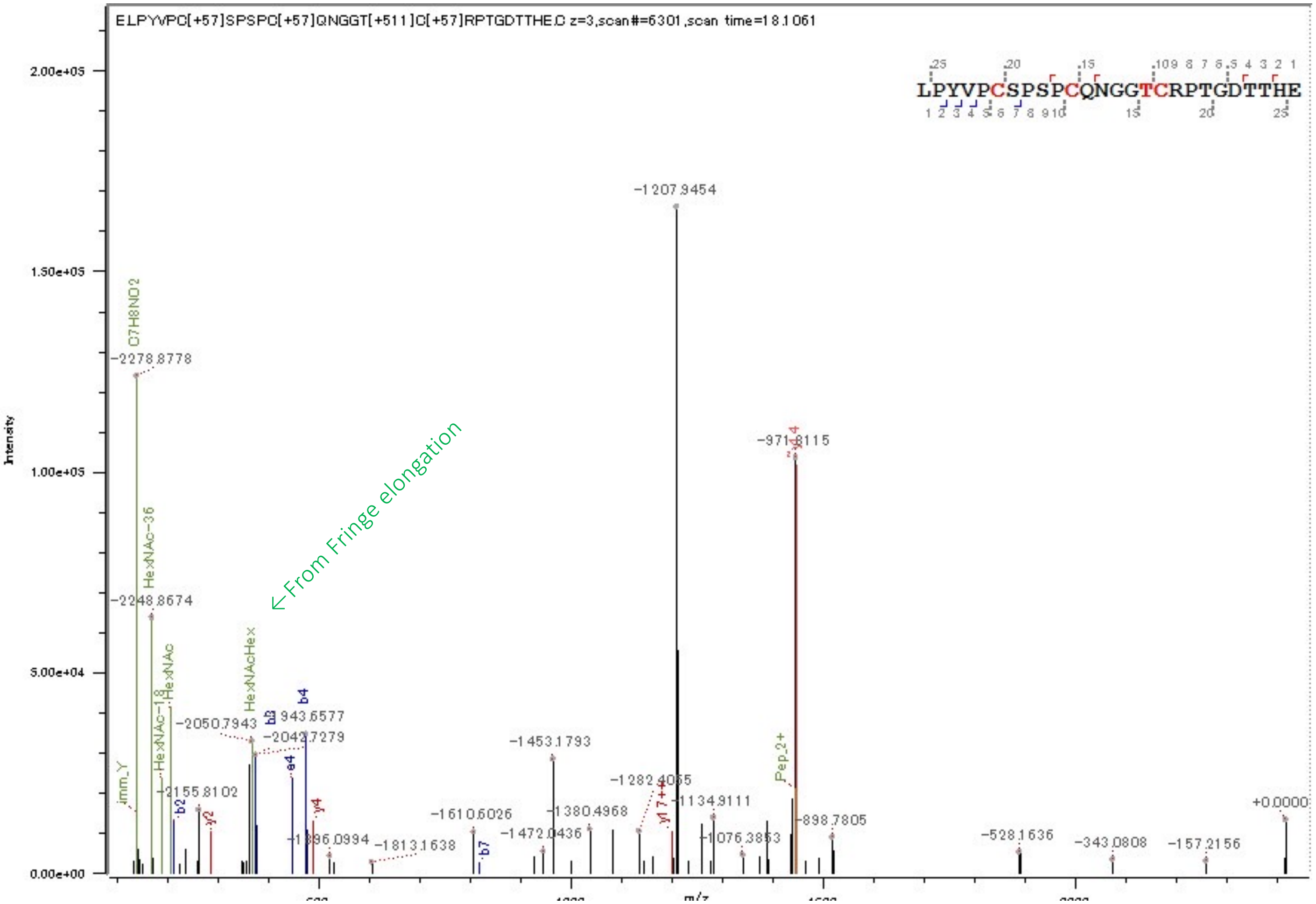

Figure S11F

Monosaccharide *O*-fucose modification of EGF8 from *Fng* LMR activated T cells

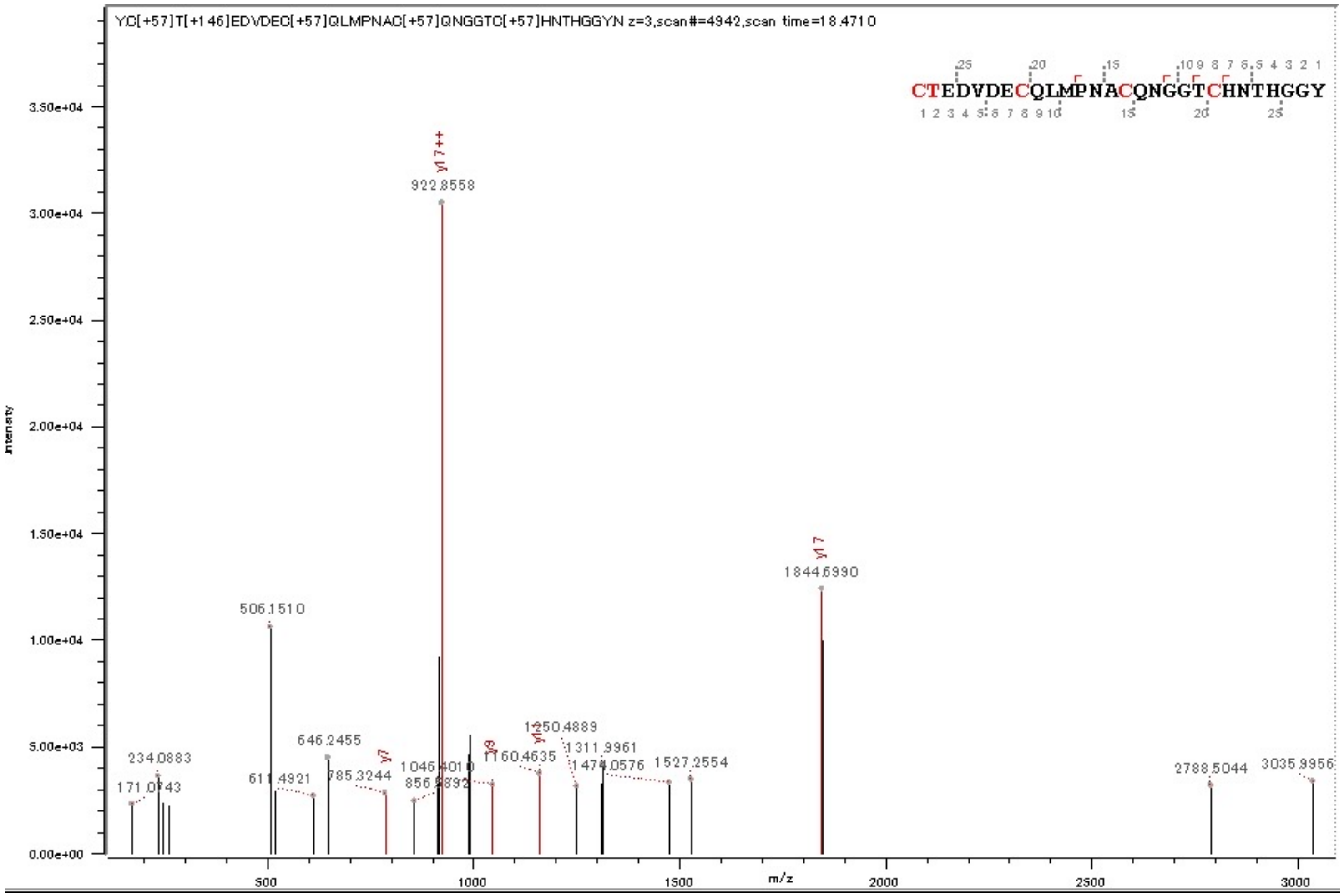

Figure S11G

Monosaccharide *O*-fucose modification of EGF9 from *Fng* LMR activated T cells

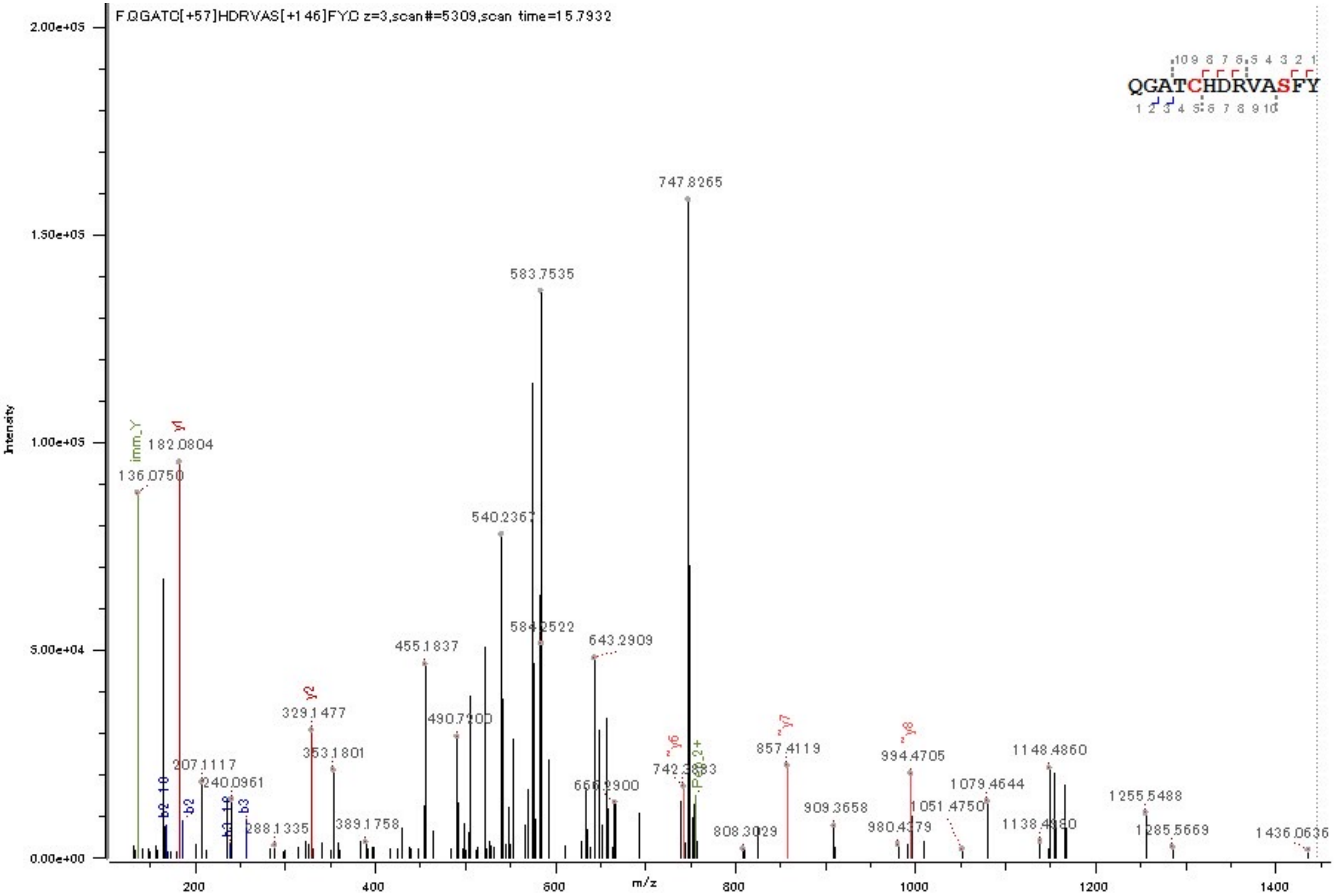

Figure S11H

Monosaccharide *O*-fucose modification of EGF12 from *Fng* LMR activated T cells

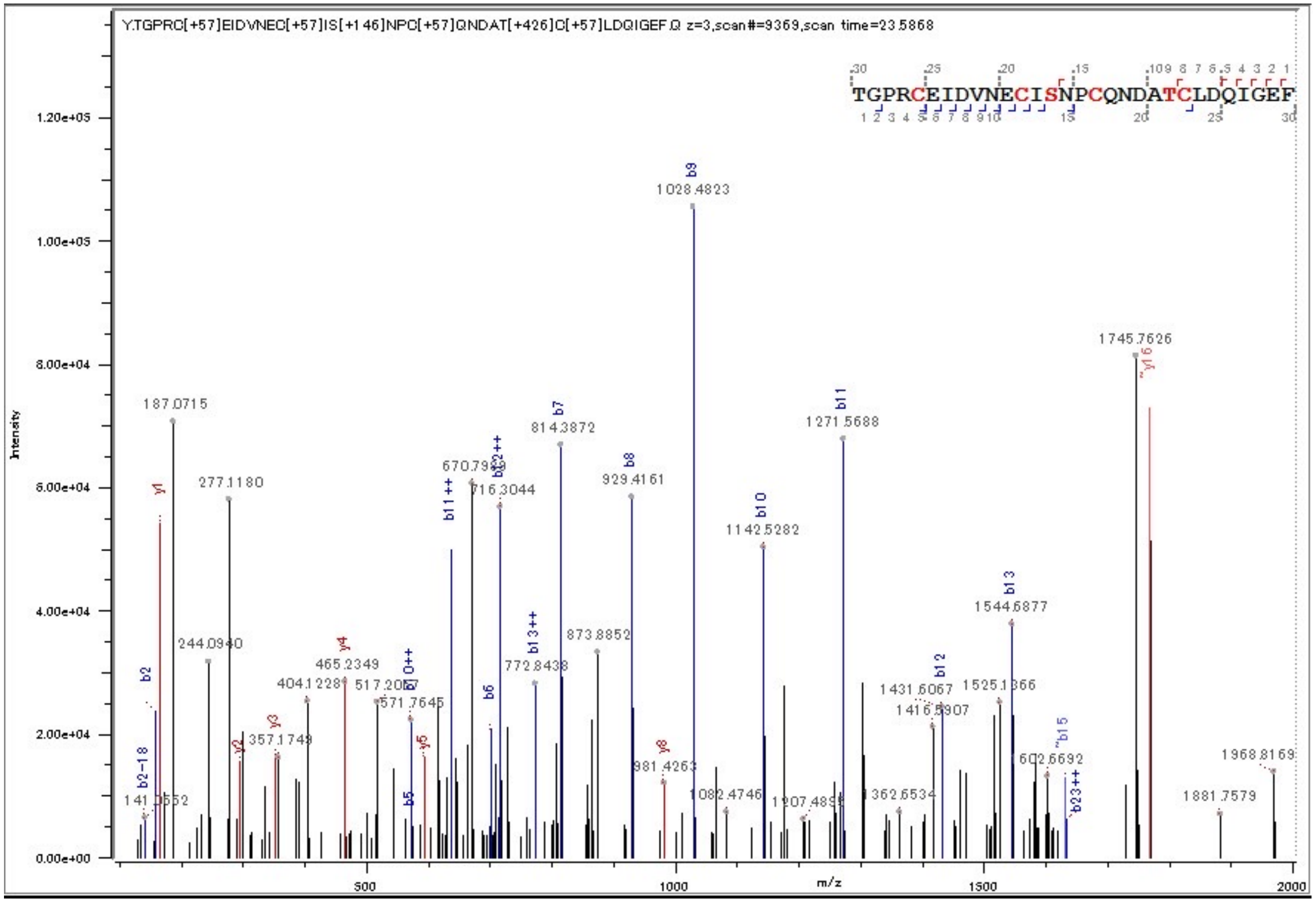

*Peptide also modified with *O*-glucose trisaccharide

Figure S11I

Monosaccharide *O*-fucose modification of EGF16 from *Fng* tKO activated T cells

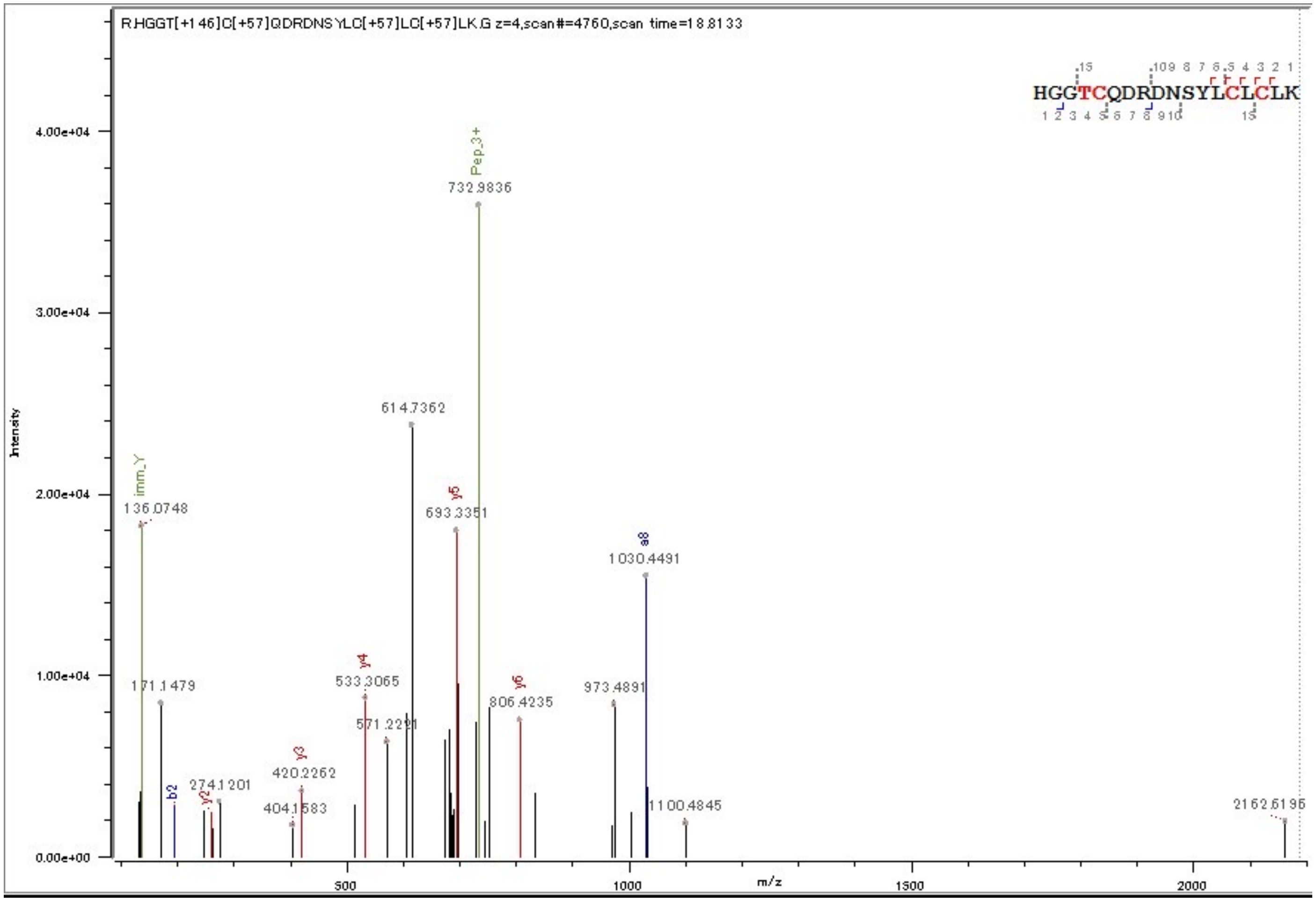

Figure S11J

Tetrasaccharide *O*-fucose (Fuc-HexNac-Hex-NeuGc) modification of EGF16 from *Fng* LMR activated T cells

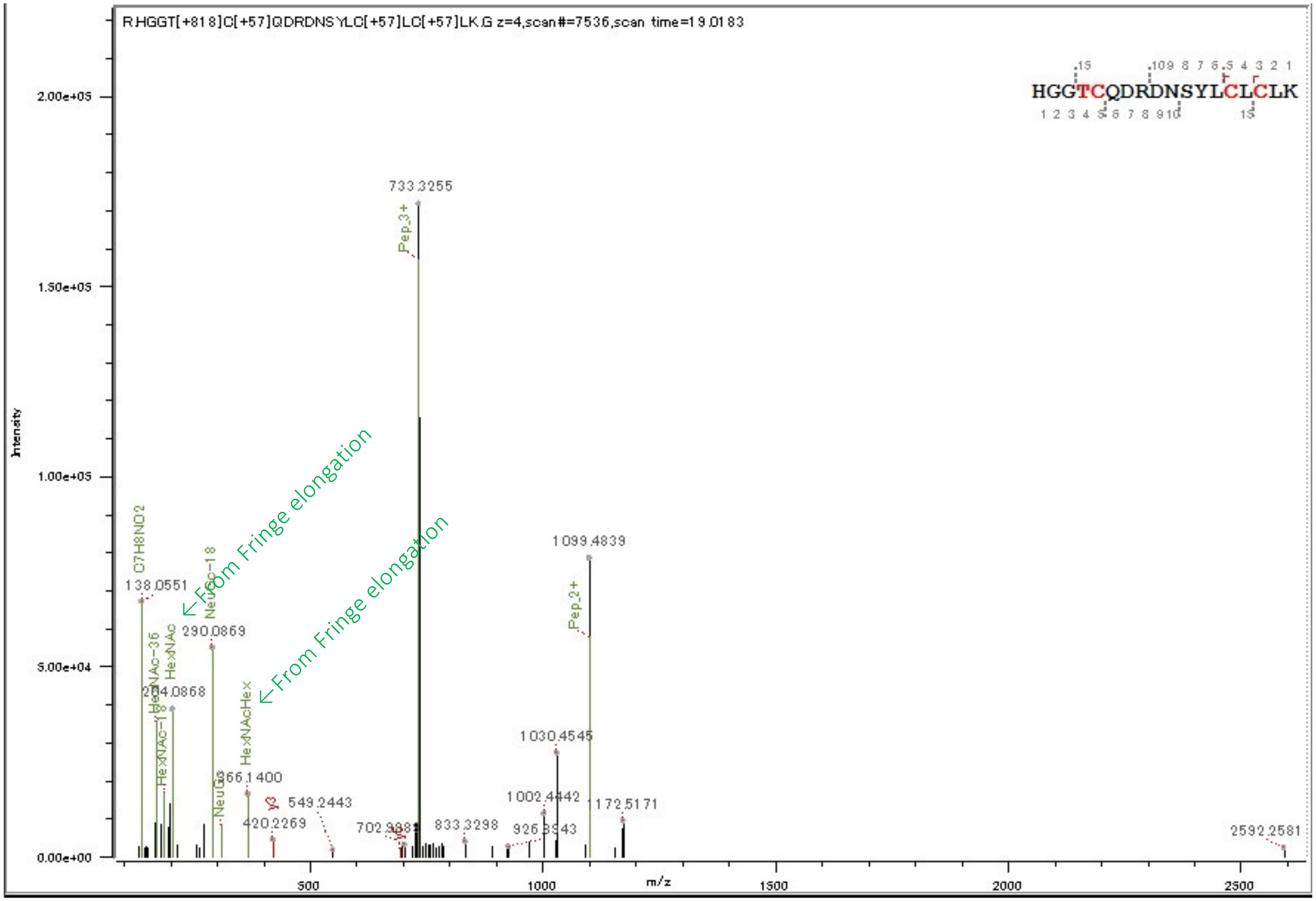

Figure S11K

Unmodified peptide of EGF18 from *Fng* LMR activated T cells

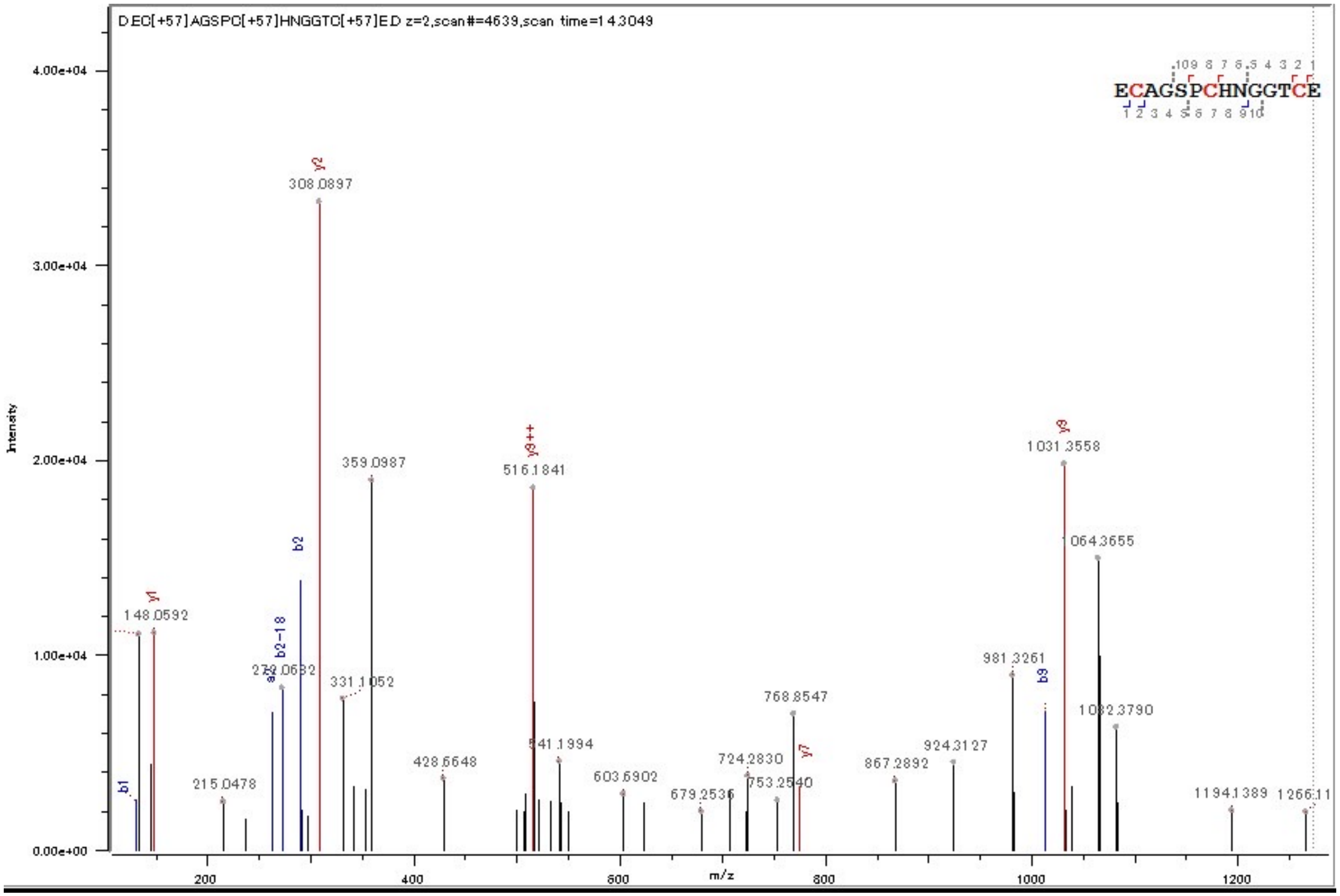

Figure S11L

Monosaccharide *O*-fucose modification of EGF21 from *Fng* LMR activated T cells

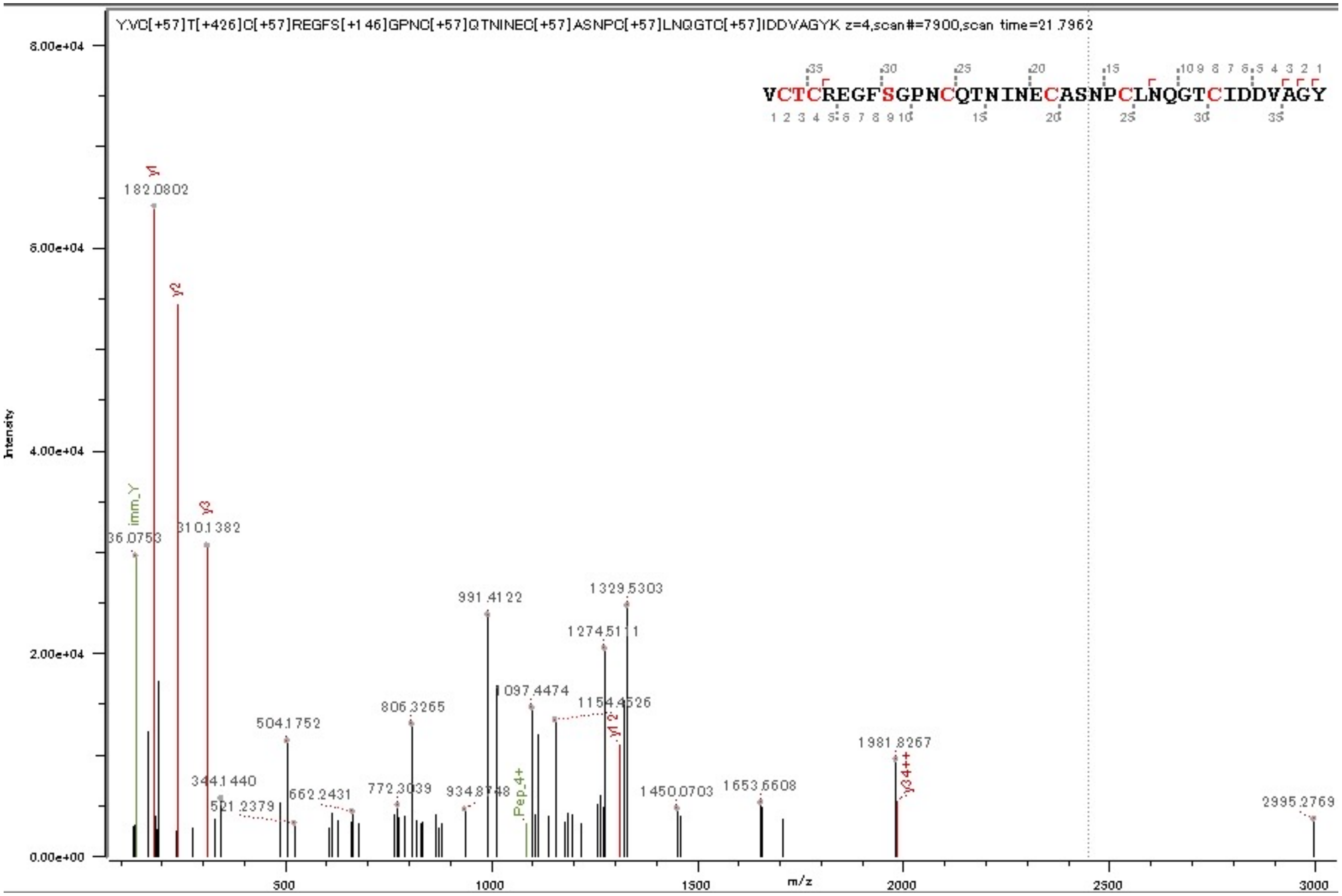

Figure S11M

Monosaccharide *O*-fucose modification of EGF23 from *Fng* LMR activated T cells

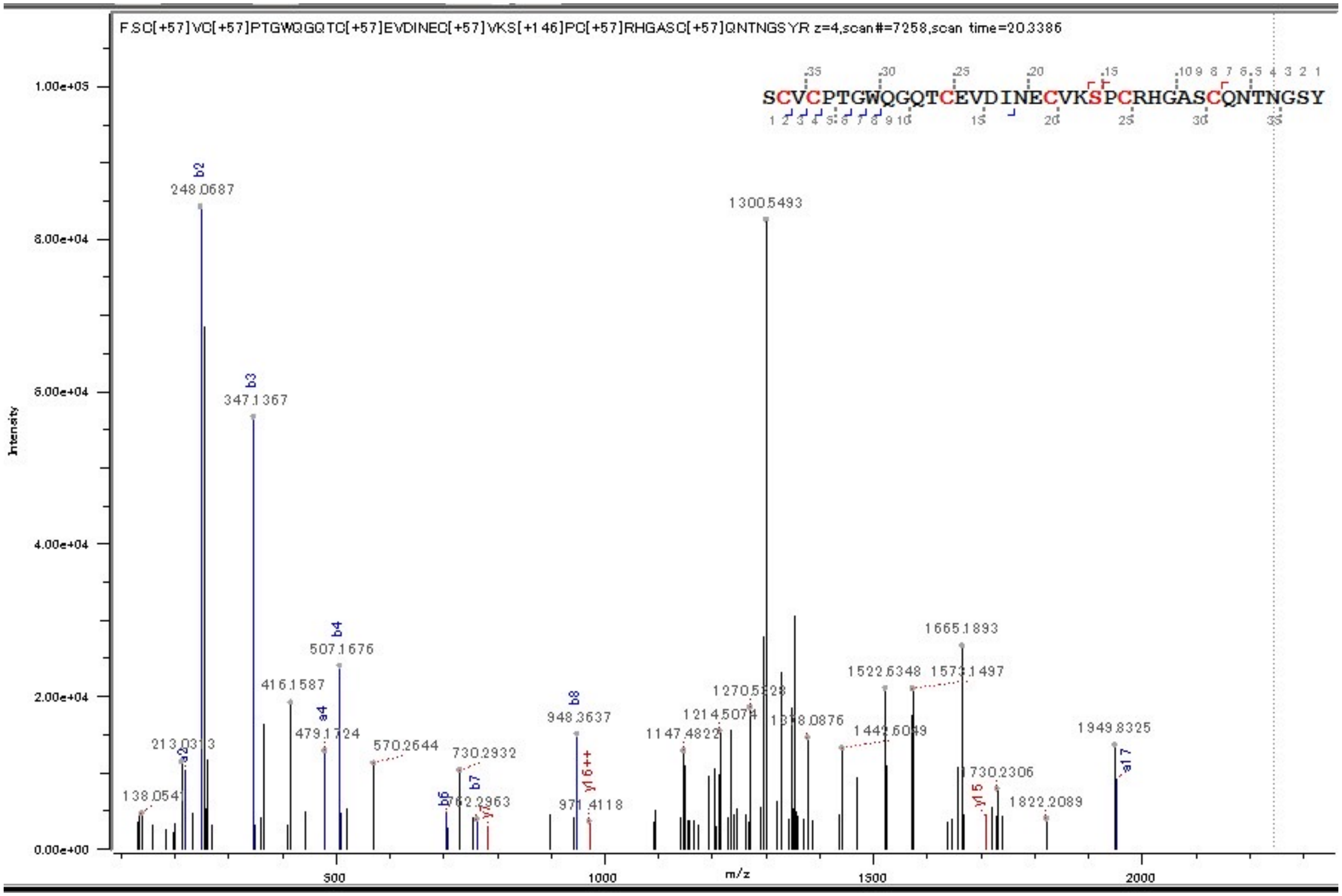

Figure S11N

Monosaccharide *O*-fucose modification of EGF26 from *Fng* LMR activated T cells

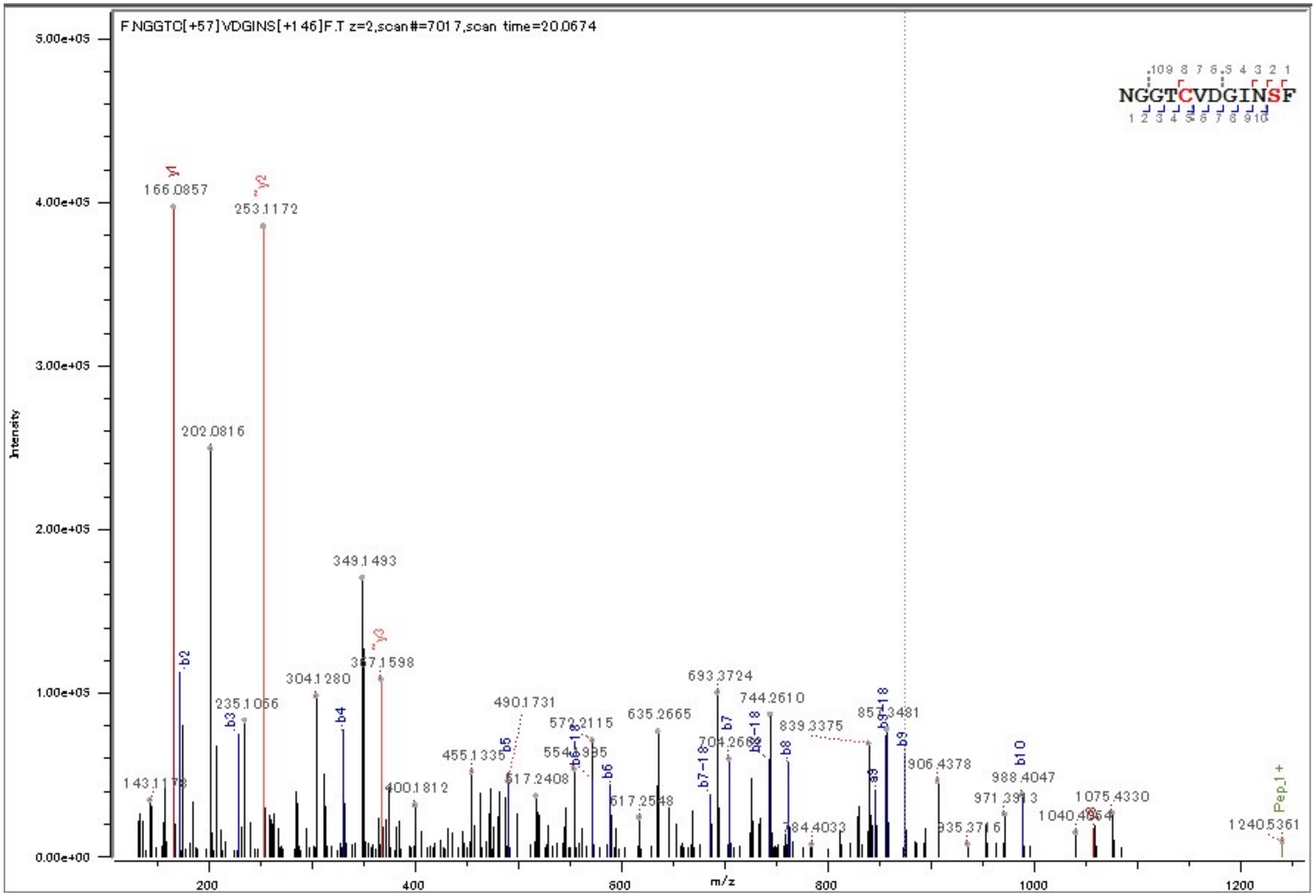

Figure S11O

Tetrasaccharide *O*-fucose (Fuc-HexNac-Hex-NeuAc) modification of EGF26 from *Fng* LMR activated T cells

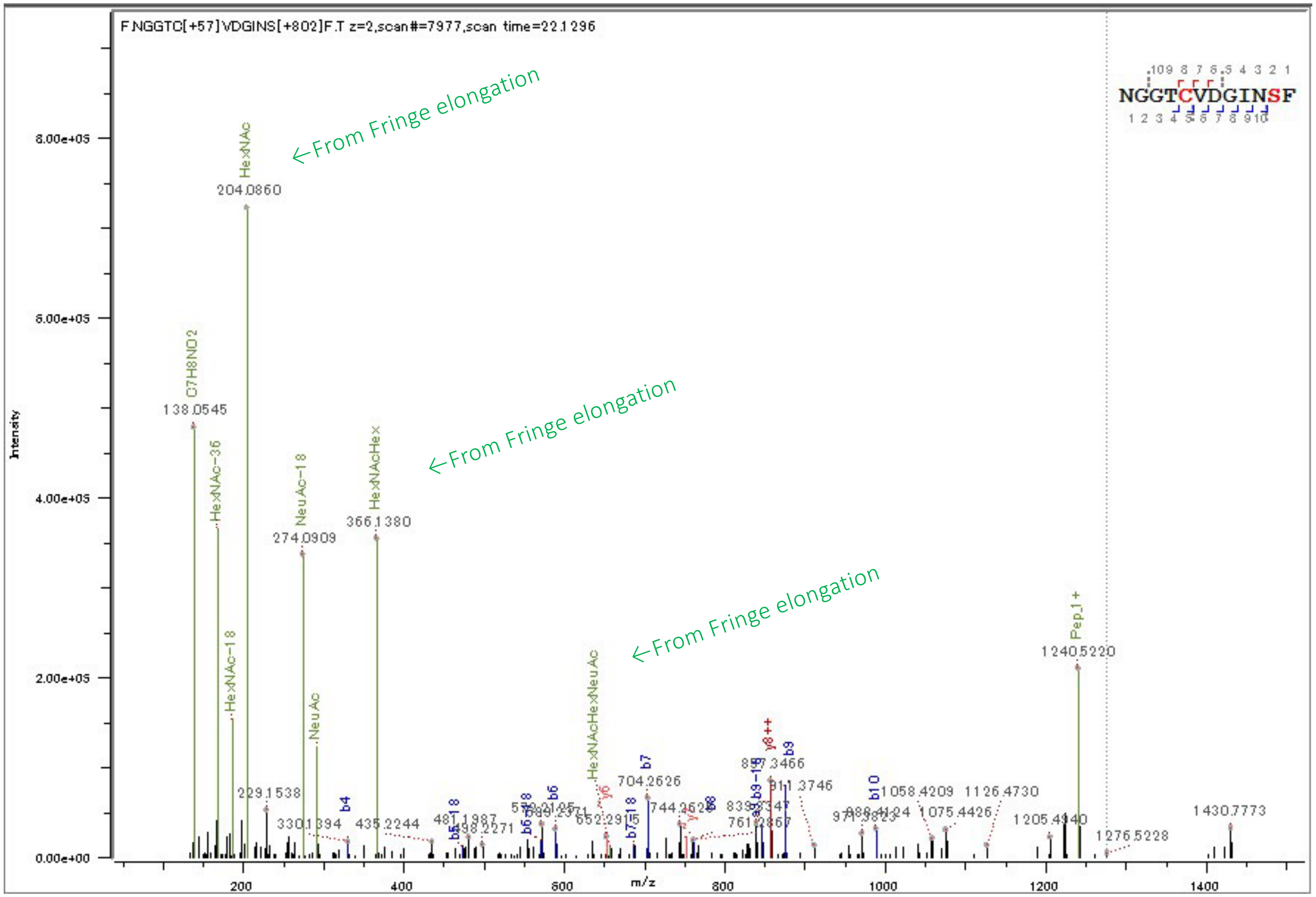

Figure S11P

Monosaccharide *O*-fucose modification of EGF27 from *Fng* LMR activated T cells

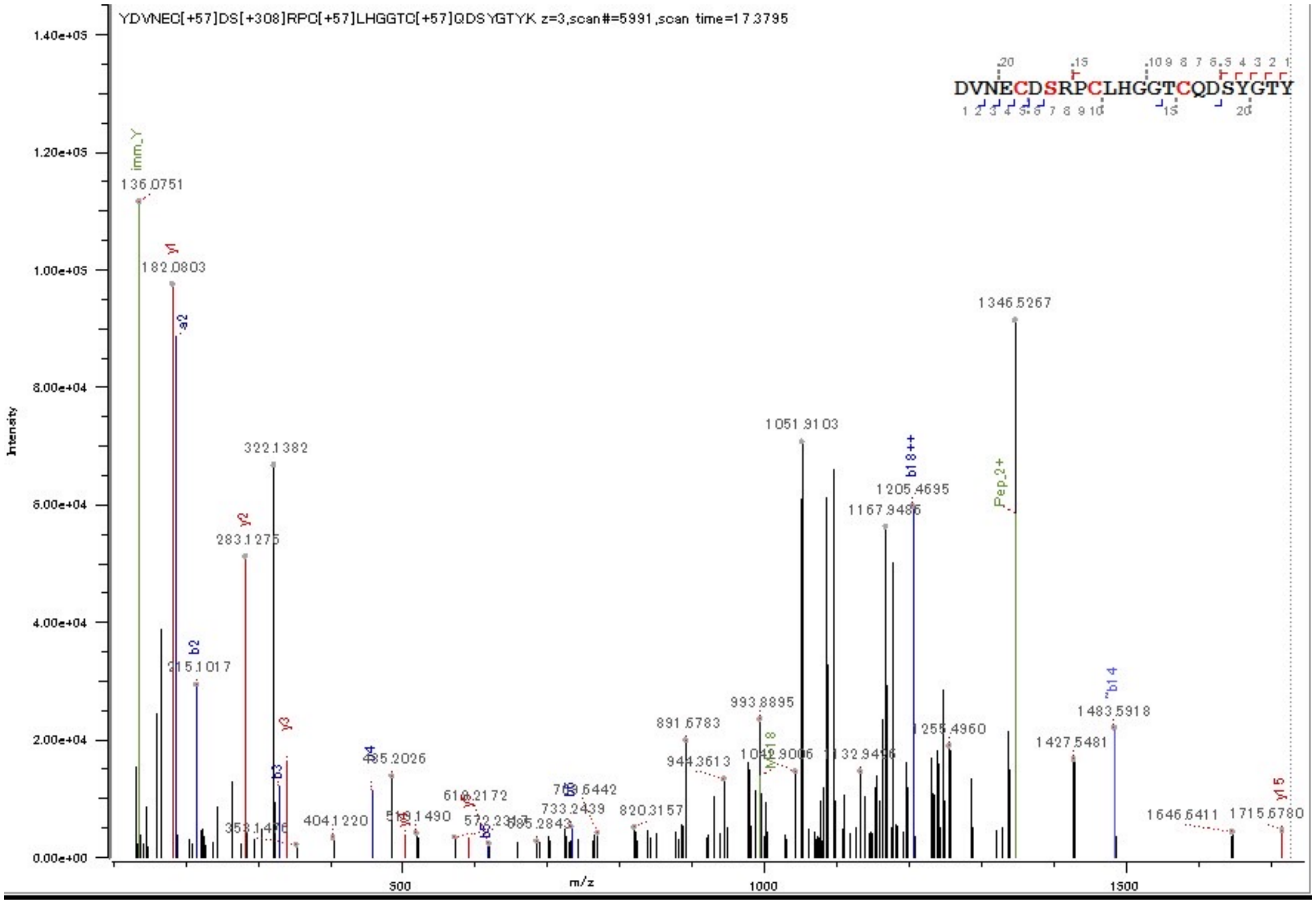

*This peptide is also modified with an *O*-glucose monosaccharide.

Figure S11Q

Disaccharide *O*-fucose modification of EGF27 from *Fng* LMR activated T cells

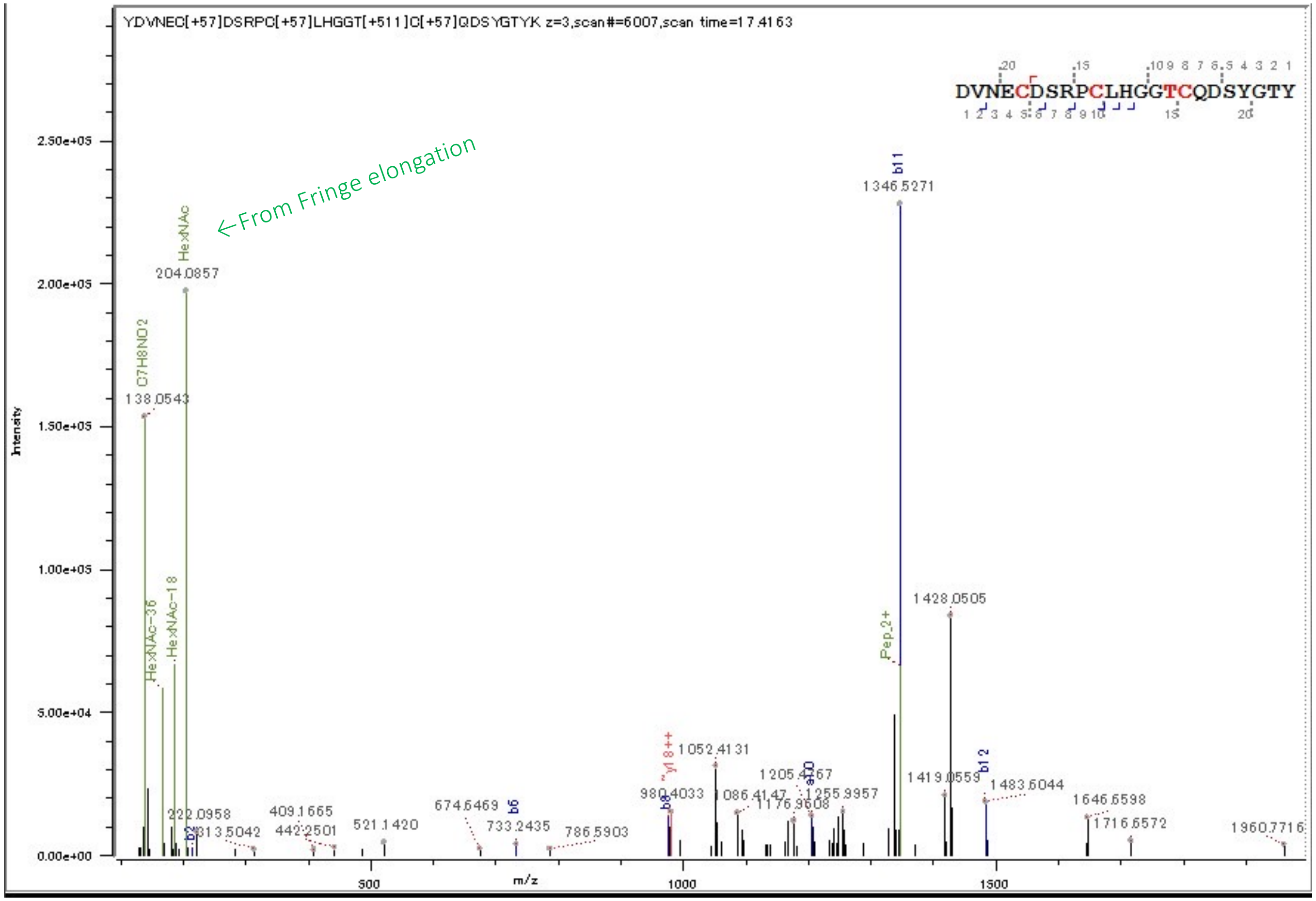

*This peptide is also modified with an *O*-glucose monosaccharide.

Figure S11R

Trisaccharide *O*-fucose modification of EGF27 from *Fng* LMR activated T cells

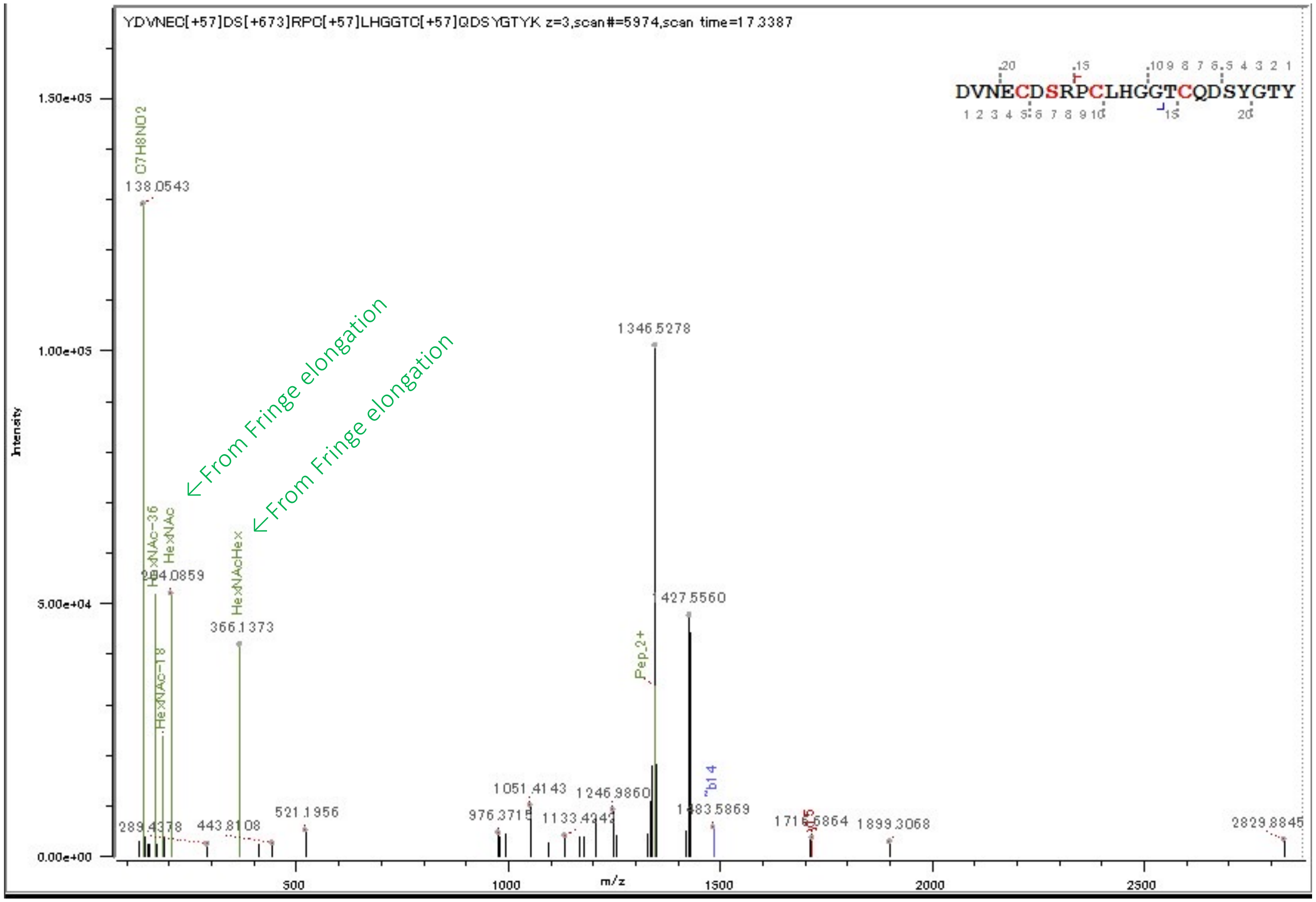

*This peptide is also modified with an *O*-glucose monosaccharide.

Figure S11S

Monosaccharide *O*-fucose modification of EGF35 from *Fng* LMR activated T cells

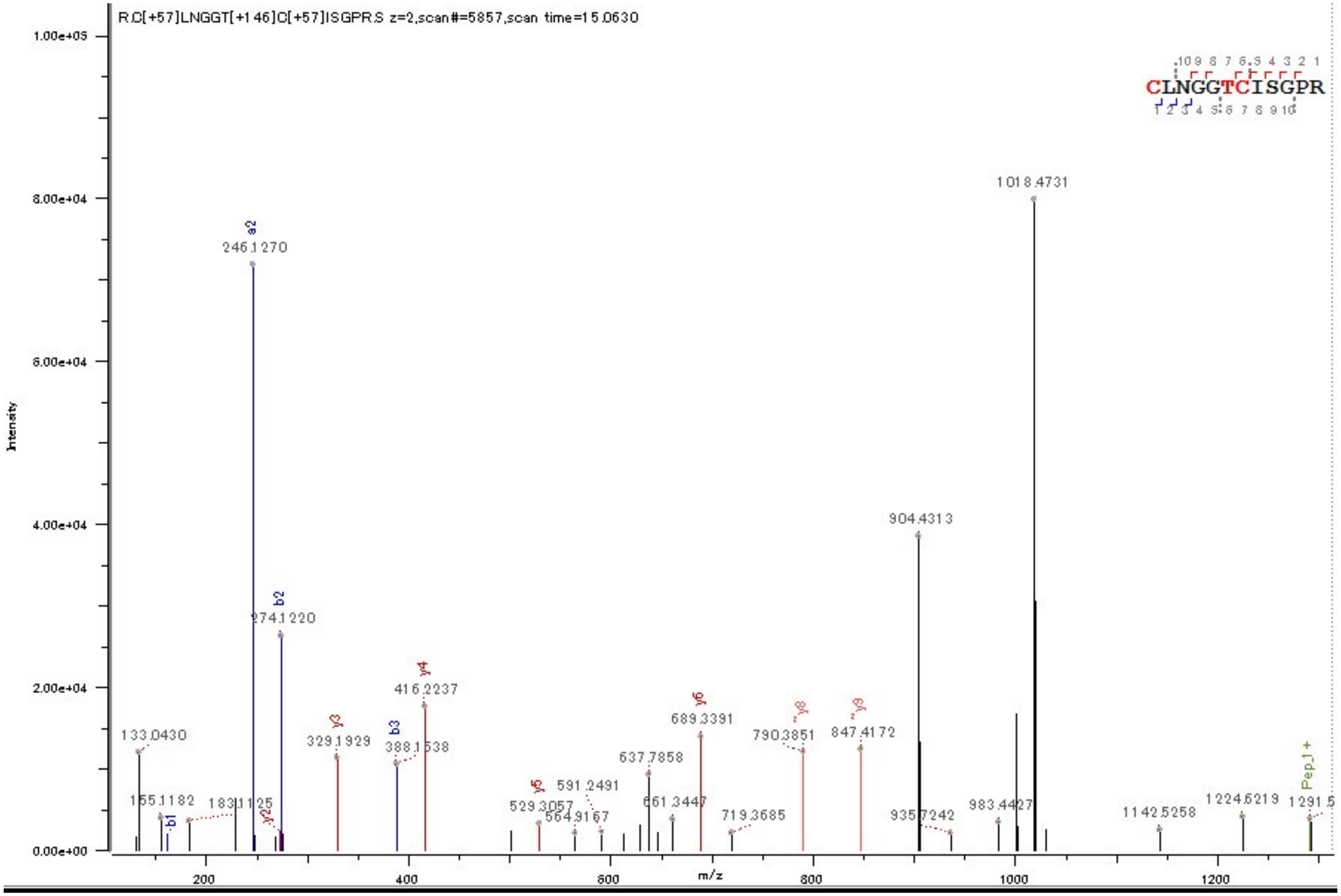

Figure S11T

Monosaccharide *O*-fucose modification of EGF36 from *Fng* LMR activated T cells

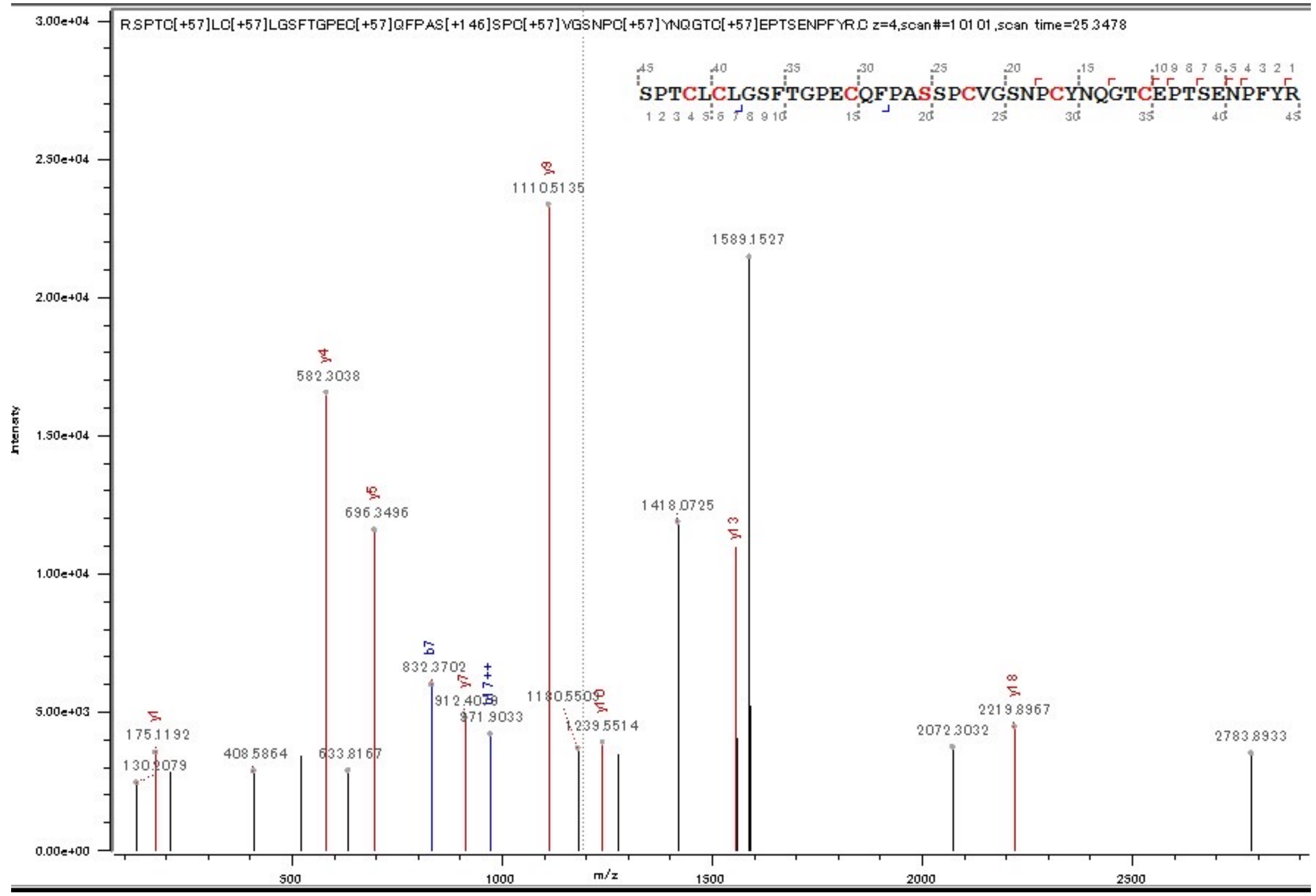

Figure S11U

Monosaccharide *O*-fucose modification of EGF36 from *Fng* LMR activated T cells

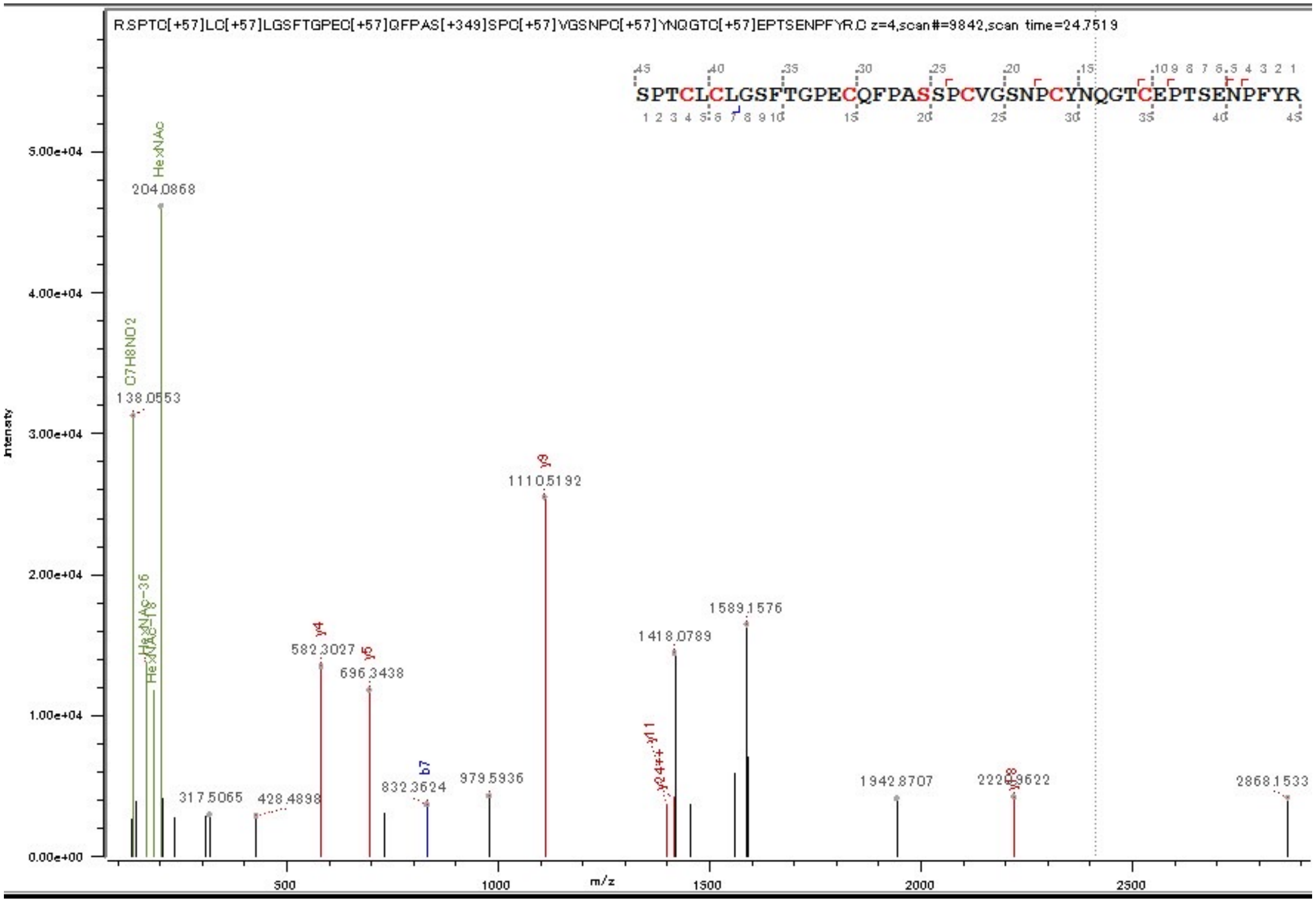

*This peptide is also modified with an *O*-GlcNAc monosaccharide

Figure S11V

Control peptide from *Fng* LMR activated T cells

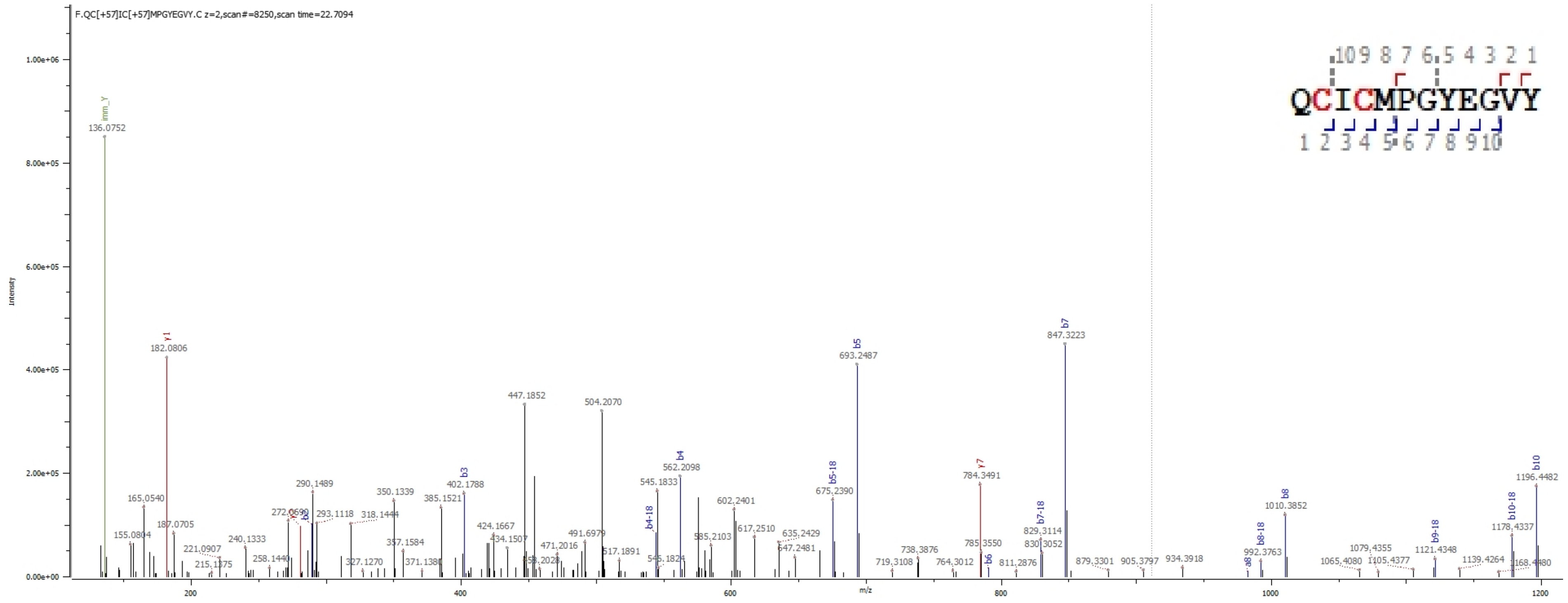

Figure S12A

Tetrasaccharide *O*-fucose(Fuc-HexNac-Hex-NeuGc) modification of EGF16 from *Lfng*-only activated T cells

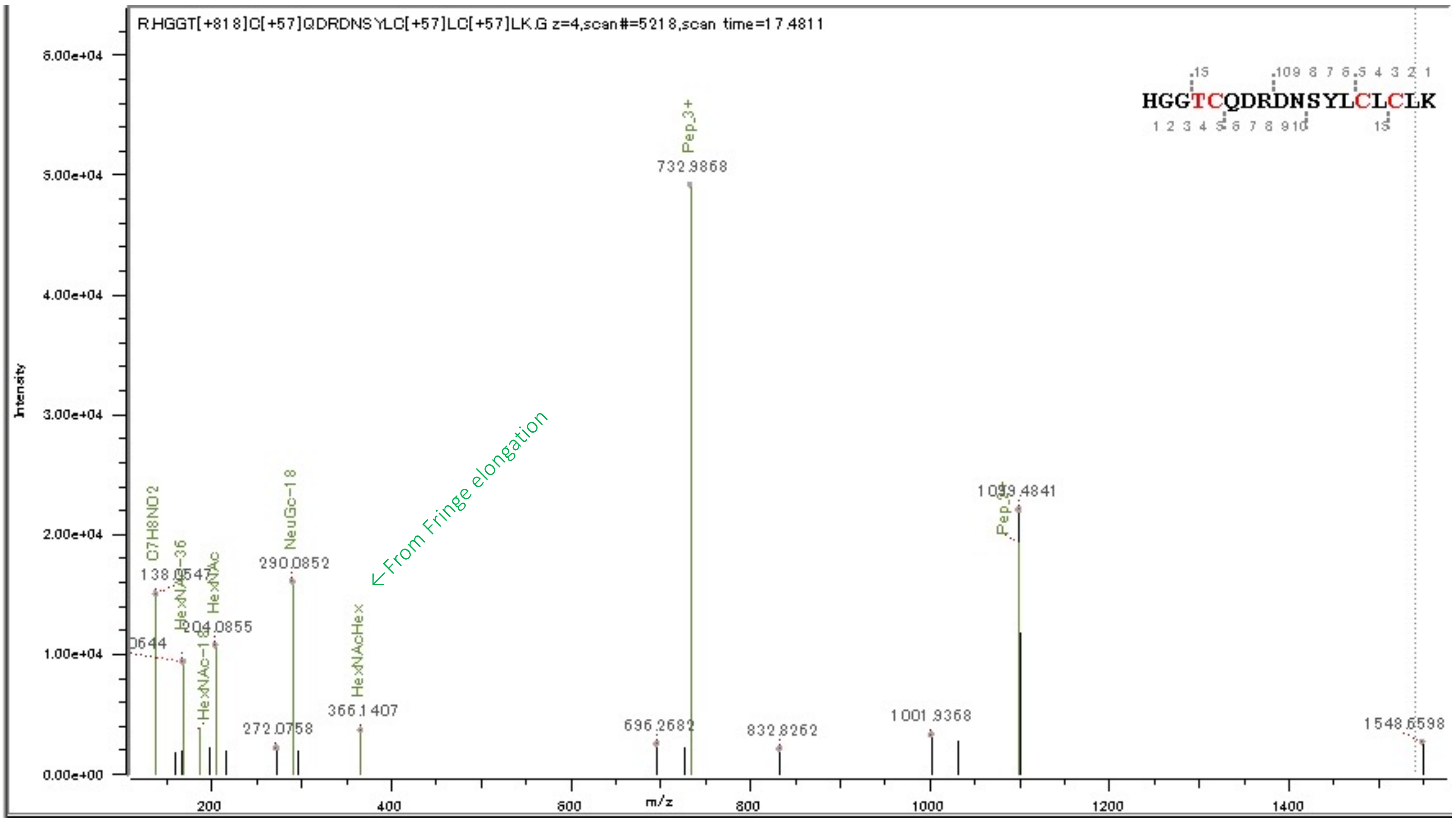

Figure S12B

Tetrasaccharide *O*-fucose(Fuc-HexNac-Hex-NeuGc) modification of EGF16 from *Rfng*-only activated T cells

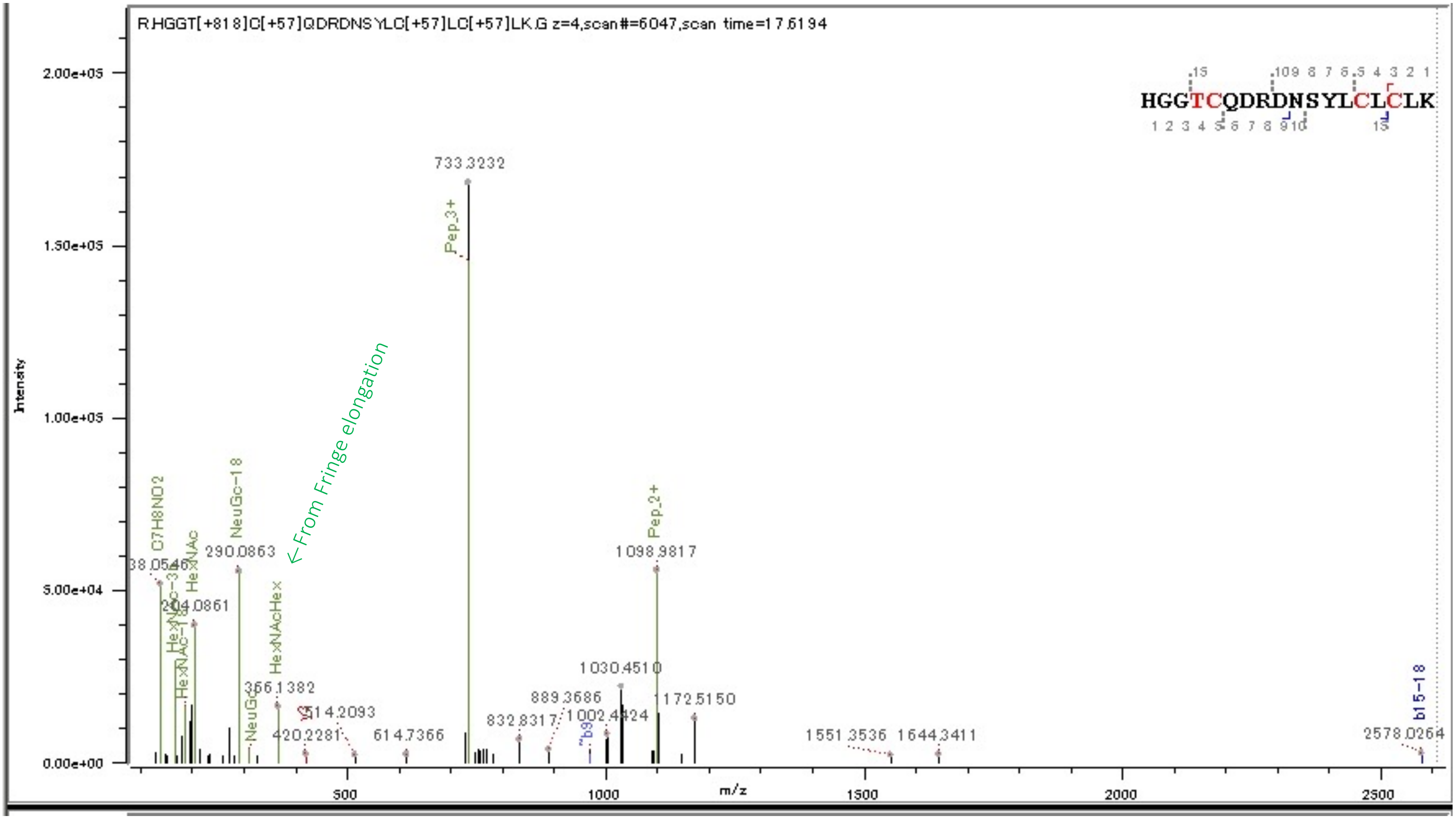

Figure S12C

Disaccharide *O*-fucose modification of EGF27 from *Lfng*-only activated T cells

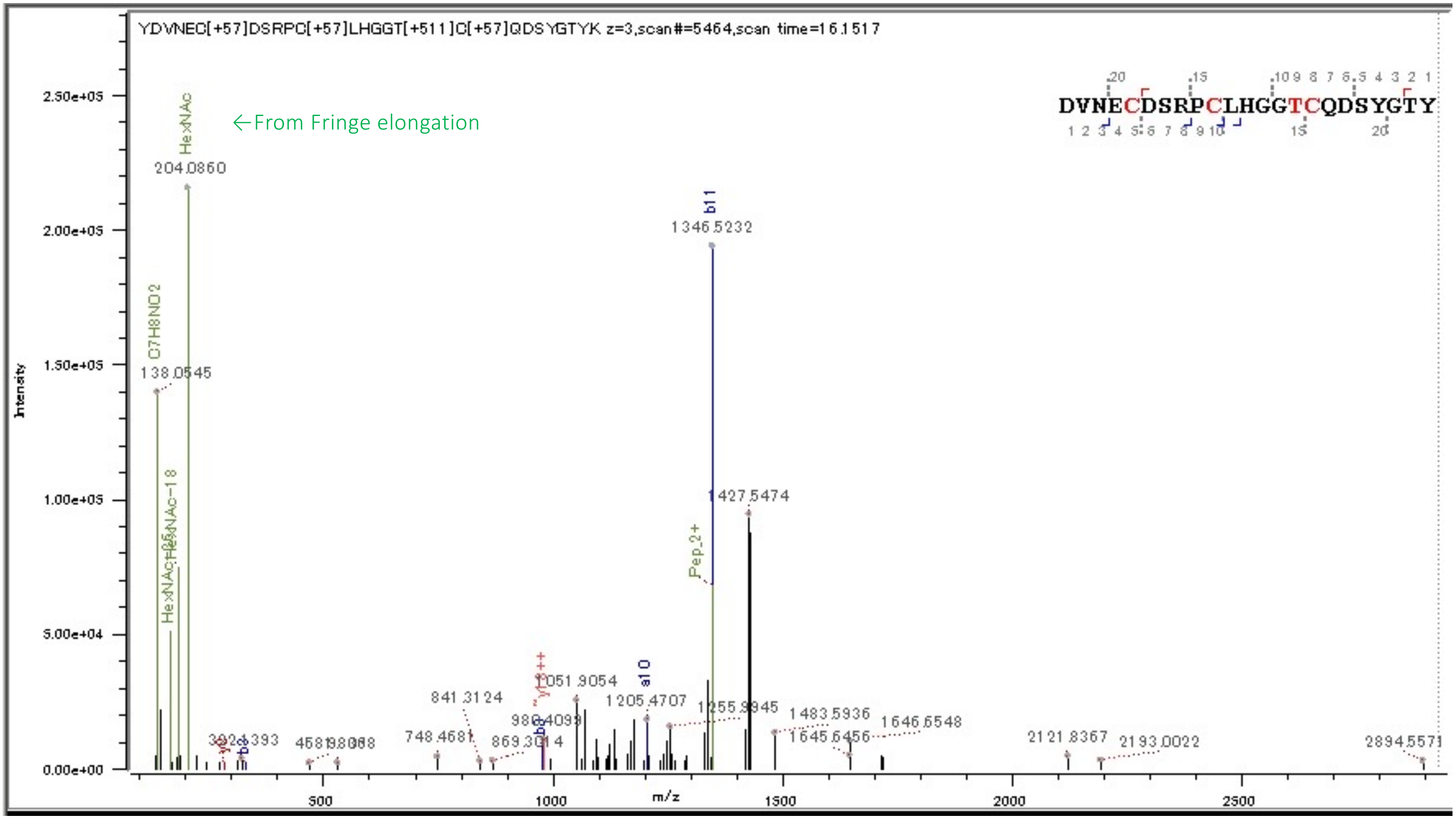

Supplement: Supporting Information [file mmc1.pdf]
